# Rational design of substituted maleimide dyes with tunable fluorescence and solvafluorochromism†
†Electronic supplementary information (ESI) available: Additional characterization and analysis of the maleimides. See DOI: 10.1039/c8cc00772a


**DOI:** 10.1039/c8cc00772a

**Published:** 2018-03-15

**Authors:** Yujie Xie, Jonathan T. Husband, Miquel Torrent-Sucarrat, Huan Yang, Weisheng Liu, Rachel K. O’Reilly

**Affiliations:** a Department of Chemistry, University of Warwick , Coventry , CV4 7AL , UK; b Department of Organic Chemistry I , Universidad del País Vasco (UPV/EHU) , and Donostia International Physics Center (DIPC) , Manuel Lardizabal Ibilbidea 3 , Donostia 20018 , Spain; c Ikerbasque , Basque Foundation for Science , María Díaz de Haro 3, 6o̲ , Bilbao 48013 , Spain; d Key Laboratory of Nonferrous Metals Chemistry and Resources Utilization of Gansu Province and State Key Laboratory of Applied Organic Chemistry , Lanzhou University , Lanzhou 730000 , P. R. China; e School of Chemistry, University of Birmingham , Edgbaston , B15 2TT , UK . Email: r.oreilly@bham.ac.uk

## Abstract

A series of maleimide derivatives were systematically designed and synthesized with tunable fluorescent properties. The facile modifications herein provide a simple methodology to expand the scope of maleimide-based dyes and also provide insight into the relationship between substitution pattern and optical properties.

## Abstract 2

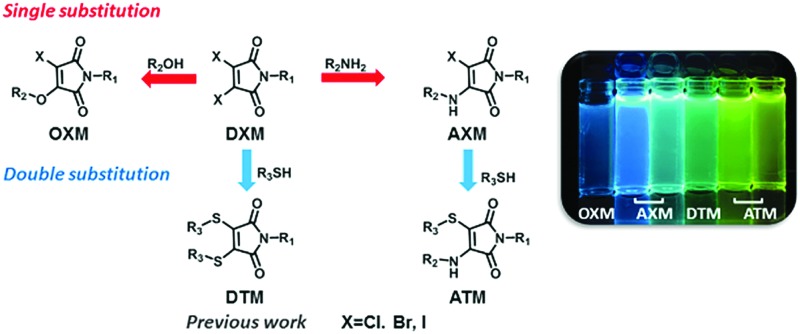
We herein present a simple methodology to systematically expand the scope of maleimide-based dyes and also provide an insight into the relationship between substitution pattern and optical properties.

The development of novel organic fluorophores is of great interest in the areas of biological labels and probes,^1^ chemosensors,^2^ medical diagnostics^3^ and photoactive materials.^4^ Indeed, the rational design of organic fluorophores has provided a straightforward method for generating various fluorescent functional materials.^5^–^7^ Currently, many bulky planar fluorophores suffer from complex syntheses, large size and poor solubility, limiting their applications in fluorescence labelling and functionalization. Among reported organic dyes, maleimide-based fluorophores, one of the smallest fluorophores, exhibit promising properties, such as high emissivity, large Stokes shifts, and ease of modification.^8^–^13^

Unsubstituted maleimides have been extensively reported as effective fluorescence quenchers through direct conjugation to fluorophores.^14^–^16^ The low lying n–π* transition of the maleimide ring can provide a nonradiative pathway for excited state decay and significantly reduce the emission of fluorophores.^17^–^19^ Recently, our group realized two novel fluorescent maleimide dyes: dithiomaleimides (**DTM**)^20^ and aminobromomaleimides (**ABM**),^21^ which exhibit strong emission originating from their donor–acceptor architectures. The main advantage of these maleimide based dyes is their small size, which allows for ready incorporation into architectures without affecting or disrupting the scaffold.^22^ Therefore, such maleimides have subsequently been used as ideal fluorescent linkers in polymer functionalization and protein labelling.^20^,^23^–^27^ The Baker and Caddick groups have extensively investigated these small molecules in the labelling of thiol or disulfide containing antibodies and peptides.^28^–^30^ In addition, the versatile and facile chemistry in the maleimides synthesis make it easy to tailor towards a specific application. Our group first utilized substituted maleimides in radical polymerizations as a labelling technique that did not require protection of double bond.^31^ By this method, the maleimide can be incorporated into the interface of amphiphilic copolymers where the fluorescence output is dependent on polymer assembly.^32^ Furthermore, the solvatofluorochromic properties of maleimide dyes have been utilized to create responsive particles, which exhibit reversible on/off fluorescence emission in the presence of CO_2_ or N_2_.^33^

Despite the recent advances in this field, there has been little work on varying the maleimide structures and studying the subsequent impact on their fluorescence.^8^–^13^,^21^,^34^,^35^ The search for a versatile maleimide fluorophore is pivotal in this area, where structures with well-defined reactivities and varying fluorescent profiles are needed. In this work, we systemically expanded the scope of substitution patterns, through single or double substitution reactions from halogen precursors for the first time. Moreover, we explored the relationship between the substitution patterns and their optical properties. In this regard, the fluorescent properties of maleimide structures could be predicted and tuned for various applications.

The design concept relies on the typical electron donor and acceptor mechanism in maleimide-based fluorophores where the C=O groups and the group on the C=C moiety act as the electron acceptor and donor respectively. Our initial strategy began with reacting three different pro-fluorescent precursors: dibromo (**DBM**), dichloro (**DCM**) and diiodo (**DIM**) maleimide (**1a–c**, Scheme 1). The conversion of dibromomaleimide (**DBM**) to aminobromomaleimide (**ABM**), through single substitution, with an amine, has been previously reported by our group.^21^

**Scheme 1:**
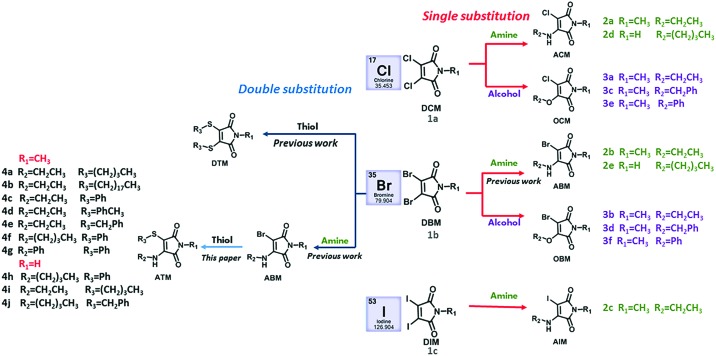
Design and structures of single- and double-substituted maleimides.

Based on this method, we expanded the scope by reacting the amine with other halogeno maleimides (**DCMs** and **DIMs**). The reaction resulted in the formation of a singly substituted product for all precursors. The optical properties of these three different amino substituted halogeno maleimides (**2a–c**) were investigated. In diethyl ether, the three compounds (**2a–c**) exhibited two similar absorption peaks around 238 nm and 370 nm assigned to an n–π* and a π–π* transition, respectively (Fig. 1a and Table S1, ESI†).^36^ Both aminochloromaleimide (**ACM**, **2a**) and aminobromomaleimide (**ABM**, **2b**) showed similar green emission *ca.* 475 nm while the aminoiodomaleimide (**AIM**, **2c**) produced a slightly red shifted emission (*ca.* 487 nm). The fluorescence quantum yields (*Φ*_f_) of these products were measured using quinine sulfate (59%, in 0.105 M HClO_4_) as a reference, to establish any effect of the halogen.^37^ The *Φ*_f_ decreased from Cl (**2a**, 37%) through Br (**2b**, 30%) to I (**2c**, 8%) in diethyl ether (Fig. 1b and Table S1, ESI†), matching the trend in decreasing electronegativity. This suggests that a more electron withdrawing halogen results in an increase in the fluorescence intensity and *Φ*_f_ as a consequence of lower electron density on the donor nitrogen atom. To confirm this hypothesis, we synthesized a previously reported highly emissive **ABM** (**2e**) and compared this to the analagous chloro derivative (**2d**).^21^ The *Φ*_f_ of **2d** was 42% in diethyl ether and 65% in cyclohexane, which is the highest *Φ*_f_ of all reported amino halogeno maleimides.

**Fig. 1:**
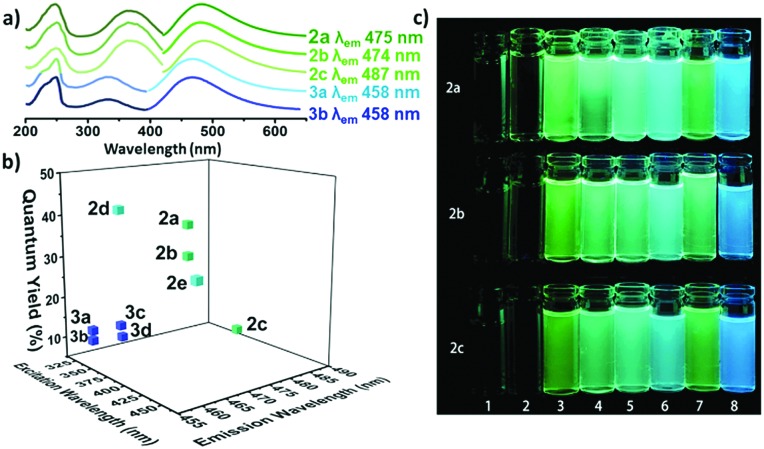
(a) UV and emission spectra of fluorescent aminomaleimides (**2a–c**) and alkoxybromomaleimides (**3a–b**); (b) fluorescence quantum yields of the singly substituted maleimides (**2a–e**, **3a–d**) against excitation and emission wavelengths (recorded at 10 μM in diethyl ether); (c) aminomaleimides (**2a–c**) in 8 solvents (1–8: H_2_O, MeOH, DMF, dioxane, THF, Et_2_O, CH_2_Cl_2_ and cyclohexane) under UV light (365 nm).

The solvatofluorochromic properties were further investigated in solvents across a wide polarity range. All compounds (**2a–c**) exhibited a similar change in fluorescent emission across the solvent series (Fig. 1c), in that the *Φ*_f_ of all were reduced in protic solvents (methanol, water) and the emission wavelengths were shifted to higher wavelengths. This is consistent with previous work which shows that the hydrogen bonding between protic solvents and the C=O group in maleimides causes quenching effects through electron driven proton transfer from the solvent to the fluorophore.^38^ In non-polar solvents such as cyclohexane, bright emission with the highest *Φ*_f_ was observed, likely as a consequence of the suppression of twisted intramolecular changer transfer (TICT) in the maleimide rings.^9^

Looking at these results it was clear that the optical properties are closely related to the electronegativity of the halogen substituents, with increased electronegativity correlating with increased *Φ*_f_. However, the effect of the donor group in these systems has not yet been widely explored. Hence, this was investigated by replacing the strong electron donating amino group with a weakly electron donating alkoxy group (**OCM** and **OBM**, **3a–f**). Following the route of Booker-Milburn *et al.*, sodium alkoxide was used to catalyze a substitution reaction with **DCM** and **DBM** and a series of alcohols.^29^ The *in situ* formation of the products, **3a–f**, proceeded successfully for both aryl and alkyl alcohols at room temperature.

In the absorbance spectra of **3a–d**, the typical π–π* transition peak shifted from *ca.* 360 nm (as observed for the amino derivatives, **2a–e**) to *ca.* 335 nm, while also reducing in intensity (Fig. 1a and Fig. S5, ESI†). Meanwhile, the n–π* transition at 230 nm increased in intensity which likely correlates to a non-radiative pathway and results in a decrease in fluorescence. Moreover, changing the substituent from ethylamino (**2b**) to an ethoxy (**3b**) group also lead to a dramatic decrease in *Φ*_f_ from 30% to 10% (in diethyl ether). This indicates that a strong electron donating group on the C=C bond is critical in obtaining a high fluorescence intensity. Looking at the series in more detail, both the **OCM** and **OBM** substituted ethoxy (**3a–b**) and benzyloxy (**3c–d**) maleimides showed similar emission wavelengths, whereas no emission was observed for the phenoxy (**3e–f**) derivatives (Table S1, ESI†). These results are consistent with our previous reports, in which the direct conjugation of aromatic rings leads to emission quenching in **ABMs** and **DTMs**.^20^,^21^ Moreover, the same effects of varying the halogen from **OBM** to **OCM** were observed as in the amino substituted series (**ACM***vs.***ABM**). As the optical properties are closely related to modifications made to this series of singly substituted maleimides, it was hypothesized that more remarkable changes may be realized by exploring disubstituted maleimides. Previously, it has been demonstrated that **DBMs** were capable of undergoing an efficient addition-elimination reaction with thiols resulting in a disubstituted product using thiol groups.^39^ To expand upon these, unsymmetrical disubstituted products which contained an amine and a thiol group were targeted. Awuah and Capretta previously managed to introduce a second amino moiety onto an **ABM** scaffold using microwave irradiation at 50 °C.^40^ They determined that the amino group in **ABMs** altered the electronic nature of the neighbouring site, such that a second amine addition does not occur at room temperature. Conversely, we were inspired by the fast and efficient double addition elimination reaction with thiols and hence attempted to introduce a second thiol group on an **ABM**. A similar compound has been reported to date, by Naka *et al.*, where thiophenol was able to undergo reaction with an aniline substituted maleimide. However, due to the aromatic nature of the aniline, almost no fluorescence was observed in solution.^34^ Herein, a range of aminothiomaleimides (**ATM**) were synthesized and their optical properties explored. **ATMs** were generally synthesized from **ABMs** through the addition of 1 equivalent of thiol and sodium hydroxide at 80 °C in DMF. Following the successful synthesis of a series of ten **ATMs** (**4a–j**), the optical properties were characterized. The excitation wavelength of the **ATM** compounds remained in the 360–380 nm range (in diethyl ether) similar to **ABMs**, while the emission shifted to higher wavelengths (520–560 nm in diethyl ether, Fig. S6, ESI†), which was observed as yellow emission under UV light (Fig. 2a). This results in a larger Stokes shift (*ca.* 147–165 nm, *ca.* 296–329 nm in diethyl ether) compared with previous substituted maleimides, which could make them useful in Förster Resonance Energy Transfer (FRET) based applications. The solvafluorochromic properties of **ATM** (**4h**) was compared with **ABM** (**2e**) in eight common solvents (Fig. S13, ESI†). Compared with the blue to green emissions of **ABM** (**2e**), the emissions of **ATM** (**4h**) were higher in wavelength (green to yellow range). Similar with **ABMs**, fluorescence quenching of **ATM** was observed in protic solvents which is attributed to hydrogen bonding between the protic solvent and the carbonyl.

**Fig. 2:**
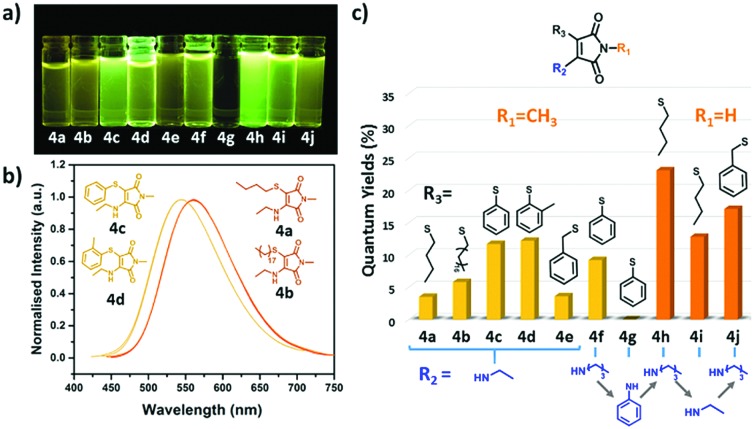
(a) Solution of **ATMs** under UV light (365 nm); (b) emission spectra of different alkyl thiol (**4a–b**) and thiophenol (**4c–d**) aminomaleimides (recorded at 10 μM in diethyl ether); (c) fluorescence quantum yields of studied **ATMs** in diethyl ether.

In order to explore how the thiol group influences the fluorescence, **ATMs** with varying thiol substituents (**4a–d**) were compared. The emission of two alkyl thiol substituted compounds (**4a** and **4b**) showed a relatively high emission wavelength (*ca.* 560 nm) which approached the red region of the spectrum (Fig. 2b), however, these products showed poor emission intensity and low *Φ*_f_ (<10%, Fig. 2c and Table S1, ESI†). Interestingly, when switching to a thiophenol group (**4c** and **4d**), the emission maxima blue-shifted with an unexpected increase in quantum yield. Furthermore, strong emission was also observed in the solid state of thiophenol functionalized **ATM** (**4h**) compared to their analogous **ABM** (**2e**). Comparing the absolute *Φ*_f_ in solid states, **4h** processes a higher *Φ*_f_ (**4h** 8% *versus***2e** 1%) and longer fluorescence lifetime (**4h** 2.97 ns *versus***2e** 0.65 ns; Fig. S15, ESI†), suggesting that the twisted benzene ring can be retained in the solid state and this diminished aggregation caused quenching (ACQ) in the solid state. To study this further, the fluorescence of **4h** and **2e** were compared in a mixed solvent system of hexane (non-solvent) and dichloromethane (good solvent). The fluorescence of **4h** gradually increased with the addition of non-solvent while aminomaleimide **2e** did not present the same trend (Fig. S16, ESI†). These results suggest that quenching in the solid state can be prevented by introducing an aromatic thiol adjacent to the donor group resulting in dual-state emission (DSE).^36^ This trend aligns with previous reports in which maleimides with three aromatic motifs showed aggregation induced emission (AIE).^9^,^34^

Following the study on the effect of thiol groups in the R3 position, substituents on the R1 and R2 positions were also found to affect the optical properties significantly (Table S1, ESI†) in the **ATMs**. Specifically, compounds with a hydrogen at the R1 position (**4h–j**) showed an increased Φ_f_ compared with compounds with methyl at R1 (**4a–g**). As expected, the direct conjugation of an aniline group at the R2 position (**4g**) also caused dramatic quenching of the fluorescence (*Φ*_f_ < 0.1%), consistent with previous reported aminobromomaleimides.^21^ These results indicate that the fluorescence of **ATMs** originates from the aminomaleimide moiety analogous to **ABMs**, in which the amino group on C=C double bond acts as the donor moiety in a donor–acceptor skeleton.

To confirm the conclusions derived from this work, the lowest-energy absorption and emission peaks were computed at CAM-B3LYP-D3BJ(PCM)/6-311G(d,p) level using time-dependent density functional theory (for more details see ESI†). All the energies displayed in Table S2 (ESI†) correspond to HOMO (π) → LUMO (π*) transitions (Fig. S17–S22, ESI†). Moreover, a natural population analysis of the carbon atoms adjacent to the electron withdrawing and donating groups (labelled as C2 and C3, respectively) confirmed the electron displacements indicated throughout the text (Table S3, ESI†). It is worth noting that the high *Φ*_f_ values are usually associated with small charge differences between C2 and C3 atoms, reinforcing the push–pull model that to increase the *Φ*_f_ it is necessary to introduce strong electron donating and withdrawing groups into the maleimide scaffold.

In conclusion, we successfully synthesized a library of maleimide fluorophores with remarkable fluorescent properties such as tunable emissions among the visible colour range (blue to yellow), high fluorescence quantum yields (up to 64%) and solvafluorochromism. Significantly, we investigated the effect on optical properties when altering the halogen group (Cl, Br and I) and the donor groups (amines and alcohols). The fluorescence results provide evidence of the previously hypothesized push–pull model and computational results further confirmed that smaller charge differences on the C=C bond positively affect fluorescence. Building upon this work and further exploring the relationship between the structure of maleimides and their optical properties is essential for full understanding of the fluorescence mechanism. This, in turn, will aid the design of future maleimide fluorophores with small size, multi-functionalization and high fluorescence efficiency for biological and chemical sensing applications.

The authors thank the University of Warwick, the ERC (grant no. 615142), Ministerio de Economía y Competitividad (MINECO) of Spain (project CTQ2016-80375-P) and EPSRC for financial support. The authors acknowledge the computational resources and technical and human support provided by DIPC.

## Conflicts of interest

There are no conflicts to declare.

## Supplementary Material

Supplementary information
