# Supplementary material for: Rational design of substituted maleimide dyes with tunable fluorescence and solvafluorochromism
Source: Chem Commun (Camb). 2018 Mar 15;54(27):3339–42. doi: 10.1039/c8cc00772a (PMC5885783; doi:10.1039/c8cc00772a)
Supplement: Supplementary file 1 [file CC-054-C8CC00772A-s001.pdf]

Electronic Supplementary Material (ESI) for ChemComm.
This journal is © The Royal Society of Chemistry 2018

Electronic Supplementary Information

For

Rational Design of Substituted Maleimide Dyes with Tunable Fluorescence and Solvafluorochromism

Yujie Xie,[a] Jon Husband,[a] Miquel Torrent-Sucarrat,[b,c] Huan Yang,[d] Weisheng Liu[d] and Rachel K. O'Reilly,[a,e]*

[a] Department of Chemistry, University of Warwick, Gibbet Hill Road, Coventry, CV4 7AL, UK.

[b] Department of Organic Chemistry I, Universidad del País Vasco (UPV/EHU), and Donostia International Physics Center (DIPC), Manuel Lardizabal Ibilbidea 3, 20018 Donostia, Spain.

[c] Ikerbasque, Basque Foundation for Science, María Díaz de Haro 3, 6º, 48013 Bilbao, Spain.

[d.] Key Laboratory of Nonferrous Metals Chemistry and Resources Utilization of Gansu Province and State Key Laboratory of Applied Organic Chemistry, Lanzhou University, Lanzhou 730000, P. R. China

[e.] School of Chemistry, University of Birmingham, Edgbaston, BT15 2TT, UK. E-mail: r.oreilly@bham.ac.uk

Contents

1. Materials and apparatus S3

2. Chemical structures of maleimides and synthetic protocols S4

Chemical structures of maleimides S4

Experimental procedure for di-halogen maleimides preparation . S5

General procedure of aminomaleimides synthesis SError! Bookmark not defined.

General procedure for alkoxymaleimides synthesis S8

General procedure for thioaminomaleimide synthesis: S10

3. Fluorophore characterization S15

4. UV-Vis Spectrum of maleimides S17

5. Solvatochromic determination S19

6. Photographs in different solvents under UV light S21

7. DSE effect of second thiol substituted amino maleimides S22

8. NMR spetra of substituted maleimides S23

9. Computational Details S44

10. References S67

1. MATERIALS AND APPARATUS

All chemicals and reagents were purchased from either: Sigma Aldrich, Fisher Chemicals, Acros Chemicals or Alfa Aesar and used as received. Dithiolmaleimides was synthesized according to a previous paper.[1] Solvents were purchased from Fisher Scientific and used as received. Dry solvents were used directly from a drying and degassing solvent tower delivery system.

NMR spectra were recorded on a Bruker Avance 300, a Bruker Avance III HD 400 or a Bruker Avance III HD 500 spectrometer at 298k and 300, 400 and 500 MHz, respectively. Shifts are quoted in δ in parts per million and quoted relative to the internal standard trimethylsilane (TMS). High Resolution Mass Spectra (HR-MS) were conducted on a Bruker UHR-Q-ToF MaXis spectrometer with electrospray ionization. Infrared spectra were recorded (neat) on a PerkinElmer 100 FT-IR, UV-Vis spectroscopy was carried out on a Perkin Elmer Lambda 365 UV/vis spectrometer or an Agilent Cary 60 UV-Vis Spectrometer at room temperature. Fluorescence spectra were recorded using an Agilent Cary Eclipse Fluorescence spectrophotometer. Quartz cells with four polished sides (Starna) were used for fluorescence and UV-vis measurements. Fluorescence lifetime and absolute fluorescence quantum yield were performed on an Edinburgh Instruments FLS920 steady-state spectrometer fitted with an integrating sphere. Experiments were carried out in solution using 1 cm path length quartz cuvettes with four transparent polished faces.

2. CHEMICAL STRUCTURES OF MALEIMIDES AND SYNTHETIC PROTOCOLS

Chemical structures of maleimides

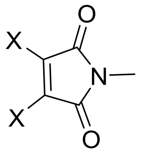

1 (a-b)

a. X=Cl
b. X=Br
c. X=I

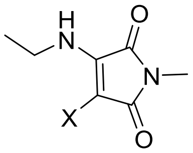

2 (a-c)

a. X=Cl
b. X=Br
c. X=I

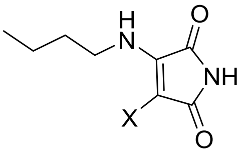

2 (d-e)

d. X=Cl
e. X=Br

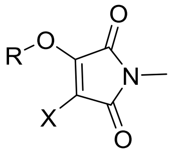

3 (a-f)

a. X=Cl R=CH_2CH_3
b. X=Br R=CH_2CH_3
c. X=Cl R=CH_2Ph
d. X=Br R=CH_2Ph
e. X=Cl R=Ph
f. X=Br R=Ph

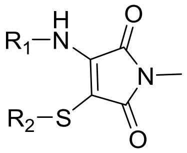

4 (a-g)

a. R_1=CH_2CH_3 R_2=$(CH_2)_3CH_3$
b. R_1=CH_2CH_3 R_2=$(CH_2)_{17}CH_3$
c. R_1=CH_2CH_3 R_2=Ph
d. R_1=CH_2CH_3 R_2=$PhCH_3$
e. R_1=CH_2CH_3 R_2=CH_2Ph
f. R_1=$(CH_2)_3CH_3$ R_2=Ph
g. R_1=Ph R_2=Ph

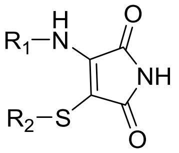

4 (h-j)

h. R_1=$(CH_2)_3CH_3$ R_2=Ph
i. R_1=CH_2CH_3 R_2=$(CH_2)_3CH_3$
j. R_1=$(CH_2)_3CH_3$ R_2=CH_2Ph

Experimental procedure for di-halogen maleimides preparation

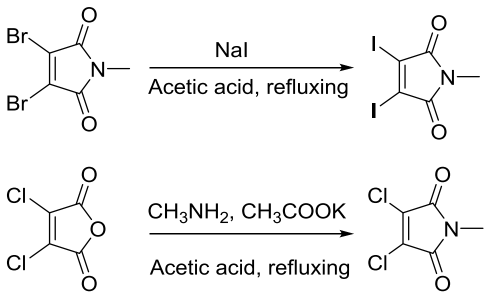

3,4-diiodo-1-methyl-1H-pyrrole-2,5-dione (1c):

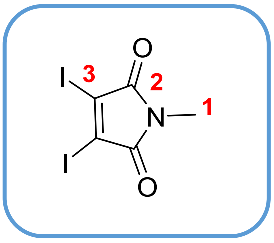

This compound was prepared by the method from the previous literature.[2] 3,4-dibromo-N-methylmaleimide (0.5 g, 1.859 mmol) and sodium iodide (1.115 g, 7.437 mmol) in acetic acid (20 ml) was heated at refluxing temperature for 2 h. Then the solution was added to water. The obtained yellow precipitate was filtered, washing with water and drying at 60 °C to obtain the 3,4-diiodo-1-methyl-1H-pyrrole-2,5-dione (0.527 g, 78.3%). Matches literature data.[2]

TLC conditions: R_f = 0.53 (petroleum ether: ethyl acetate= 4:1), Yield =78.3%. ^{1}H NMR (400 MHz, $CDCl_3$, ppm) δ= 3.15 (1H, s, H1).^{13}C NMR (101 MHz, $CDCl_3$, ppm) δ=166.2 (C2), 117.1 (C3), 26.0 (C1). HR-MS (MaXis) m/z found 358.8141 $(M+Na)^+$, calculated 385.8145; FTIR (neat) ν_{max} / cm^{-1} 1695 and 1560 (C=O of maleimide), 1426 and 1361 (C=C of maleimide).

3,4-dichloro-1-methyl-1H-pyrrole-2,5-dione (1a):

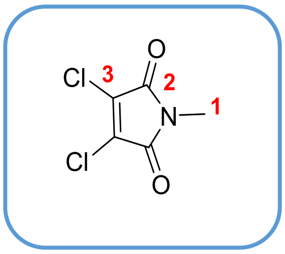

This compound was prepared by the method from the previous literature.[3] 3,4-dichloromaleic anhydride (1 g, 5.98 mmol), methylamine hydrochloride (0.605 g, 8.97 mmol), and potassium acetate (1.24 g, 8.97 mmol) were added to a solution of acetic acid (10 ml), and stirred for 4 hrs under reflux. The reaction become yellow, and was cooled to 25-30 °C, and poured slowly onto chilled sodium bicarbonate solution (1 M, 50 ml) until no effervesce was observed, followed by stirring for 1 hour. The solids were filtered, washed with *n*-hexane (2 x 30 ml) and dried under vacuum to obtain 3,4-dichloro-1-methyl-1H-pyrrole-2,5-dione (0.732 g, 68%). Matches literature data.[3]

TLC conditions: R_f = 0.61 (petroleum ether: ethyl acetate= 4:1), Yield =68.0%. ^{1}H NMR (400 MHz, $CDCl_3$) δ= 3.12 (1H, s, H1); ^{13}C NMR (101 MHz, $CDCl_3$, ppm) δ= 162.8 (C2), 133.51 (C3), 25.1 (C1); FTIR (neat) ν_{max} / cm^{-1} 1708 and 1617 (C=O of maleimide), 1436 and 1381 (C=C of maleimide).

General procedure of aminomaleimides synthesis

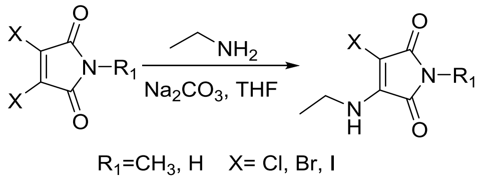

Reactions were performed according to the protocol established by a previous paper.[4] All reactions were performed in THF (20 mL) at room temperature with 2,3-dibromomaleimide/ 2,3-dibromomethylmaleimide (1 eq.), sodium carbonate (2.5 eq.) and a small excess of amine (1.05-1.1 eq.). Consumption of 2,3-dibromomaleimide was monitored by TLC, and was complete within 30 min to 2 h. The solvent was then evaporated under reduced pressure and the residue was taken up with 150 mL of CH_2Cl_2. The resultant mixture was washed with water (2 × 150 mL), dried with sodium sulfate and purified *via* column chromatography on silica gel with petroleum ether/ethyl acetate.

3-chloro-4-(ethylamino)-1-methyl-1H-pyrrole-2,5-dione (2a):

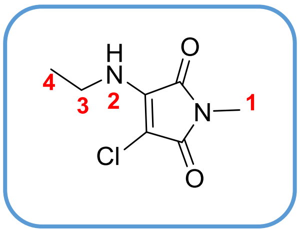

TLC conditions: R_f = 0.60 (petroleum ether: ethyl acetate= 2:1), Yield =60.5%. ^{1}H NMR (400 MHz, $CDCl_3$, ppm) δ=5.30 (1H, s, H2), 3.76 – 3.58 (2H, m, H3), 3.00 (3H, s, H1), 1.30 (3H, t, $^3J_{H-H}$ = 7.2 Hz, H4); ^{13}C NMR (101 MHz, $CDCl_3$, ppm) δ=38.1 (C3), 24.2 (C1), 16.1 (C4); HR-MS (MaXis) m/z found 211.0247 $(M+Na)^+$, calculated 211.0245; FTIR (neat) ν_{max} / cm^{-1} 3344 (H-N of amine), 1694 and 1629 (C=O of maleimide), 1436 and 1372 (C=C of maleimide).

3-bromo-4-(ethylamino)-1-methyl-1H-pyrrole-2,5-dione (2b):

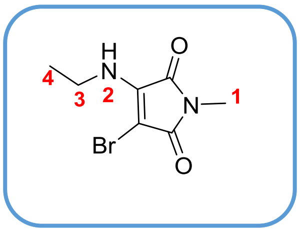

TLC conditions: R_f = 0.60 (petroleum ether: ethyl acetate= 2:1) Yield =54.3%. ^{1}H NMR (400 MHz, $CDCl_3$, ppm) δ=5.35 (1H, s, H2), 3.78 – 3.63 (2H, m, H3), 3.02 (3H, s, H1), 1.29 (3H, t, $^3J_{H-H}$ = 7.2 Hz, H4); ^{13}C NMR (101 MHz, $CDCl_3$, ppm) δ=38.0 (C3), 24.4 (C1), 15.8 (C4); HR-MS (MaXis) m/z found 256.9720

(M+Na) $^+$, calculated 256.9719; FTIR (neat) ν_{max} / cm^{-1} 3332 (H-N of amine), 1707 and 1655 (C=O of maleimide), 1449 and 1372 (C=C of maleimide).

3- iodo-4-(ethylamino)- 1-methyl-1H-pyrrole-2,5-dione (2c):

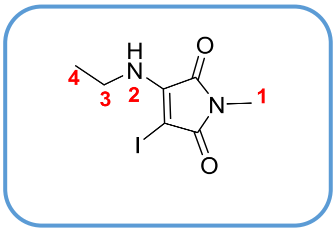

TLC conditions: R_f = 0.60 (petroleum ether: ethyl acetate= 2:1); Yield = 32.1%; ^{1}H NMR (400 MHz, $CDCl_3$, ppm) δ=5.35 (1H, s, H2), 3.78 – 3.63 (2H, m, H3), 3.02 (3H, s, H1), 1.29 (3H, t, $^3J_{H-H}$ 7 Hz, H4). ^{13}C NMR (101 MHz, $CDCl_3$, ppm) δ=38.0 (C3), 24.4 (C1), 15.8 (C4); HR-MS (MaXis) m/z found 302.9604 (M+Na) $^+$, calculated 302.9601. FTIR (neat) ν_{max} / cm^{-1} 3332 (H-N of amine), 1604 (C=O of maleimide), 1449 (C=C of maleimide).

3-(butylamino)-4-chloro-1-methyl-1H-pyrrole-2,5-dione (2d):

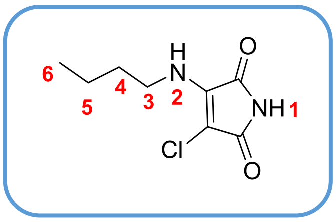

TLC conditions: R_f = 0.50 (petroleum ether: ethyl acetate= 2:1), Yield = 58.7%. ^{1}H NMR (400 MHz, $CDCl_3$, ppm) δ=7.08 (1H, s, H1), 5.31 (1H, s, H2), 3.62 (2H, td, $^3J_{H-H}$ = 14, 7 Hz, H3), 1.74 – 1.53 (2H, m, $^3J_{H-H}$ = 7 Hz, H4), 1.48 – 1.34 (2H, sex, $^3J_{H-H}$ = 7 Hz, H5), 0.97 (3H, t, $^3J_{H-H}$ = 7 Hz, H6). ^{13}C NMR (101 MHz, $CDCl_3$, ppm) δ=42.8 (C3), 32.8 (C4), 19.6 (C5), 13.6 (C6); HR-MS (MaXis) m/z found 225.0404 (M+Na) $^+$, calculated 225.0401; FTIR (neat) ν_{max} / cm^{-1} 3319 (H-N of amine), 3138 (H-N of maleimide) 1772 and 1707 (C=O of maleimide), 1404 and 1359 (C=C of maleimide).

3-bromo-4-(butylamino)-1H-pyrrole-2,5-dione (2e):

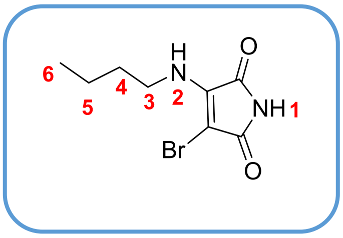

TLC conditions: R_f = 0.50 (petroleum ether: ethyl acetate= 2:1), Yield = 69.2%; ^{1}H NMR (400 MHz, $CDCl_3$, ppm) δ=7.17 (1H, s, H1), 5.41 (1H, s, H2), 3.65 (2H, dd, J = 7 Hz, H3), 1.70 – 1.58 (2H, quin, $^3J_{H-H}$ = 7 Hz, H4), 1.42 (2H, sex, $^3J_{H-H}$ =7 Hz, H5), 0.97 (3H, t, $^3J_{H-H}$ = 7 Hz, H6). ^{13}C NMR (101 MHz, $CDCl_3$) δ=42.9 (C3), 32.7 (C4), 19.5 (C5), 13.7 (C6); HR-MS (MaXis) m/z found 268.9898 (M+Na) $^+$, calculated 268.9896. FTIR (neat) ν_{max} / cm^{-1} 3344 (H-N of amine), 3138 (H-N of maleimide) 1617 and 1707 (C=O of maleimide), 1462 and 1333 (C=C of maleimide);

General procedure for alkoxymaleimides synthesis

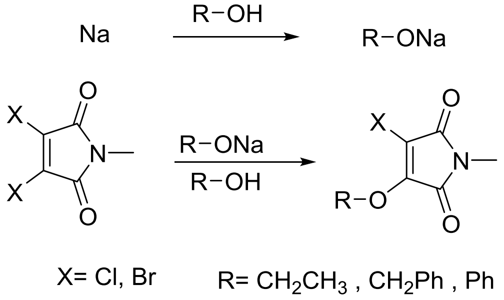

Reactions were performed according to the protocol established by a previous paper.[5] Sodium alkoxide solution was freshly prepared by the addition of sodium (2 mmol, 1 equiv) to the desired alcohol. This solution was then added dropwise to a cooled solution of the maleimide (2 mmol, 1 equiv) in the same alcohol (10 mL). This was kept at 0 °C in an ice bath. Consumption of 2, 3-dibromo/chloro maleimide was monitored by TLC, and was complete within 30 min to 1 h. Then the reaction mixture was concentrated in vacuo and separated between ethyl acetate and aqueous ammonium chloride solution. The aqueous layer was washed with ethyl acetate (50 mL×3), then the combined organic extracts washed with water (50 mL×3), brine, and dried with Na_2SO_4. Concentration *in vacuo*, followed by purification *via* column chromatography on silica gel with petroleum ether/ethyl acetate, afforded the products.

3-chloro-4-ethoxy-1-methyl-1H-pyrrole-2,5-dione (3a):

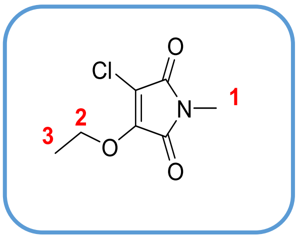

TLC conditions: R_f = 0.38 (petroleum ether: ethyl acetate= 4:1). Yield = 55.1%. ^{1}H NMR (400 MHz, $CDCl_3$, ppm) δ=4.72 (2H, q, $^3J_{H-H}$ = 7 Hz, H2), 3.05 (3H, s, H1), 1.49 (3H, t, $^3J_{H-H}$ = 7 Hz, H3). ^{13}C NMR (101 MHz, $CDCl_3$, ppm) δ=68.8 (C2), 23.9 (C1), 15.1 (C3). HR-MS (MaXis) m/z found 212.0084 $(M+Na)^+$, calculated 212.0085; FTIR (neat) ν_{max} / cm^{-1} 1707 and 1655 (C=O of maleimide), 1436 and 1384 (C=C of maleimide), 1255 (C-O of alcohol).

3-bromo-4-ethoxy-1-methyl-1H-pyrrole-2, 5-dione (3b):

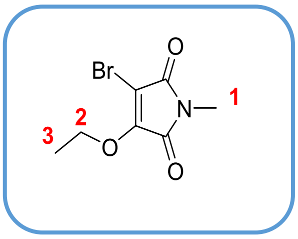

TLC conditions: R_f = 0.38 (petroleum ether: ethyl acetate= 4:1). Yield = 42.5%.; ^{1}H NMR (400 MHz, $CDCl_3$, ppm) δ=4.70 (2H, q, $^3J_{H-H}$ = 7 Hz, H2), 3.03 (3H, s, H1), 1.47 (3H, t, $^3J_{H-H}$ = 7 Hz, H3); ^{13}C NMR (101 MHz, $CDCl_3$) δ 68.7 (C2), 24.1 (C1), 15.4

(C3); FTIR (neat) ν_{max} / cm^{-1} 1720 and 1640 (C=O of maleimide), 1436 and 1382 (C=C of maleimide), 1268 (C-O of alcohol).

3-(benzyloxy)-4-chloro-1-methyl-1H-pyrrole-2,5-dione (3c):

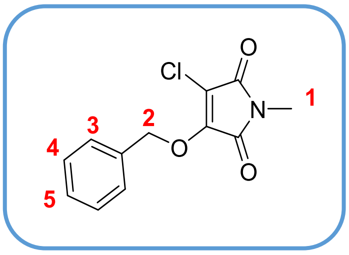

TLC conditions: R_f = 0.41 (petroleum ether: ethyl acetate= 4:1). Yield =43.7%; ^{1}H NMR (400 MHz, DMSO, ppm) δ=7.60 – 7.39 (5H, m, H3), 5.71 (2H, s, H2), 2.94 (3H, s, H3); ^{13}C NMR (101 MHz, DMSO, ppm) δ=131.24 – 125.94 (m, C3), 73.83 (C2), 24.42 (C1); HR-MS (MaXis) *m/z* found 274.0243, calc. 274.0241 ([M+Na] $^{+,}$100%); FTIR (neat) ν_{max} / cm^{-1} 1720 and 1642 (C=O of maleimide), 1449 and 1397 (C=C of maleimide), 1255 (C-O of alcohol), 752 and 701 (C-H of aromatic).

3-(benzyloxy)-4-bromo-1-methyl-1H-pyrrole-2,5-dione (3d):

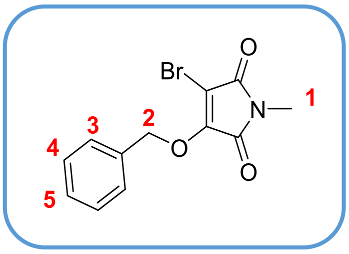

TLC conditions: R_f = 0.41 (petroleum ether: ethyl acetate= 4:1). Yield = 32.1%; ^{1}H NMR (400 MHz, DMSO, ppm) δ=7.55 – 7.35 (5H, m, H3), 5.69 (2H, s, H2), 2.90 (3H, s, H3); ^{13}C NMR (101 MHz, DMSO, ppm) δ=130.4 – 126.2 (m, C3), 73.8 (C2), 24.5 (C1); HR-MS (MaXis) *m/z* found 317.9731, calc. 317.9736 ([M+Na] $^{+,}$100%); FTIR (neat) ν_{max} / cm^{-1} 1707 and 1642 (C=O of maleimide), 1423 and 1382 (C=C of maleimide), 1255 (C-O of alcohol), 740 and 688 (C-H of aromatic);

3-chloro-1-methyl-4-phenoxy-1H-pyrrole-2,5-dione (3e):

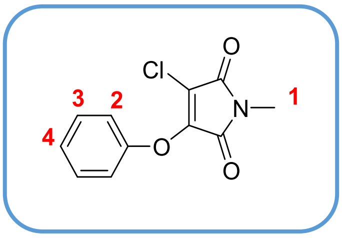

TLC conditions: R_f = 0.41 (petroleum ether: ethyl acetate= 4:1); Yield =55.1%; ^{1}H NMR (400 MHz, MeOD, ppm) δ=7.43 (2H, t, J = 8 Hz, H2), 7.28 (2H, t, J = 7 Hz, H3), 7.23 (1H, d, J = 8 Hz,), 3.03 (3H, s, H1). ^{13}C NMR (101 MHz, MeOD, ppm) δ=129.43 (C2), 125.47 (C3), 118.70 (C4), 22.87 (C1); HR-MS (MaXis) m/z found 260.0085 (M+Na) $^+$, calculated 260.0085; FTIR (neat) ν_{max} / cm^{-1} 1720 (C=O of maleimide), 1433 and 1392 (C=C of maleimide), 1270 (C-O of alcohol), 774 and 695 (C-H of aromatic).

3-bromo-1-methyl-4-phenoxy-1H-pyrrole-2,5-dione (3f):

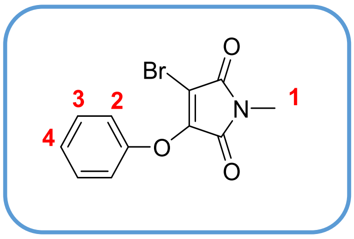

TLC conditions: R_f = 0.41 (petroleum ether: ethyl acetate= 4:1); Yield =51.0%. ^{1}H NMR (400 MHz, DMSO, ppm) δ=7.43 (2H, t, J = 7.5 Hz, H2), 7.28 (3H, dd, J = 14, 7 Hz, H2,3), 2.93 (3H, s, H1); ^{13}C NMR (101 MHz, DMSO, ppm) δ=130.2 (C2), 125.9 (C3), 119.1 (C4), 24.7 (C1). HR-MS (MaXis) m/z found 303.9586 (M+Na)$^+$, calculated 303.9580; FTIR (neat) ν_{max} / cm^{-1} 1720 (C=O of maleimide), 1436 and 1359 (C=C of maleimide), 1165 (C-O of alcohol), 830 and 714 (C-H of aromatic).

General procedure for thioaminomaleimide synthesis:

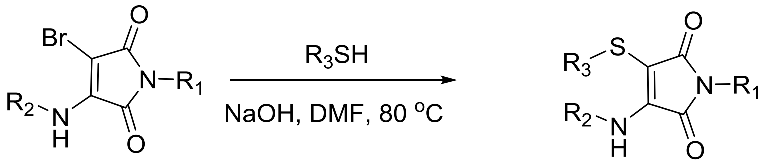

Aminobromomaleimide (2 mmol, 1 equiv.) was dissolved in DMF (30 mL) followed by addition of NaOH (3 mmol, 1.5 equiv). The mixture was stirred for 5 minutes and then thiol (2 mmpl, 1 equiv.) was added dropwise. Upon complete addition, the solution was allowed to warm to 80 °C and left to stir for 20 hours. Then the reaction mixture was concentrated in vacuo and dissolved in ethyl acetate. The organic solution washed with water, brine and dried over anhydrous Na_2SO_4. The solution was filtered and concentrated *in vacuo*, and the crude product was purified by column chromatography (petroleum ether: ethyl acetate = 10:1-3:1) to yield the yellow product.

3-(butylthio)-4-(ethylamino)-1-methyl-1H-pyrrole-2,5-dione (4a):

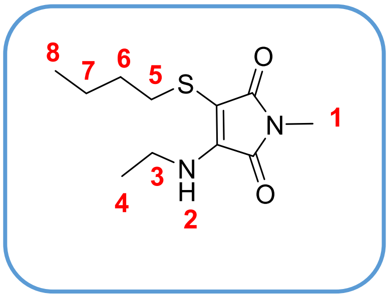

TLC conditions: R_f = 0.63 (petroleum ether: ethyl acetate= 2:1); Yield =36.8%; ^{1}H NMR (400 MHz, $CDCl_3$, ppm) δ=5.54 (1H, s, H2), 3.88 – 3.79 (2H, m, H3), 3.01 (3H, s, H1), 2.71 – 2.65 (2H, t, $^3J_{H-H}$ = 7 Hz, H5), 1.61 – 1.51 (2H, quin, $^3J_{H-H}$ = 7 Hz,H6), 1.41 (2H, sex, $^3J_{H-H}$ = 7 Hz, H7), 1.30 (3H, t, $^3J_{H-H}$ = 7 Hz, H4), 0.91 (3H, t, $^3J_{H-H}$ = 7 Hz, H8); ^{13}C NMR (101 MHz, $CDCl_3$, ppm) δ=38.4 (C3), 35.7 (C5), 31.9 (C6), 24.1 (C1), 21.8 (C7), 15.4 (C4), 13.6 (C8); HR-MS (MaXis) *m/z* found 265.0983, calc. 265.0981 ([M+Na]$^{+,}$ 100%); FTIR (neat) ν_{max} / cm^{-1} 3307 (H-N of amine), 1708 and 1617 (C=O of maleimide), 1436 and 1371 (C=C of maleimide).

3-(ethylamino)-1-methyl-4-(octadecanethio)-1H-pyrrole-2,5-dione (4b):

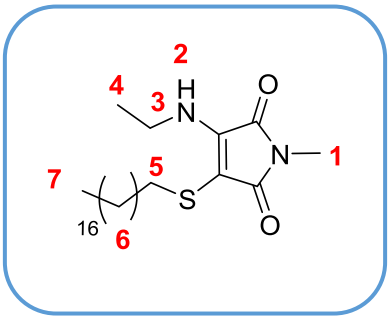

TLC conditions: R_f = 0.39 (petroleum ether: ethyl acetate= 2:1); Yield =18.2 %; ^{1}H NMR (400 MHz, $CDCl_3$, ppm) δ=5.53 (1H, s, H2), 3.82 (2H, p, $^3J_{H-H}$ = 7 Hz, H3), 3.00 (3H, s, H1), 2.69 – 2.64 (2H, m, H5), 1.32 – 1.19 (34H, m, H6+H4), 0.88 (3H, t, J = 7 Hz, H7); ^{13}C NMR (1 MHz, $CDCl_3$, ppm) δ=38.5 (C3), 36.2 (C5), 31.9- 29.8 (C6), 24.2 (C4), 22.9 (C6), 15.3 (C7), 14.0 (C1); HR-MS (MaXis) *m/z* found 461.3171, calc. 461.3172 ([M+Na] $^{+,}$ 100%); FTIR (neat) ν_{max} / cm^{-1} 3320 (H-N of amine), 1700 and 1621 (C=O of maleimide), 1463 and 1391 (C=C of maleimide).

3-(ethylamino)-1-methyl-4-(phenylthio)-1H-pyrrole-2,5-dione (4c):

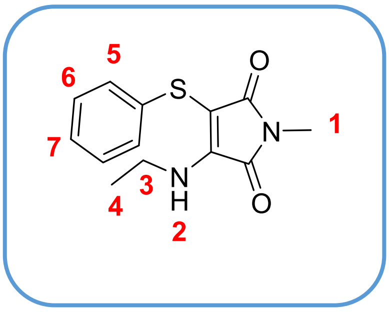

TLC conditions: R_f = 0.42 (petroleum ether: ethyl acetate= 2:1); Yield =50.9%; ^{1}H NMR (400 MHz, CD_3CN, ppm) δ= 7.30 (2H, dd, $^3J_{H-H}$ = 10, 4 Hz, H5), 7.23 (2H, dd, $^3J_{H-H}$ = 5, 3 Hz, H6), 7.19 – 7.14 (1H, m, H7), 3.70 (2H, p, $^3J_{H-H}$ = 7 Hz, H3), 2.96 (3H, s, H1), 1.16 (t, $^3J_{H-H}$ = 3H, 7 Hz, H4). ^{13}C NMR (101 MHz, CD_3CN, ppm) δ=129.6 (C5), 126.0 (C6), 125.8 (C7), 38.3 (C3), 24.1 (C1), 15.4 (C4). HR-MS (MaXis) *m/z* found 285.0667, calc. 285.0668 ([M+Na] $^{+,}$ 100%); FTIR (neat) ν_{max} / cm^{-1} 3313 (H-N of amine), 1696 and 1614 (C=O of maleimide), 1437 and 1390 (C=C of maleimide), 792 and 683 (C-H of aromatic).

3-(ethylamino)-1-methyl-4-(o-tolylthio)-1H-pyrrole-2,5-dione (4d):

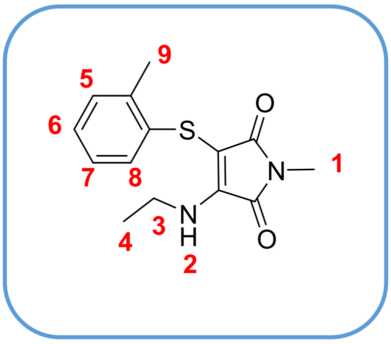

TLC conditions: R_f = 0.47 (petroleum ether: ethyl acetate= 2:1); Yield =55.6%; ^{1}H NMR (400 MHz, CD_3CN, ppm) δ=7.17 (1H, t, $^3J_{H-H}$ = 8 Hz,H5), 7.12 (1H , d, $^3J_{H-H}$ = 7.5 Hz, H6), 7.06 (2H, t, $^3J_{H-H}$ = 6 Hz,H7,H8), 3.65 (2H, p, $^3J_{H-H}$ = 7 Hz, H3), 2.97 (3H, s,H1), 2.38 (3H, s, H9), 1.13 (1H, t, $^3J_{H-H}$ = 7 Hz,H4);^{13}C NMR (101 MHz, CD_3CN, ppm) δ =130.6 (C5), 127.1 (C7), 125.4 (C6), 125.2 (C8), 38.3 (C3), 24.1 (C1), 19.3 (C9), 15.4 (C4); HR-MS (MaXis) *m/z* found 299.0827, calc. 299.0825 ([M+Na] $^{+,}$100%); FTIR (neat) ν_{max} / cm^{-1} 3327 (H-N of amine),

1703 and 1602 (C=O of maleimide), 1436 and 1383 (C=C of maleimide), 797 and 744 (C-H of aromatic).

3-(benzylthio)-4-(ethylamino)-1-methyl-1H-pyrrole-2,5-dione (4e):

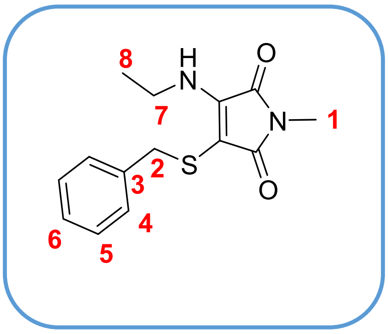

TLC conditions: R_f = 0.39 (petroleum ether: ethyl acetate= 2:1); Yield =11.9%; ^{1}H NMR (400 MHz, DMSO) δ 7.23 (5H, ddd, J = 26, 16, 7 Hz, H 4-6), 3.78 (2H, s, H3), 3.27 (2H, dd, J = 13, 6 Hz, H7), 2.88 (3H, s, H1), 0.90 (3H, t, J = 7 Hz, H8); ^{13}C NMR (101 MHz, DMSO, ppm) δ 129.2 (C4), 128.7, 127.3 (C5, C6), 37.8 (C7), 24.1 (C1), 15.0 (C8); HR-MS (MaXis) *m/z* found 299.0825, calc. 299.0825 ([M+Na]$^{+,}$ 100%); FTIR (neat) ν_{max} / cm^{-1} 3306 (H-N of amine), 1707 and 1604 (C=O of maleimide), 1345 (C=C of maleimide, 757 and 641 (C-H of aromatic).

3-(butylamino)-1-methyl-4-(phenylthio)-1H-pyrrole-2,5-dione (4f):

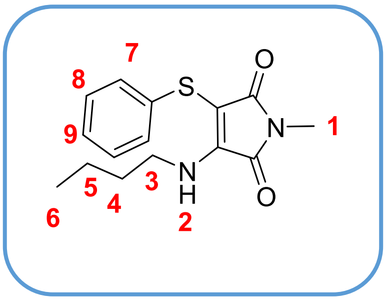

TLC conditions: R_f = 0.23 (petroleum ether: ethyl acetate= 2:1). Yield =42.3%. ^{1}H NMR (400 MHz, DMSO, ppm) δ=7.28 (2H, t, *J* = 7 Hz, H7), 7.13 (3H, dd, *J* = 14, 7 Hz, H8-9), 3.52 (2H, d, *J* = 6 Hz, H3), 2.91 (3H, s, H1), 1.52 – 1.33 (2H, m, H4), 1.17 (2H, dd, *J* = 14, 7 Hz, H5), 0.71 (3H, t, *J* = 7 Hz, H6); ^{13}C NMR (101 MHz, DMSO) δ 129.5 (C7), 125.4 (C8), 125.3 (C9), 42.6 (C3), 32.2 (C4), 24.5 (C1), 19.6 (C5), 13.9 (C6); HR-MS (MaXis) *m/z* found 289.1015, calc. 289.1016 ([M+Na]$^{+,}$ 100%); FTIR (neat) ν_{max} / cm^{-1} 3332 (H-N of amine), 1681 and 1604 (C=O of maleimide), 1436 and 1384 (C=C of maleimide), 740 and 688 (C-H of aromatic).

1-methyl-3-(phenylamino)-4-(phenylthio)-1H-pyrrole-2,5-dione (4g):

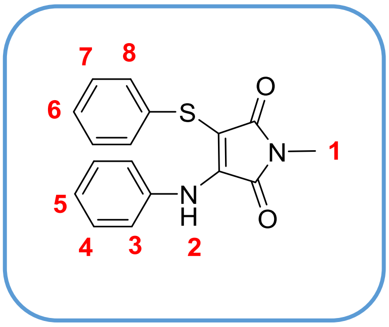

TLC conditions: R_f = 0.44 (petroleum ether: ethyl acetate= 2:1); Yield =44.2%; ^{1}H NMR (400 MHz, DMSO, ppm) δ= 10.10 (1H, s, H2), 7.18 – 6.99 (8H, m, H4-8), 6.91 (H2, d, J = 7 Hz, H3), 2.97 (3H, s, H1); ^{13}C NMR (101 MHz, DMSO, ppm) δ 129.1 (C5), 128.0 (C8), 126.3 (C4), 125.8 (C7), 125.5 (C5), 124.8 (C6), 24.6

(C1); HR-MS (MaXis) *m/z* found 333.0678, calc. 333.0668 ($[M+Na]^{+}$, 100%); FTIR (neat) ν_{max} / cm^{-1} 3281 (H-N of amine), 1694 and 1604 (C=O of maleimide), 1449 (C=C of maleimide), 740 and 688 (C-H of aromatic).

3-(butylamino)-4-(phenylthio)-1H-pyrrole-2,5-dione (4h):

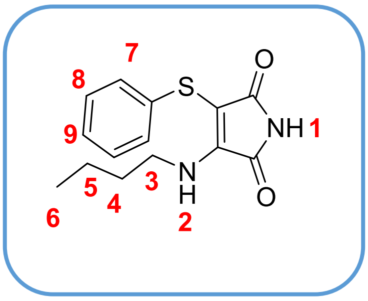

TLC conditions: R_f = 0.23 (petroleum ether: ethyl acetate= 2:1); Yield = 50.1%; ^{1}H NMR (400 MHz, DMSO, ppm) δ=10.75 (1H, s, H1), 8.32 (1H, s, H2), 7.29 (2H, t, $^{3}J_{H-H}$ = 7 Hz, H7), 7.18 – 7.06 (3H, m, H8, H9), 3.51 (2H, d, $^{3}J_{H-H}$ = 7 Hz, H3), 1.49 – 1.33 (2H, m, H4), 1.22 – 1.08 (2H, m, H5), 0.72 (3H, t, $^{3}J_{H-H}$ = 7 Hz, H6); ^{13}C NMR (101 MHz, DMSO, ppm) δ 129.5 (C7), 125.3 (C8, C9), 42.4 (C3), 32.2 (C4), 19.6 (C5), 13.9 (C6); HR-MS (MaXis) *m/z* found 299.0872, calc. 299.0825 ($[M+Na]^{+}$, 100%); FTIR (neat) ν_{max} / cm^{-1} 3216 (H-N of amine), 1720 and 1617 (C=O of maleimide), 1345 (C=C of maleimide), 740 and 688 (C-H of aromatic).

3-(butylthio)-4-(ethylamino)-1H-pyrrole-2,5-dione (4i):

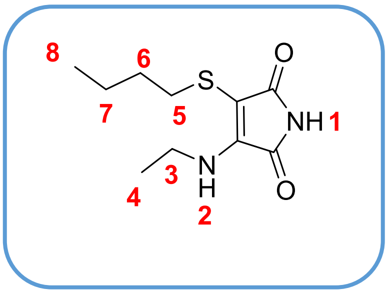

TLC conditions: R_f = 0.39 (petroleum ether: ethyl acetate= 2:1); Yield =18.9%; ^{1}H NMR (400 MHz, $CDCl_3$, ppm) δ=7.2 (1H, s, H1), 5.5 (1H, s, H2), 3.8 (2H, p, $^{3}J_{H-H}$ = 7 Hz, H3), 2.7 – 2.5 (2H, m, H5), 1.6 – 1.5 (2H, m, H6), 1.4 – 1.3 (2H, m, H7), 1.2 (3H, $^{3}J_{H-H}$ = 7 Hz, H4), 0.8 (3H, $^{3}J_{H-H}$ = 7 Hz, H8). ^{13}C NMR (101 MHz, $CDCl_3$, ppm) δ 171.1, 166.3 (C=O), 38.4 (C3), 35.6 (C5), 31.9 (C6), 21.9 (C7), 15.4(C4), 13.6 (C8); HR-MS (MaXis) *m/z* found 229.1007, calc. 229.1005 ($[M+H]^{+}$, 100%) FTIR (neat) ν_{max} / cm^{-1} 3319 (H-N of amine), 3164 (H-N of maleimide), 1707 and 1617 (C=O of maleimide), 1359 (C=C of maleimide).

3-(benzylamino)-4-(butylthio)-1H-pyrrole-2,5-dione (4j):

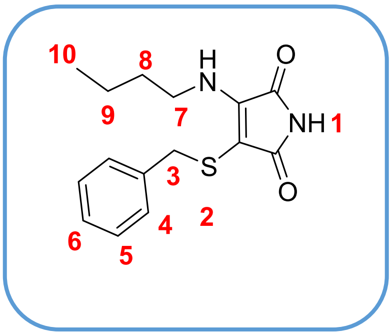

TLC conditions: R_f = 0.42 (petroleum ether: ethyl acetate= 2:1). Yield =26.4%. ^{1}H NMR (400 MHz, MeOD, ppm) δ=7.23 (5H, ddd, $^{3}J_{H-H}$ = 25, 16, 7; H 4, 5, 6), 3.8 (2H, s, H3), 3.25 (2H, t, $^{3}J_{H-H}$ = 7 Hz, H7), 1.49 – 1.19 (4H, m, H8, 9), 0.9 (3H, t, $^{3}J_{H-H}$ = 7 Hz, H10); ^{13}C NMR (101 MHz, MeOD, ppm) δ 128. 7, 127.8, 126.7 (C6, 5, 4), 42.0 (C7), 39.3 (C8), 31.2 (C9), 19.4 (C10); HR-MS (MaXis) *m/z* found 313.0981, calc. 313.0981 ([M+Na] $^{+,}$100%); FTIR (neat) ν_{max} / cm^{-1} 3315 (H-N of amine), 1711 and 1620 (C=O of maleimide), 1359 (C=C of maleimide, 765 and 705 (C-H of aromatic).

3. FLUOROPHORE CHARACTERIZATION

Table S1 Structures of substituted maleimides and associated fluorescence properties

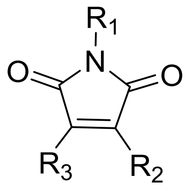

Sample	R1	R2	R3	Solvent	Φ_f^a %	ε_{max} $M^{-1}cm^{-1}$	λ^b_{abs} nm	λ^b_{ex} nm	λ^b_{em} nm	$\Delta\lambda^c$ nm
2a	CH_3	$NHCH_2CH_3$	Cl	Diethyl Ether	37	3669	239, 369	365	475	236,106
				Cyclohexane	56	3198	238, 365	364	482	244,117
				Methanol	1	3355	239, 379	380	550	311, 171
				Water	<0.1	2442	241, 398	397	580	339, 182
2b	CH_3	$NHCH_2CH_3$	Br	Diethyl Ether	30	3733	239, 370	371	474	235, 104
				Cyclohexane	37	3496	239, 366	364	472	233, 106
				Methanol	1	4811	241, 384	379	548	307, 164
				Water	<0.1	3645	241, 400	397	583	342, 183
2c	CH_3	$NHCH_2CH_3$	I	Diethyl Ether	8	4419	237, 370	368	487	250, 117
				Cyclohexane	11	3483	239, 372	363	472	233, 100
				Methanol	1	3405	238, 388	383	560	322, 172
				Water	<0.1	2925	238, 404	396	580	342, 176
2d	H	$NH(CH_2)_3CH_3$	Cl	Diethyl Ether	42	5073	226, 359	355	461	235, 102
				Cyclohexane	65	4220	226, 356	352	442	216, 72
				Methanol	1	4313	229, 374	370	512	283, 142
				Water	<0.1	2867	228, 387	387	550	322, 180
2e	H	$NH(CH_2)_3CH_3$	Br	Diethyl Ether	31	5531	226, 358	358	461	235, 103
				Cyclohexane	47	4489	226, 355	351	441	215, 86
				Methanol	2	4451	229, 374	367	513	284, 139
				Water	<0.1	2768	228, 387	387	547	319, 160
3a	CH_3	OCH_2CH_3	Cl	Diethyl Ether	12	770	230, 335	332	458	228, 123
				Cyclohexane	24	875	237, 330	332	461	224, 131
				Methanol	2	825	236, 333	330	486	250, 153
				Water	<0.1	548	236, 342	341	505	269, 163
3b	CH_3	OCH_2CH_3	Br	Diethyl Ether	10	666	230, 336	332	458	228, 122
				Cyclohexane	24	668	238, 333	332	461	223, 128
				Methanol	2	629	237, 335	332	486	249, 151
				Water	<0.1	432	236, 343	344	501	265, 158
3c	CH_3	OCH_2Ph	Cl	Diethyl Ether	12	1267	232, 335	334	464	232, 129
3d	CH_3	OCH_2Ph	Br	Diethyl Ether	9	907	233, 340	329	465	232, 125
3e	CH_3	OPh	Cl	Diethyl Ether	<0.1	586	238, 333	--	--	--
3f	CH_3	OPh	Br	Diethyl Ether	<0.1	821	243, 325	--	--	--

Sample	R_1	R_2	R_3	Solvent	Φ_f^a %	ε_{max} $M^{-1}cm^{-1}$	λ^b_{abs} nm	λ^b_{ex} nm	λ^b_{em} nm	$\Delta\lambda^c$ nm
4a	CH_3	$NHCH_2CH_3$	$S(CH_2)_3CH_3$	Diethyl Ether	4	1917	240, 379	377	558	318, 165
4b	CH_3	$NHCH_2CH_3$	$S(CH_2)_{17}CH_3$	Diethyl Ether	6	2790	232, 383	377	561	329, 160
4c	CH_3	$NHCH_2CH_3$	SPh	Diethyl Ether	12	1761	230, 370	364	545	315, 160
4d	CH_3	$NHCH_2CH_3$	$SPhCH_3$	Diethyl Ether	12	2595	236, 370	367	547	311, 161
4e	CH_3	$NHCH_2CH_3$	SCH_2Ph	Diethyl Ether	4	2130	238, 381	378	564	326, 164
4f	CH_3	$NH(CH_2)_3CH_3$	SPh	Diethyl Ether	9	3898	246, 368	359	544	298, 160
4g	CH_3	NHPh	SPh	Diethyl Ether	<0.1	4202	230, 398	--	--	--
4h	H	$NH(CH_2)_3CH_3$	SPh	Diethyl Ether	23	2685	230, 355	355	526	296, 157
				Cyclohexane	37	4743	241, 355	347	513	272, 147
				Methanol	<0.1	4269	241, 370	364	593	352, 223
				Water	--	--	--	--	--	--
4i	H	$NHCH_2CH_3$	$S(CH_2)_3CH_3$	Diethyl Ether	13	4363	227, 378	369	535	308, 147
4j	H	$NHCH_2CH_3$	SCH_2Ph	Diethyl Ether	17	2276	224, 370	367	532	308, 152

[a] Relative fluorescent quantum yield measured using quinine sulfate as reference (Φ_f = 59%, in 0.105 M $HClO_4$).[6]

[b] The absorption, excitation and emission wavelength were recorded at 10 μM in diethyl ether, slit width ex 5 nm, em 5 nm.

[c] Stokes shift in diethyl ether solution (10 μM).

4. UV-VIS SPECTRUM OF MALEIMIDES

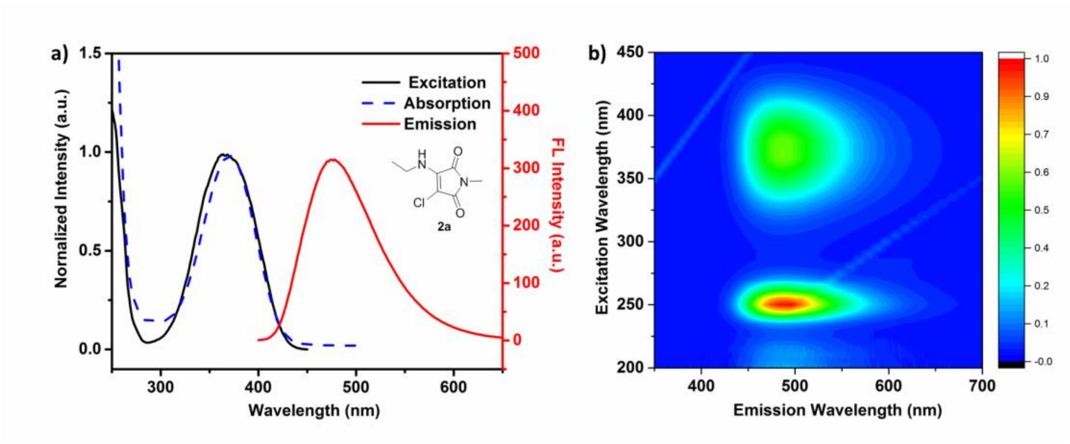

Figure S1. a) Excitation, emission and absorption spectra of **2a** in diethyl ether at 10 µM; b) 2D excitation-emission spectra (with a 5 nm step) of **2a** in diethyl ether at 10 µM. Peaks at $\lambda_{ex} = \lambda_{em}$ are due to Rayleigh scattering from the sample solution.

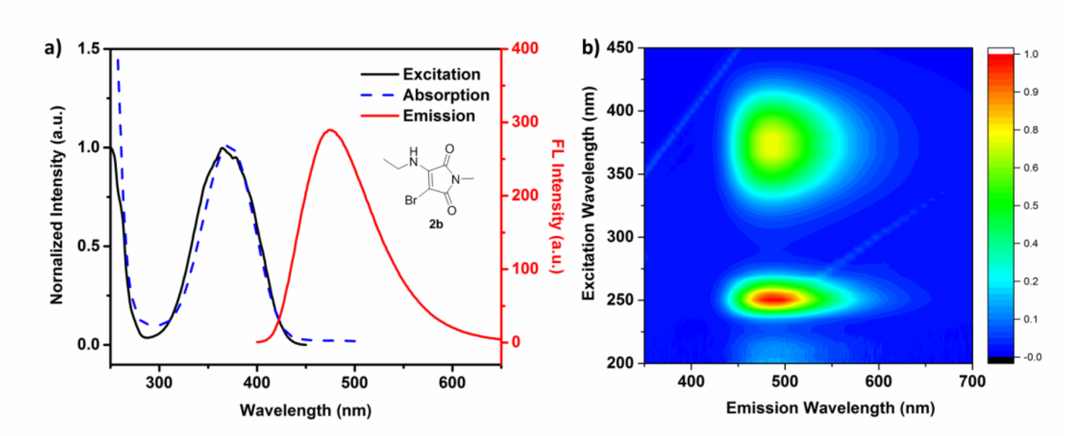

Figure S2. a) Excitation, emission and absorption spectra of **2b** in diethyl ether at 10 µM; b) 2D excitation-emission spectra (with a 5 nm step) of **2b** in diethyl ether at 10 µM. Peaks at $\lambda_{ex} = \lambda_{em}$ are due to Rayleigh scattering from the sample solution.

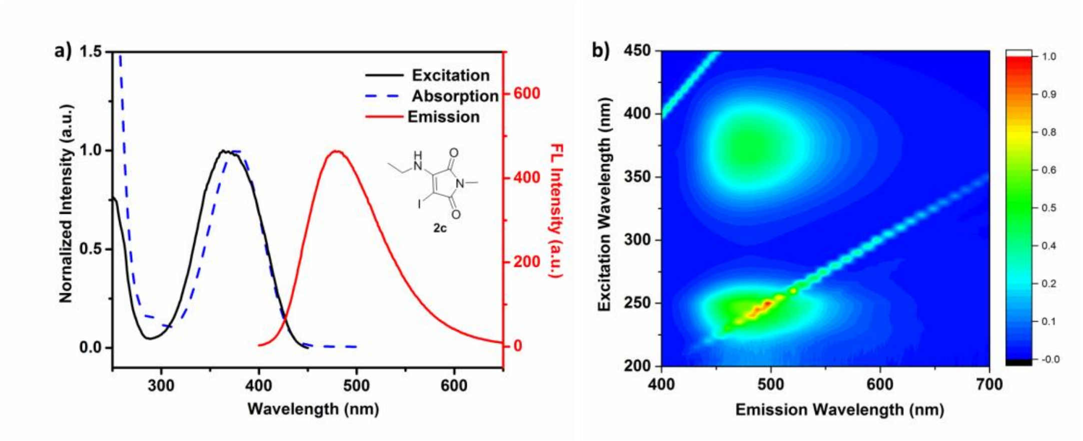

Figure S3. a) Excitation, emission and absorption spectra of **2c** in diethyl ether at 10 µM; b) 2D excitation-emission spectra (with a 5 nm step) of **2c** in diethyl ether at 10 µM. Peaks at $\lambda_{ex} = \lambda_{em}$ are due to Rayleigh scattering from the sample solution.

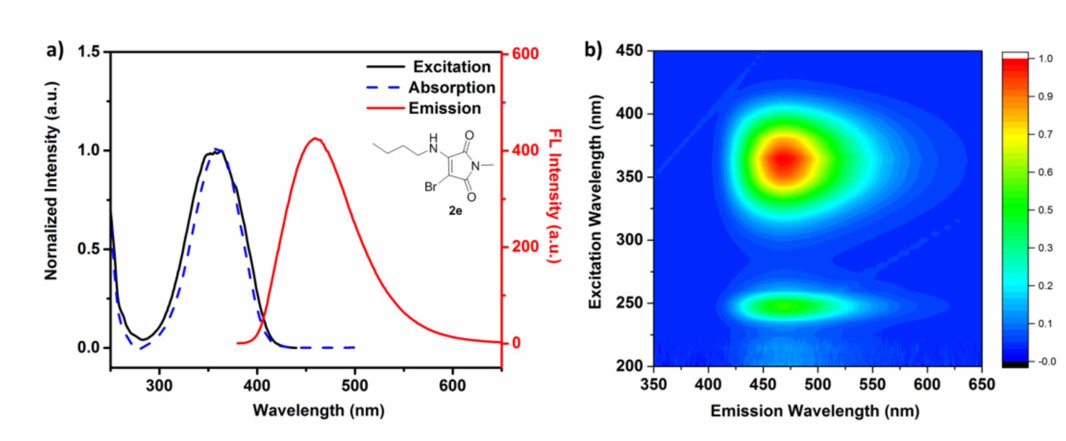

Figure S4. a) Excitation, emission and absorption spectra of **2e** in diethyl ether at 10 μM; b) 2D excitation-emission spectra (with a 5 nm step) of **2e** in diethyl ether at 10 μM. Peaks at λex = λem are due to Rayleigh scattering from the sample solution.

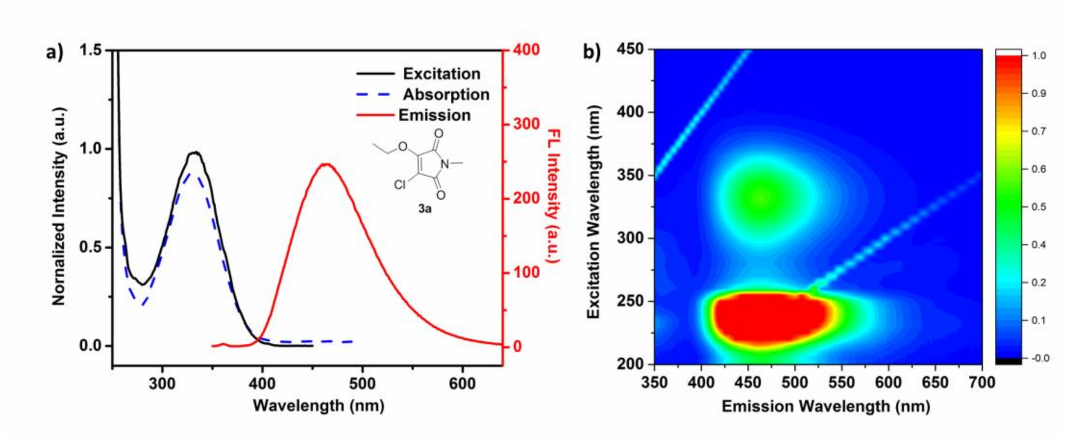

Figure S5. a) Excitation, emission and absorption spectra of **3a** in diethyl ether at 10 μM; b) 2D excitation-emission spectra (with a 5 nm step) of **3a** in diethyl ether at 10 μM. Peaks at λex = λem are due to Rayleigh scattering from the sample solution.

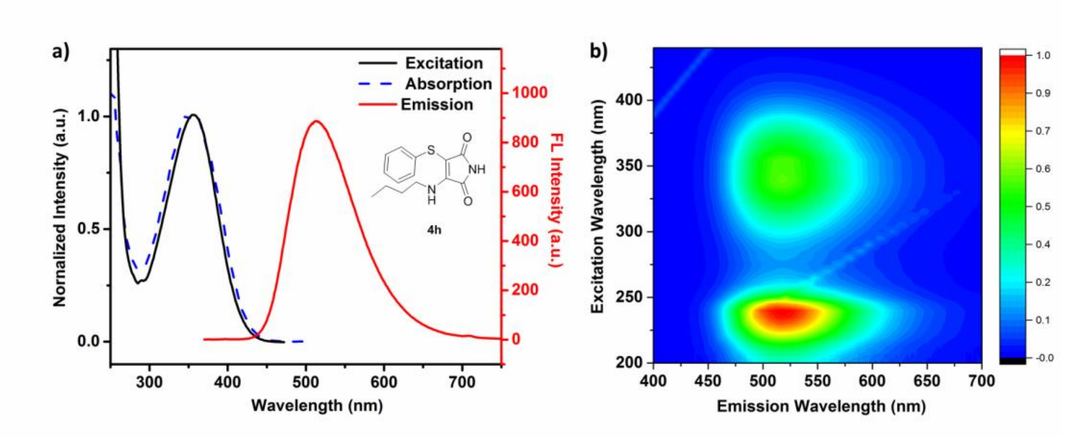

Figure S6. a) Excitation, emission and absorption spectra of **4h** in diethyl ether at 10 μM; b) 2D excitation-emission spectra (with a 5 nm step) of **4h** in diethyl ether at 10 μM. Peaks at λex = λem are due to Rayleigh scattering from the sample solution.

5. SOLVATOCHROMIC DETERMINATION

The emission spectra of each compound in different solvents was recorded using the corresponding absorption peak as the excitation wavelength. (CH= cyclohexane, DE= diethyl ether, THF=tetrahydrofutan, MeOH=Methanol, DMF= Dimethylformamide,)

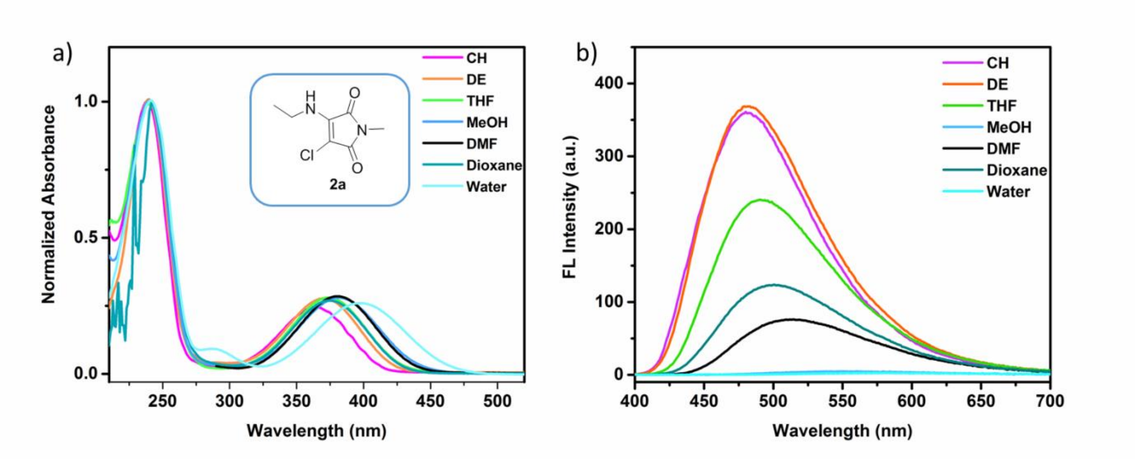

Figure S7. Absorption (a) and emission (b) spectra of **2a** in different solvents at 10 µM.

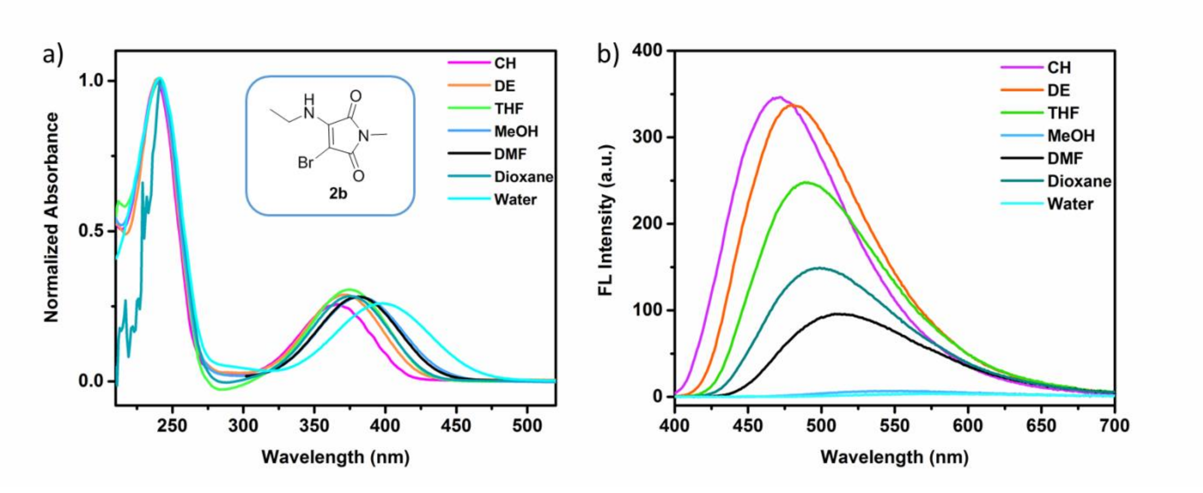

Figure S8. Absorption (a) and emission (b) spectra of **2b** in different solvents at 10 µM.

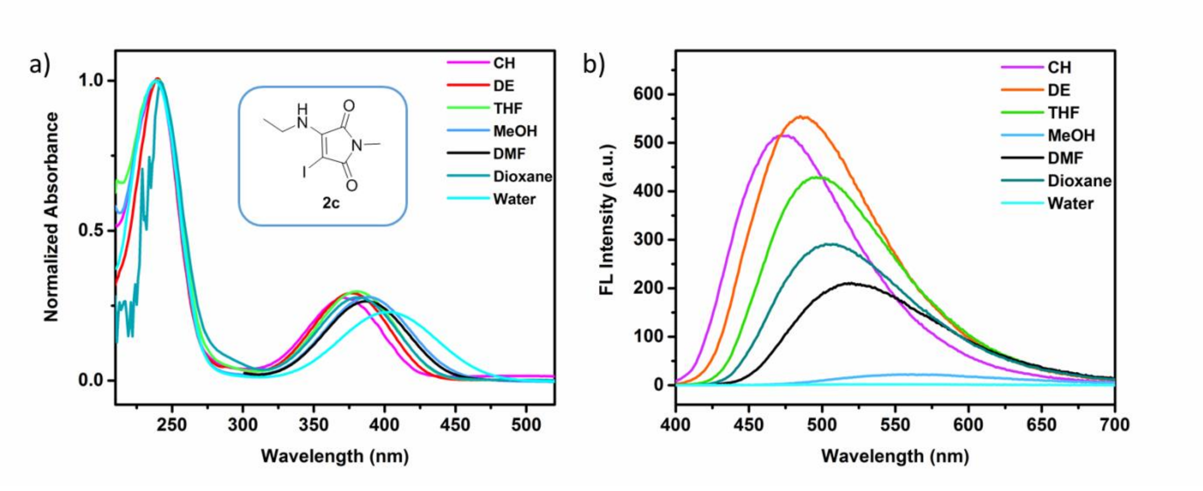

Figure S9. Absorption (a) and emission (b) spectra of **2c** in different solvents at 10 µM.

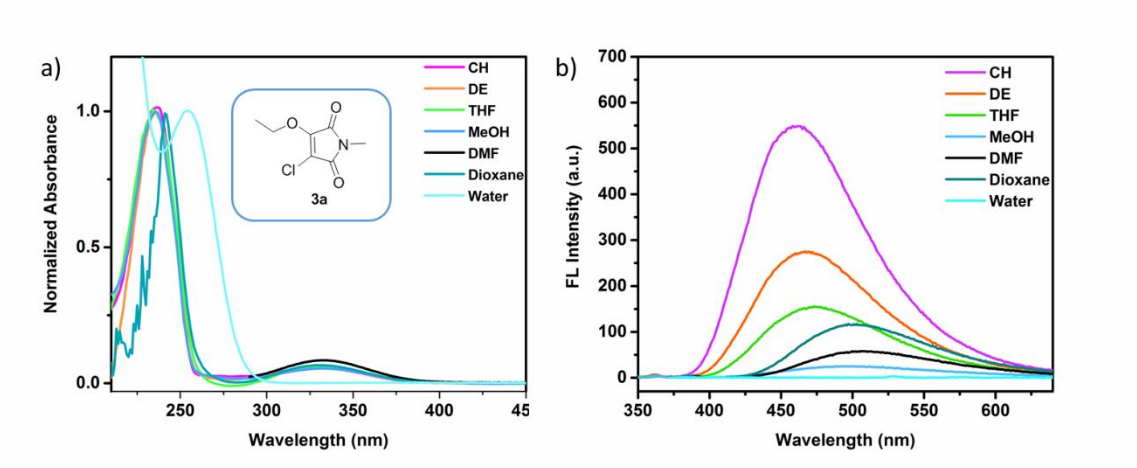

Figure S10. Absorption (a) and emission (b) spectra of **3a** in different solvents at 10 μM.

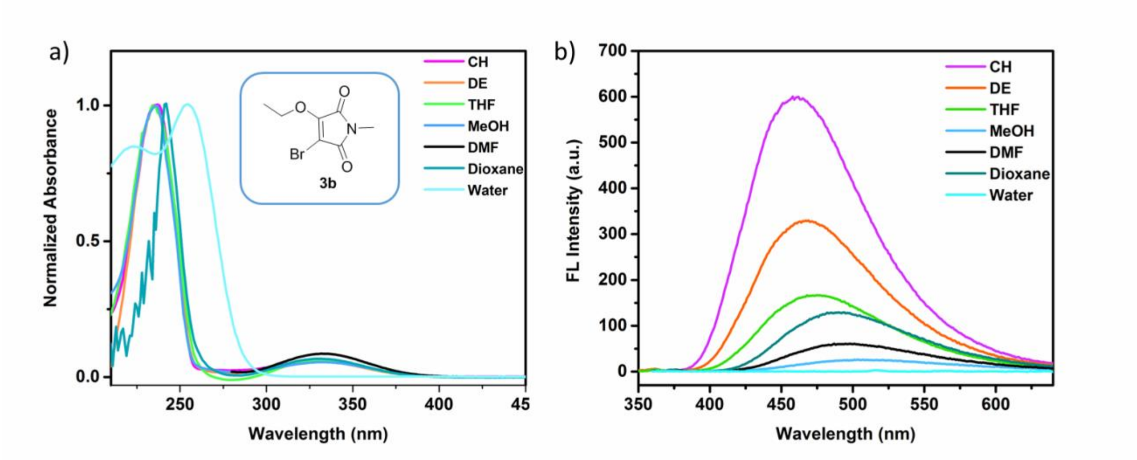

Figure S11. Absorption (a) and emission (b) spectra of **3b** in different solvents at 10 μM.

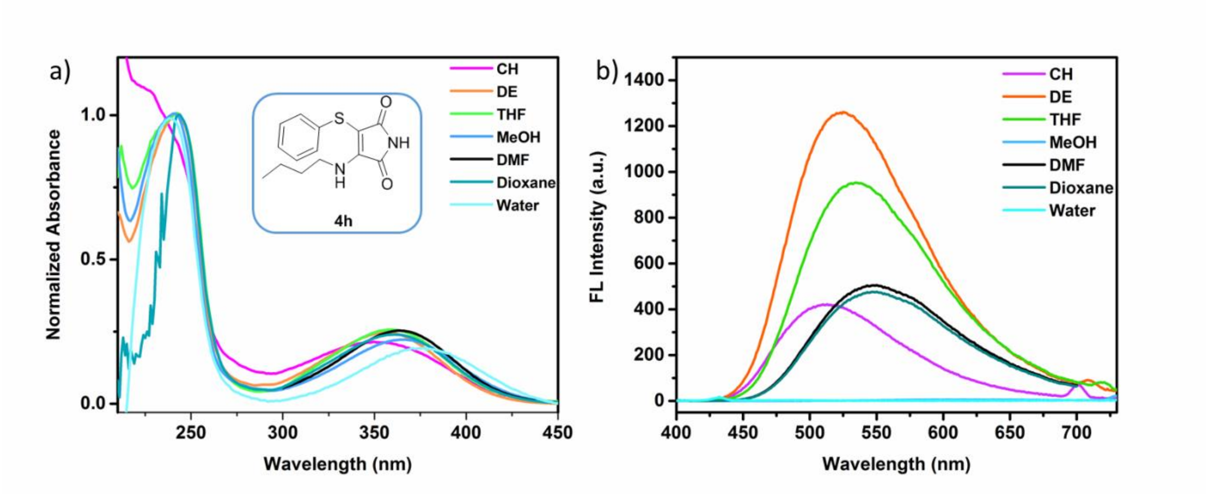

Figure S12. Absorption (a) and emission (b) spectra of **4h** in different solvents at 10 μM.

6. PHOTOGRAPHS IN DIFFERENT SOLVENTS UNDER UV LIGHT

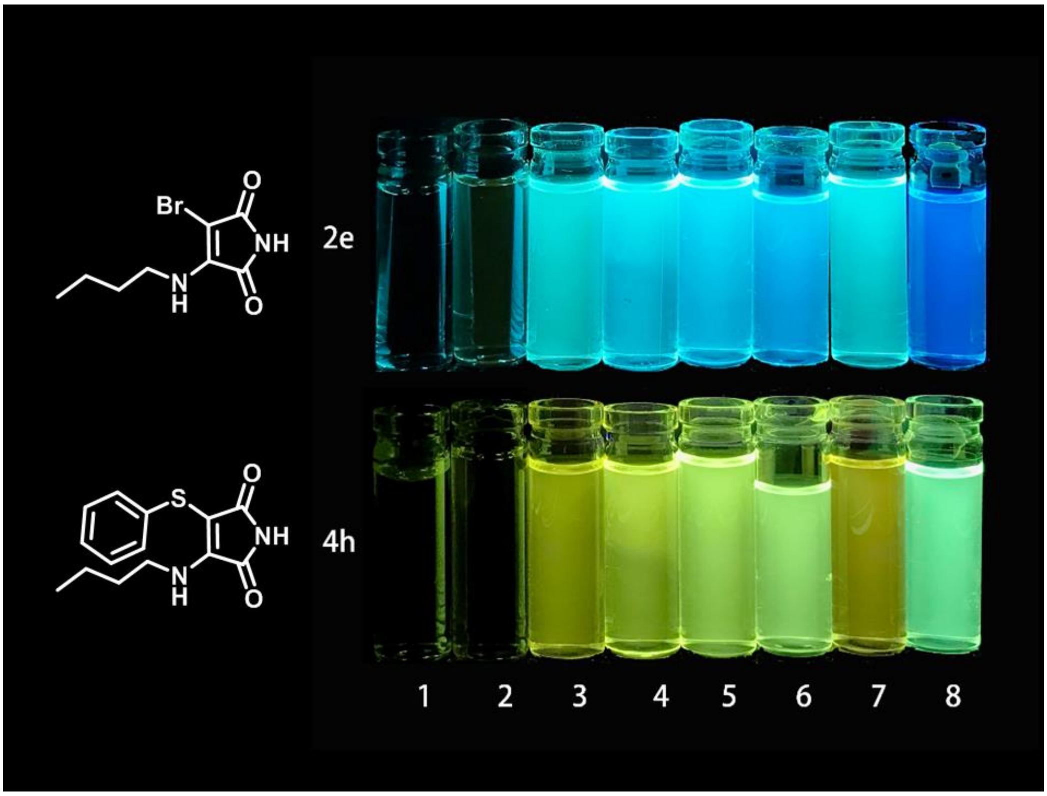

Figure S13. Photographs of ABM (**2e**) and ATM (**4h**) in different solvents under the 365 nm hand-held UV lamp. 1-8: water, methanol, DMF, dioxane, THF, diethyl ether, CH_2Cl_2 and cyclohexane.

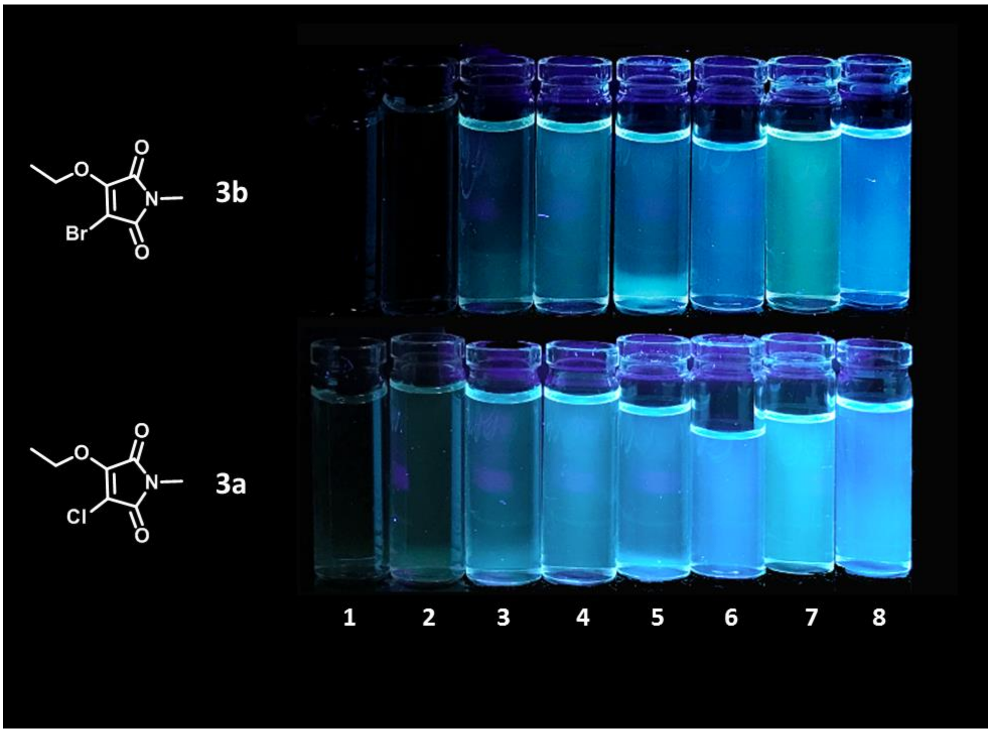

Figure S14. Photographs of OBM (**3b**) and OCM (**3a**) in different solvents under the 365 nm hand-held UV lamp. 1-8: water, methanol, DMF, dioxane, THF, diethyl ether, CH_2Cl_2 and cyclohexane.

7. DSE EFFECT OF SECOND THIOL SUBSTITUTED AMINO MALEIMIDES

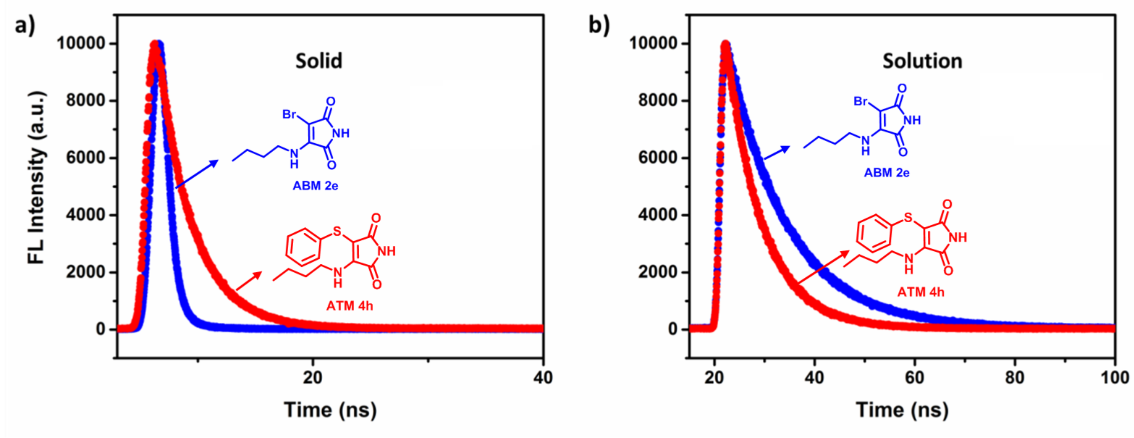

Figure S15. Fluorescent lifetimes of ATM (**4h**) and ABM (**2e**) in solid and solution states (10 µM in dioxane).

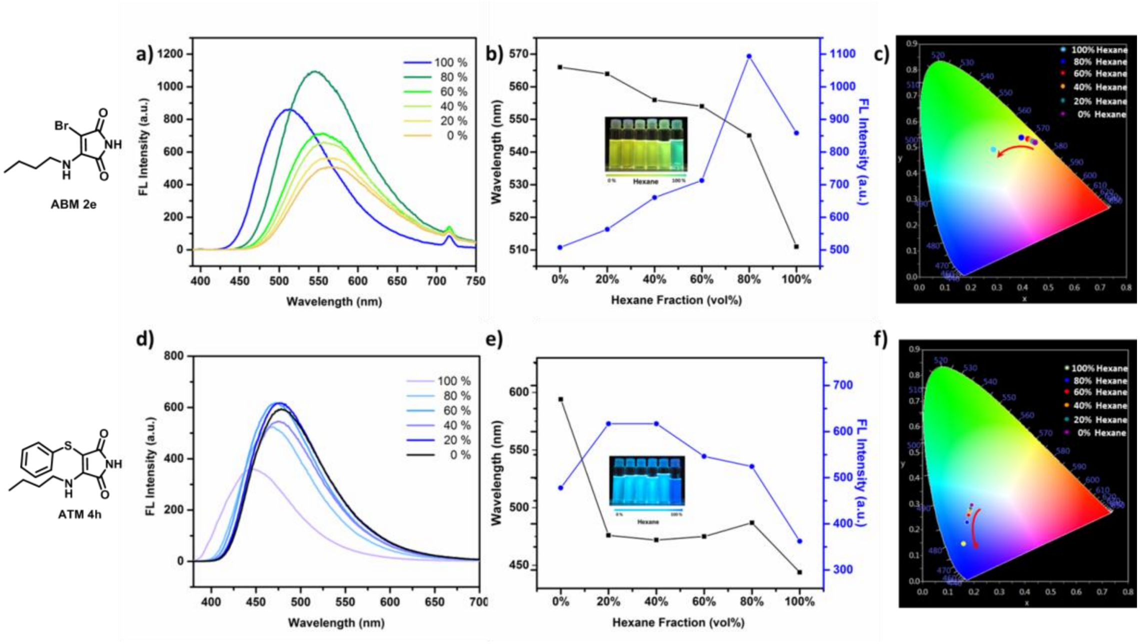

Figure S16. a) Fluorescence spectra of thiophenol substituted aminomaleimide (**4h**) in different ratios of hexane/CH_2Cl_2 solution (0-100% hexane); b) Intensity and wavelength changes in different ratios of hexane/CH_2Cl_2 solutions; (insert images are **4h** under UV light in the solutions); (20 µM, λ_{ex} 358 nm, slit width ex:5 nm, em:5 nm); c) CIE diagram of the fluorescent wavelength shift for **4h** upon changing the solvent quality. d) Fluorescence spectra of aminobromomaleimide (**2e**) in different ratios of hexane/CH_2Cl_2 solution (0-100% hexane); e) Intensity and wavelength changes in different ratios of hexane/CH_2Cl_2 solutions; (insert images are **2e** under UV light in the solutions); (20 µM, λ_{ex} 362 nm, slit width ex:5 nm, em:5 nm); f) CIE diagram of fluorescent wavelength shift for **2e** upon changing the solvent quality.

8. NMR SPETRA OF SUBSTITUTED MALEIMIDES

3-chloro-4-(ethylamino)-1-methyl-1H-pyrrole-2,5-dione (2a):

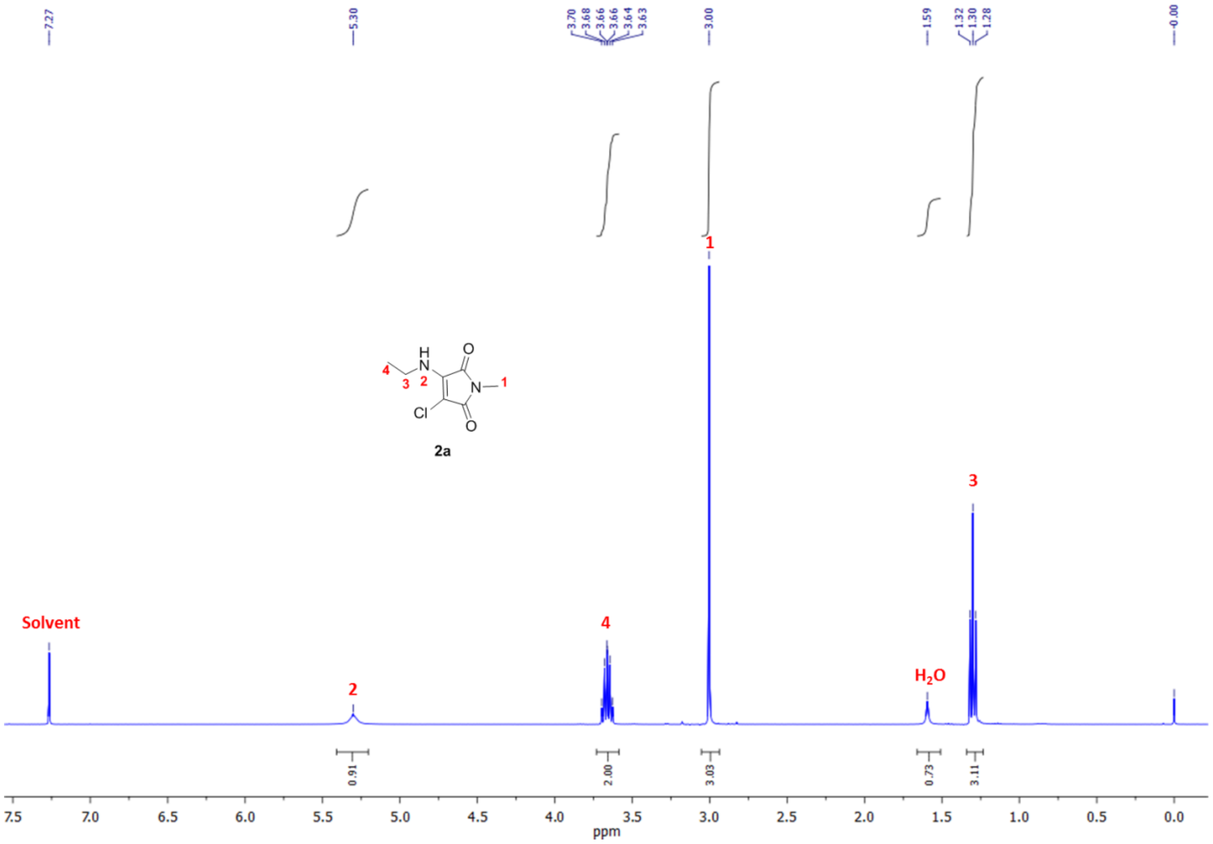

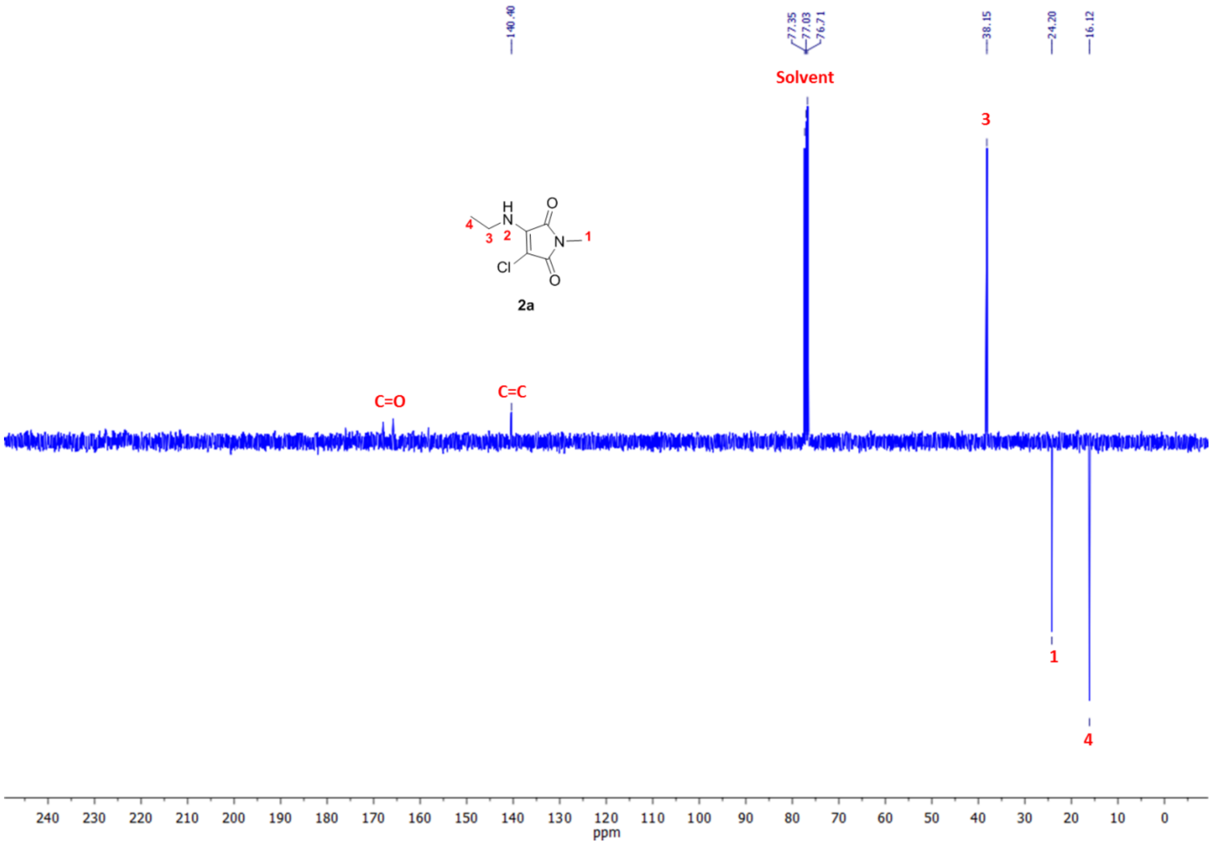

3-bromo-4-(ethylamino)-1-methyl-1H-pyrrole-2,5-dione (2b):

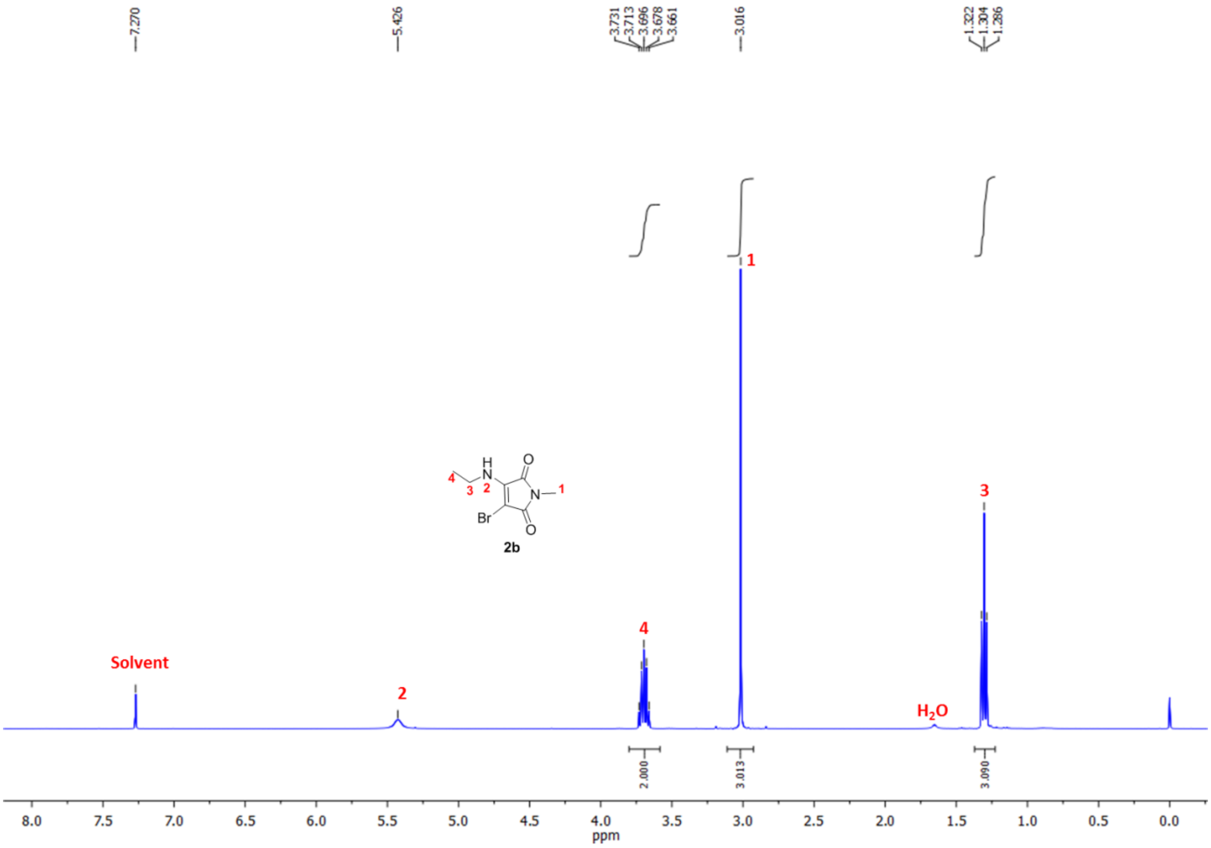

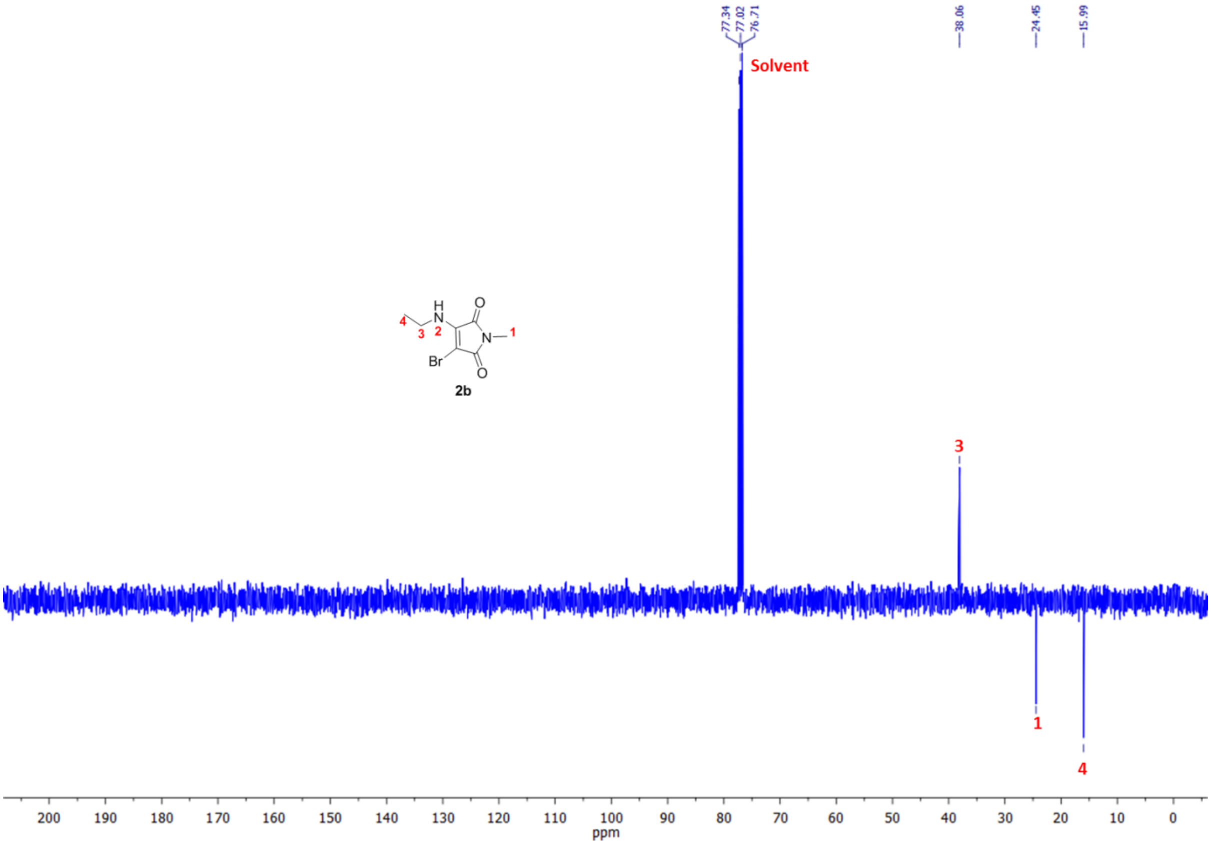

3- iodo-4-(ethylamino)- 1-methyl-1H-pyrrole-2,5-dione (2c):

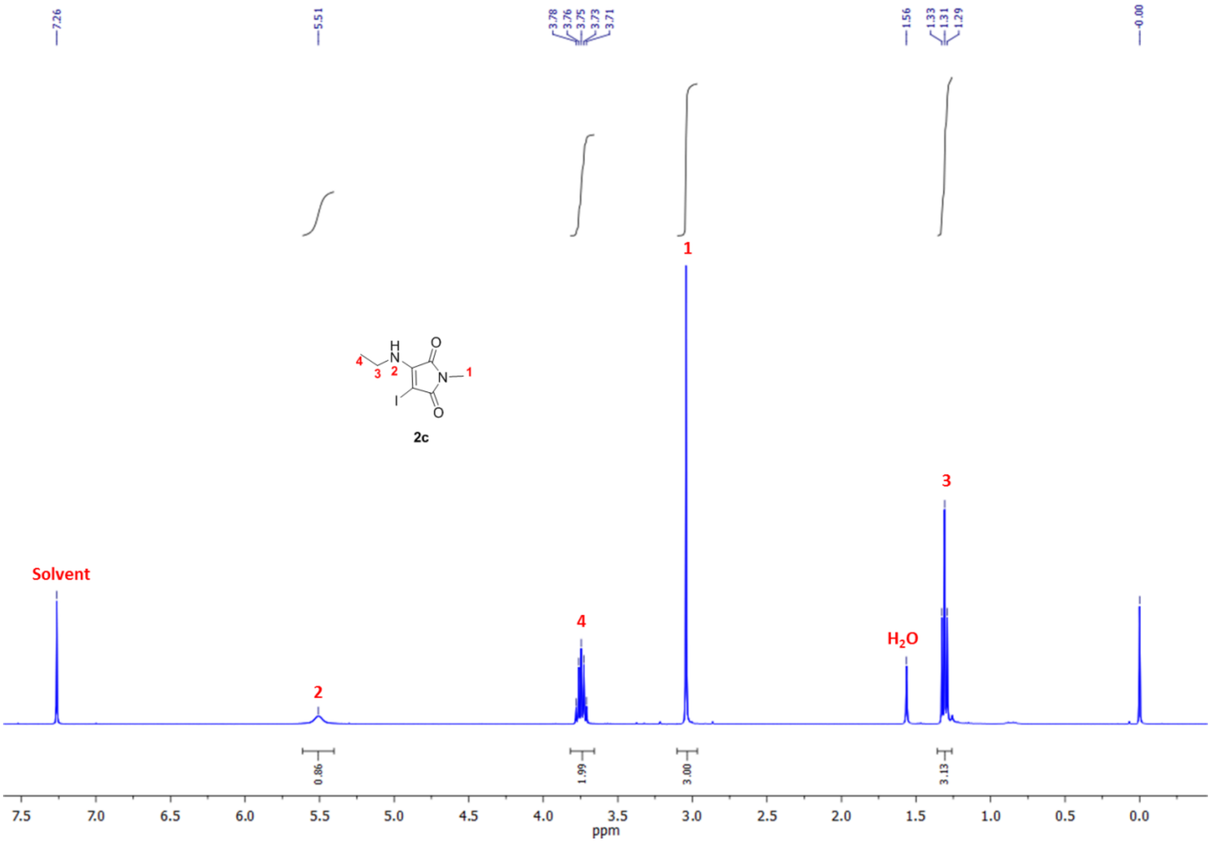

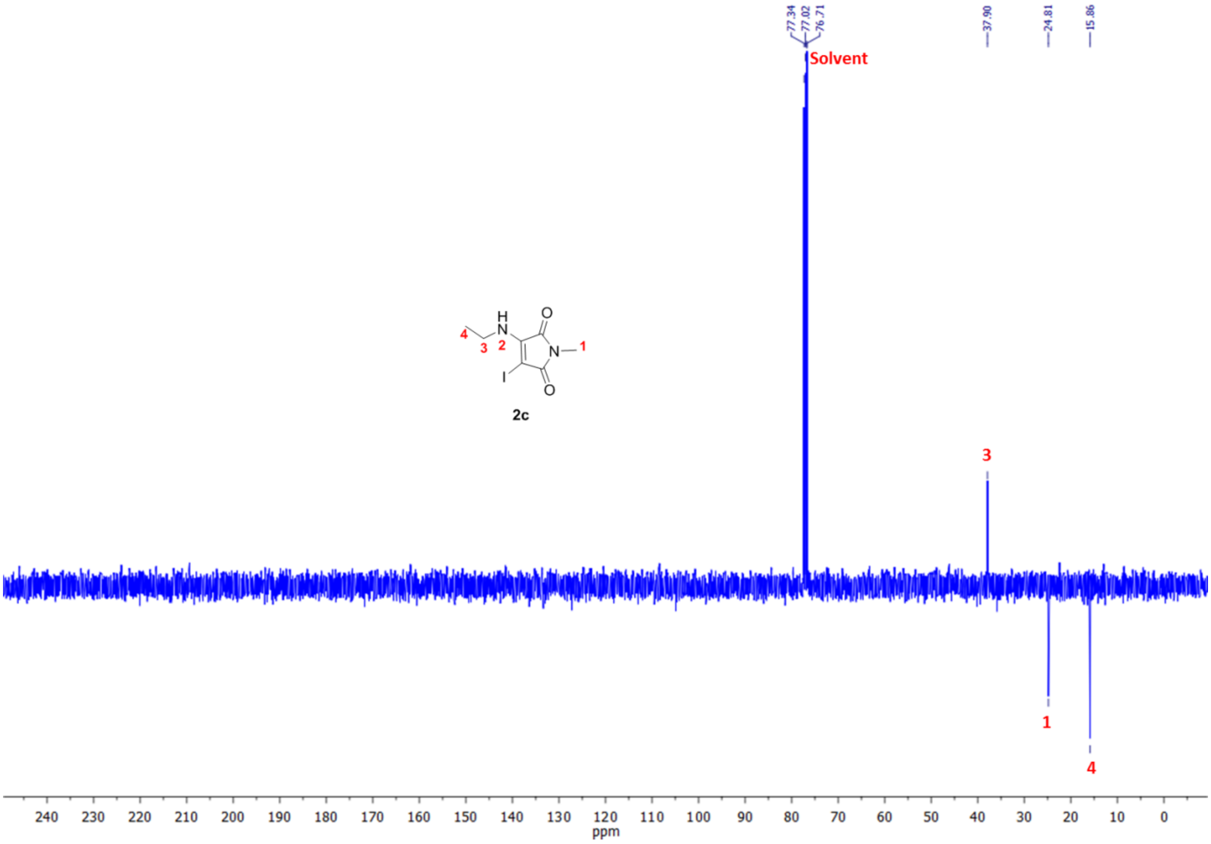

3-(butylamino)-4-chloro-1-methyl-1H-pyrrole-2,5-dione (2d):

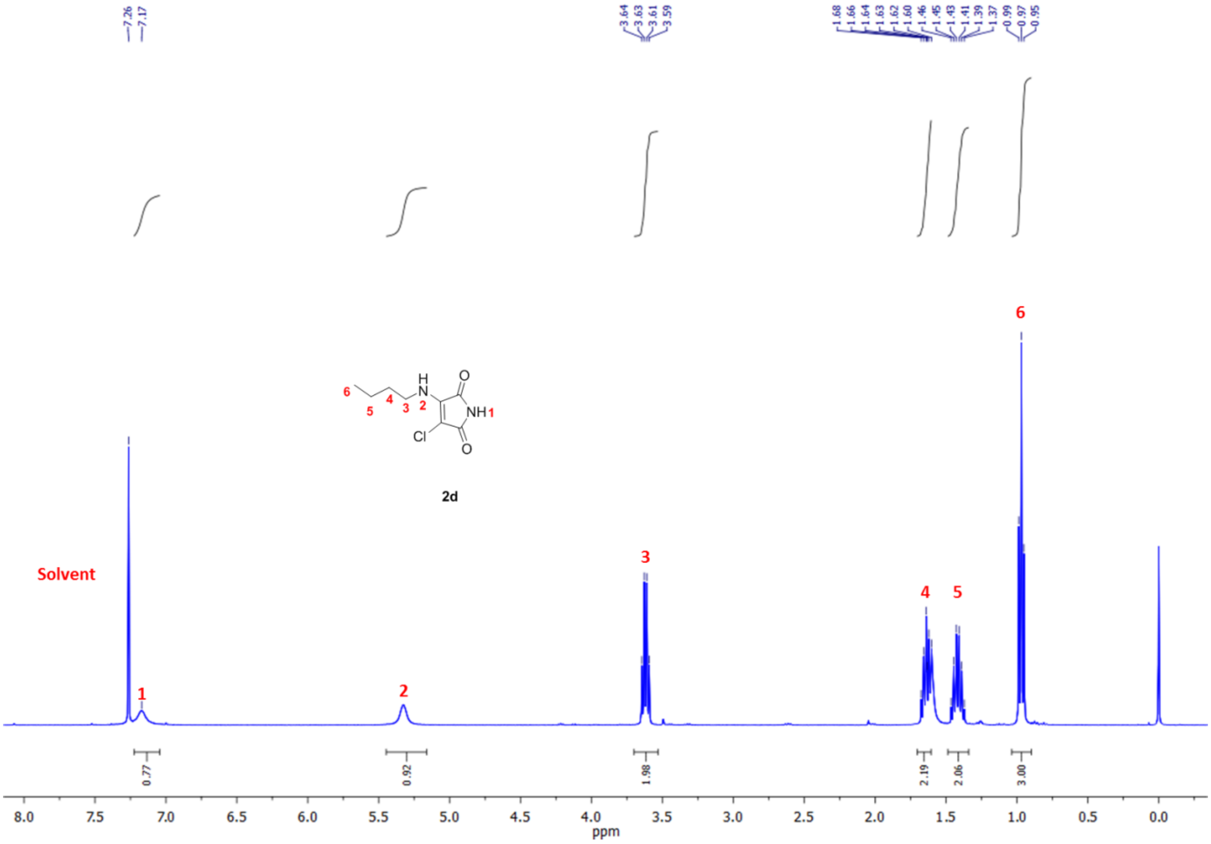

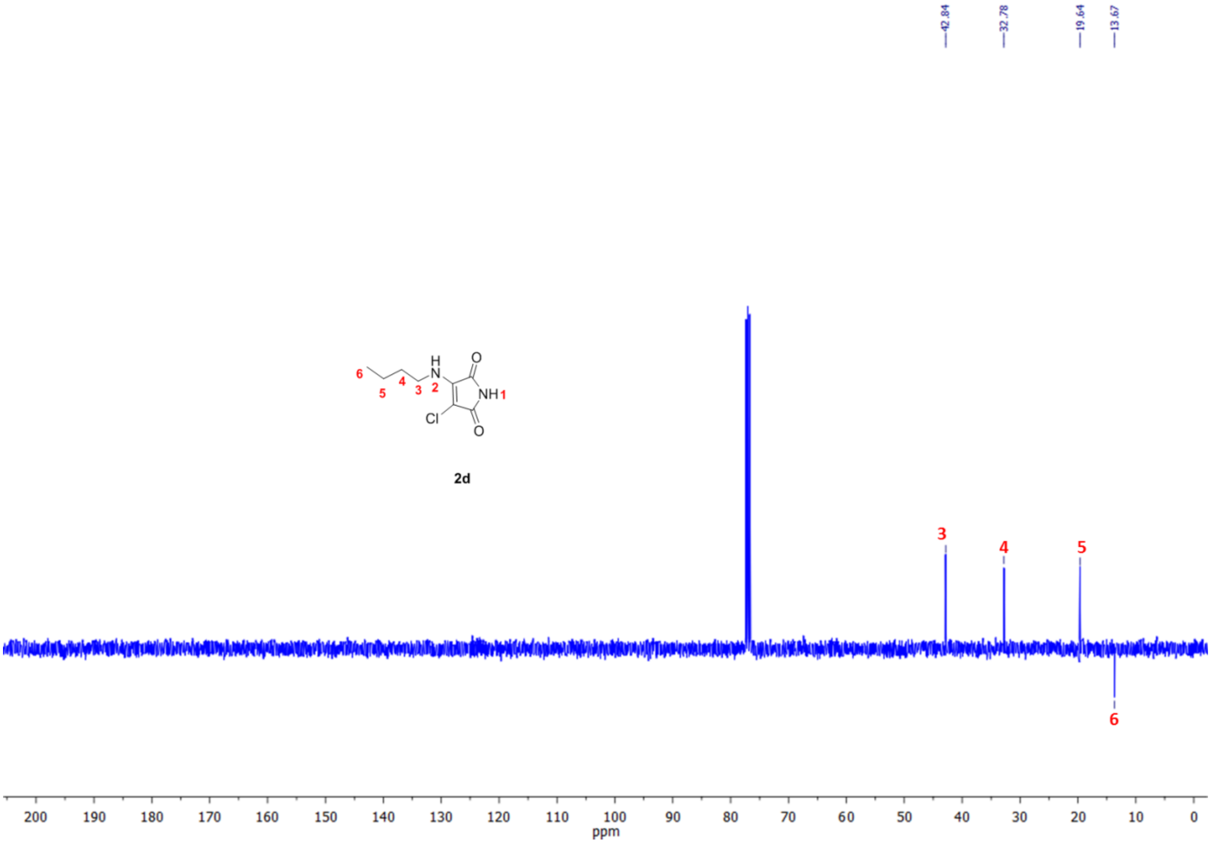

3-bromo-4-(butylamino)-1H-pyrrole-2,5-dione (2e)

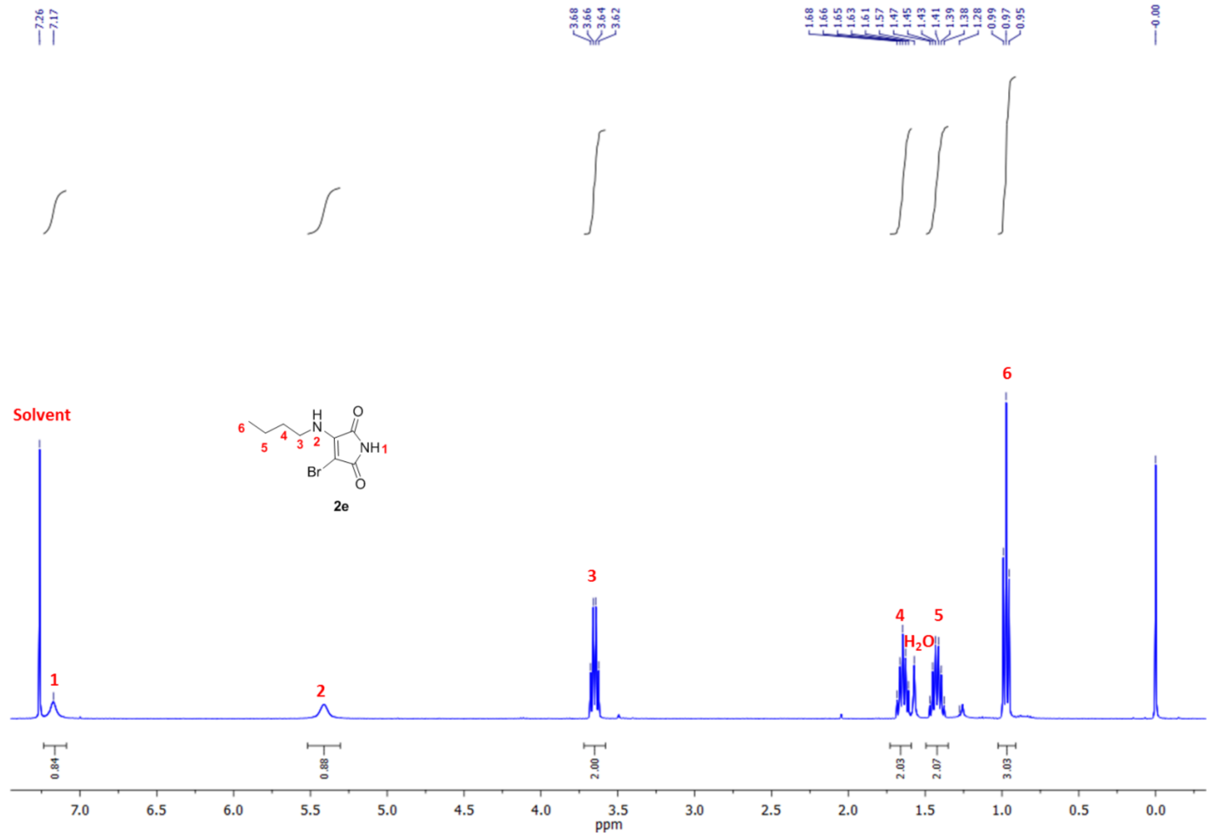

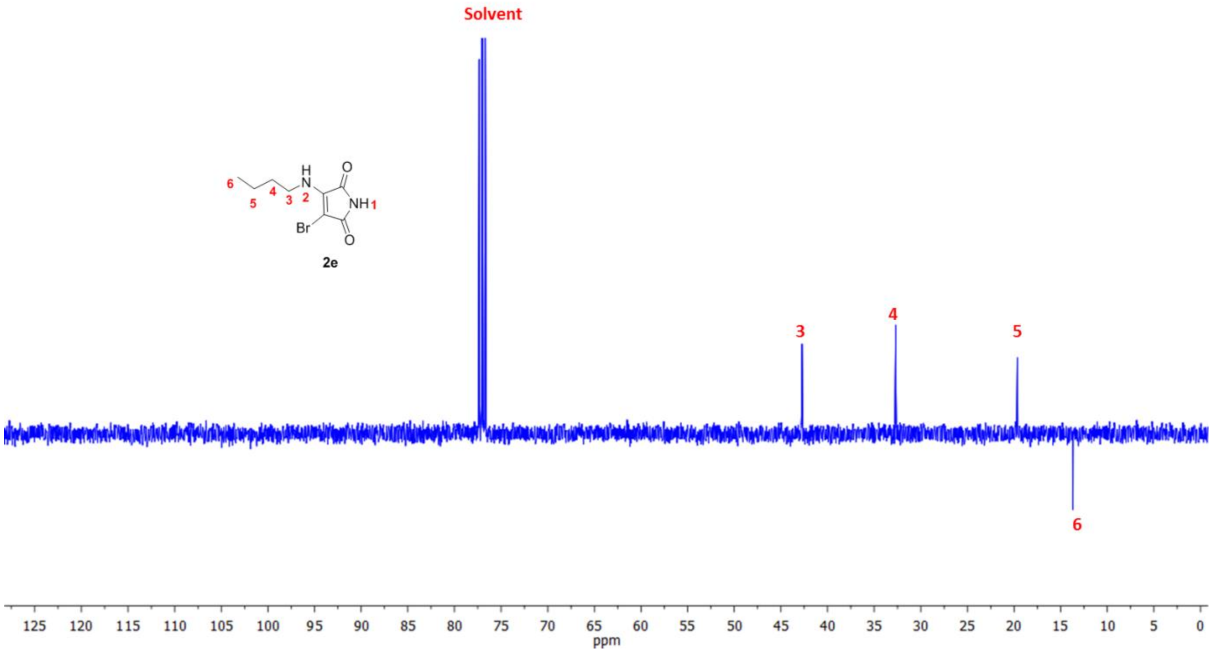

3-chloro-4-ethoxy-1-methyl-1H-pyrrole-2,5-dione (3a)

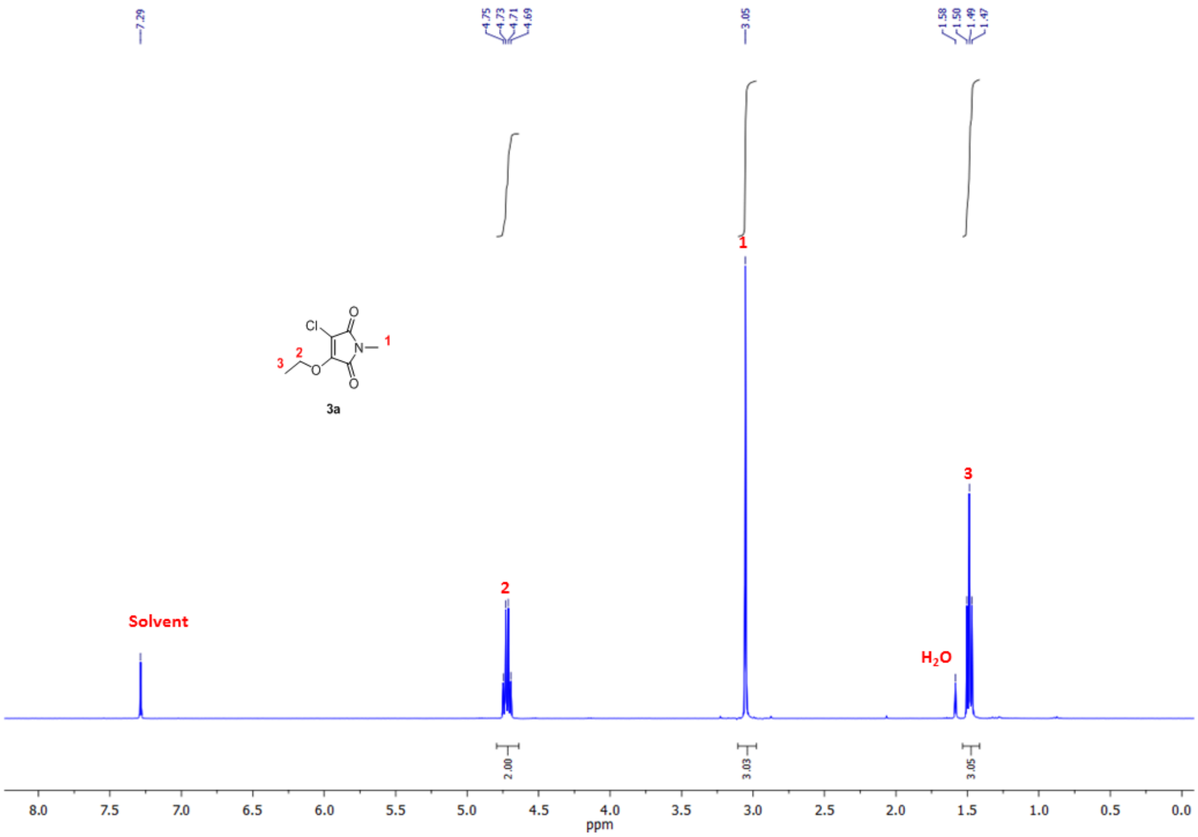

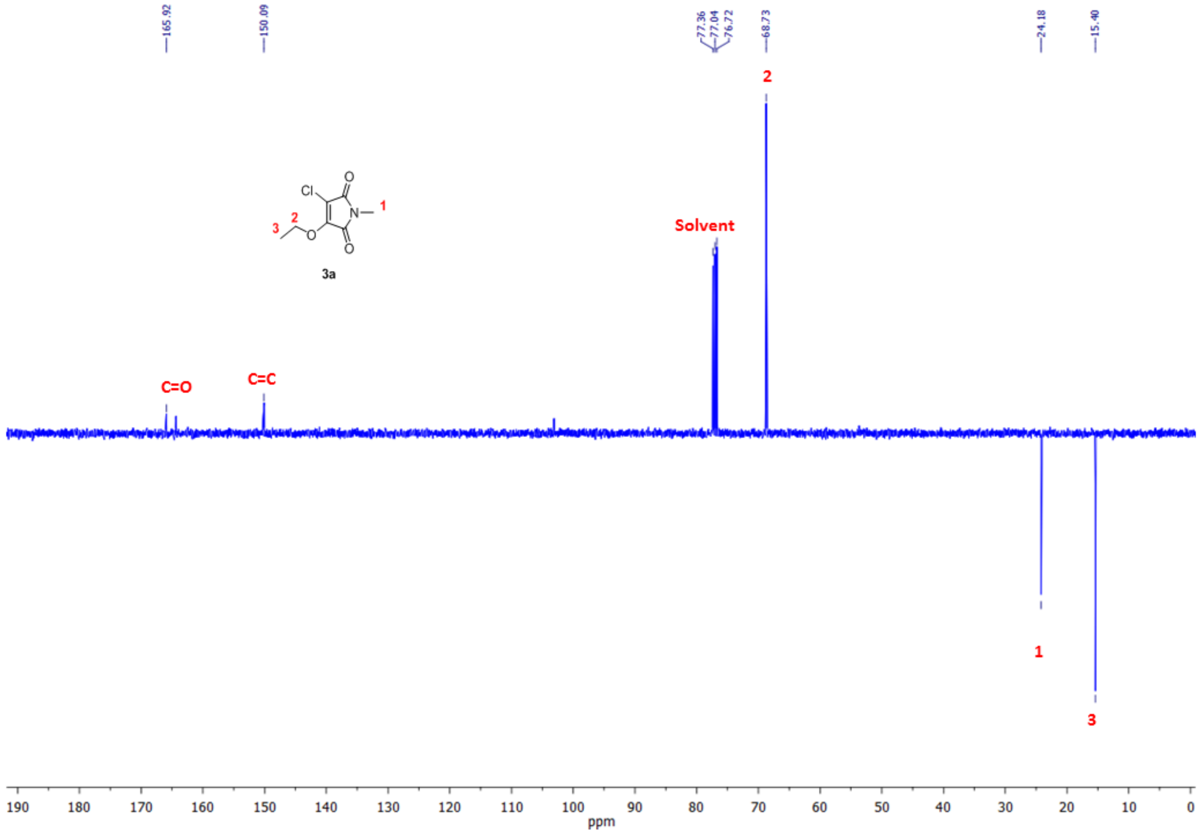

3-bromo-4-ethoxy-1-methyl-1H-pyrrole-2, 5-dione (3b)

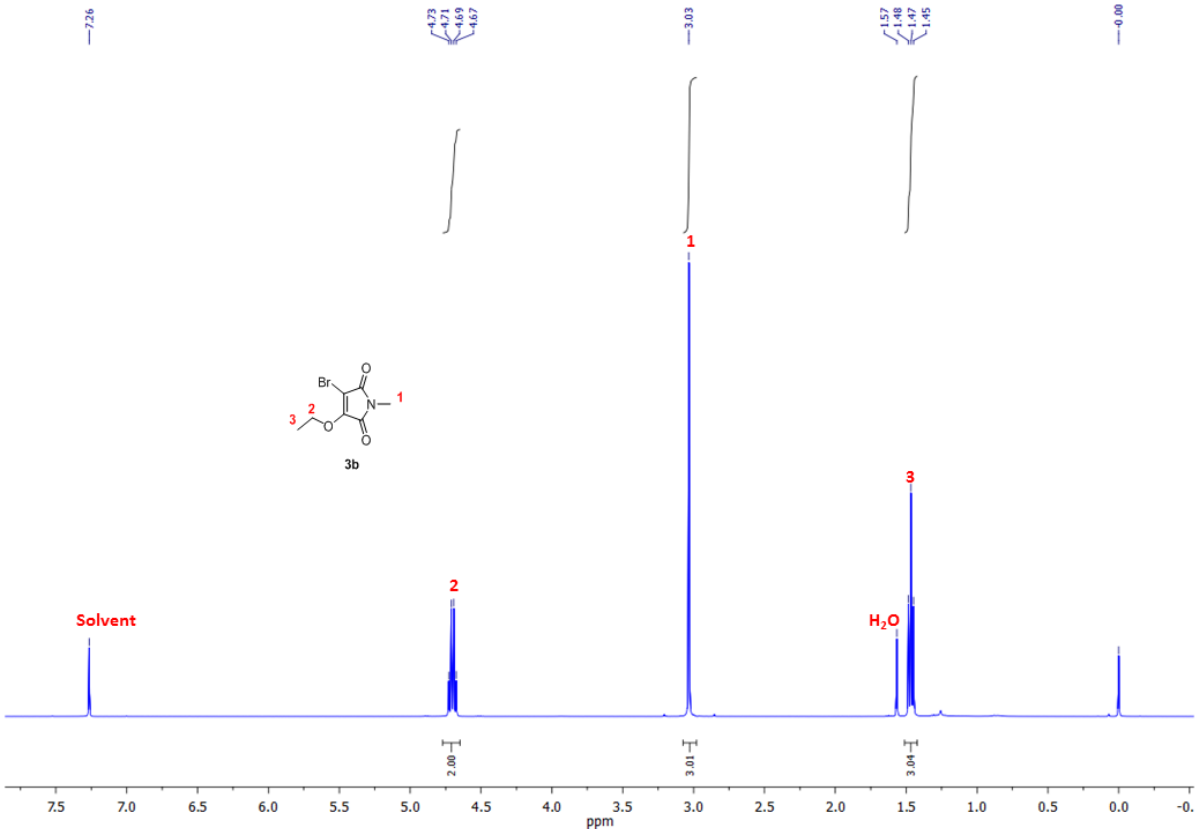

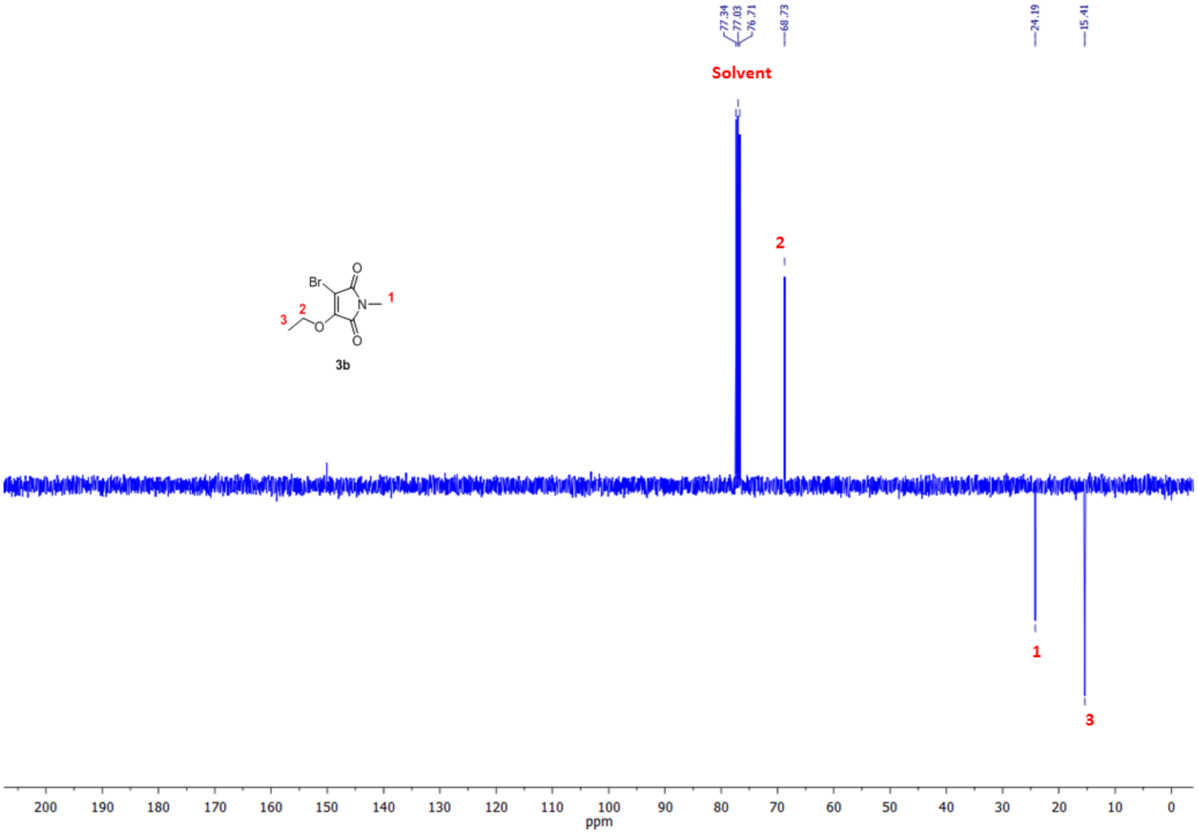

3-(benzyloxy)-4-chloro-1-methyl-1H-pyrrole-2,5-dione (3c):

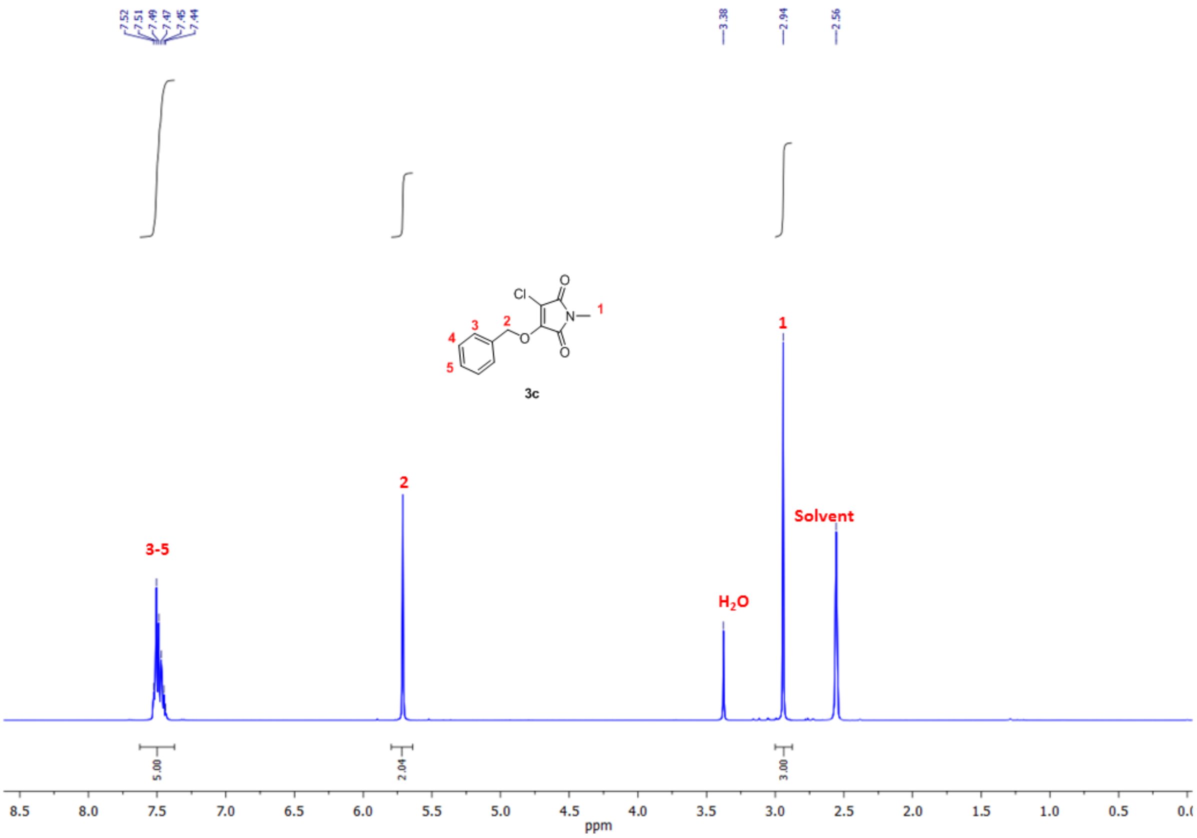

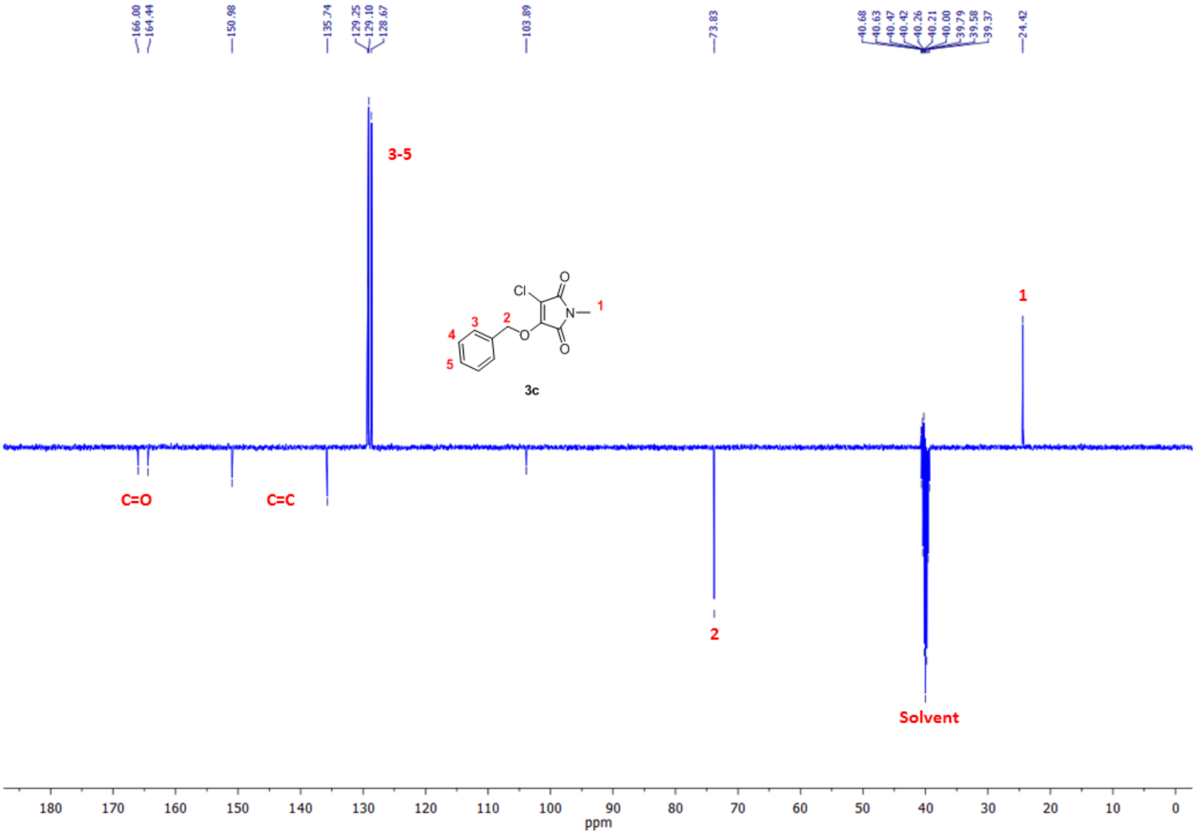

3-(benzyloxy)-4-bromo-1-methyl-1H-pyrrole-2,5-dione (3d)

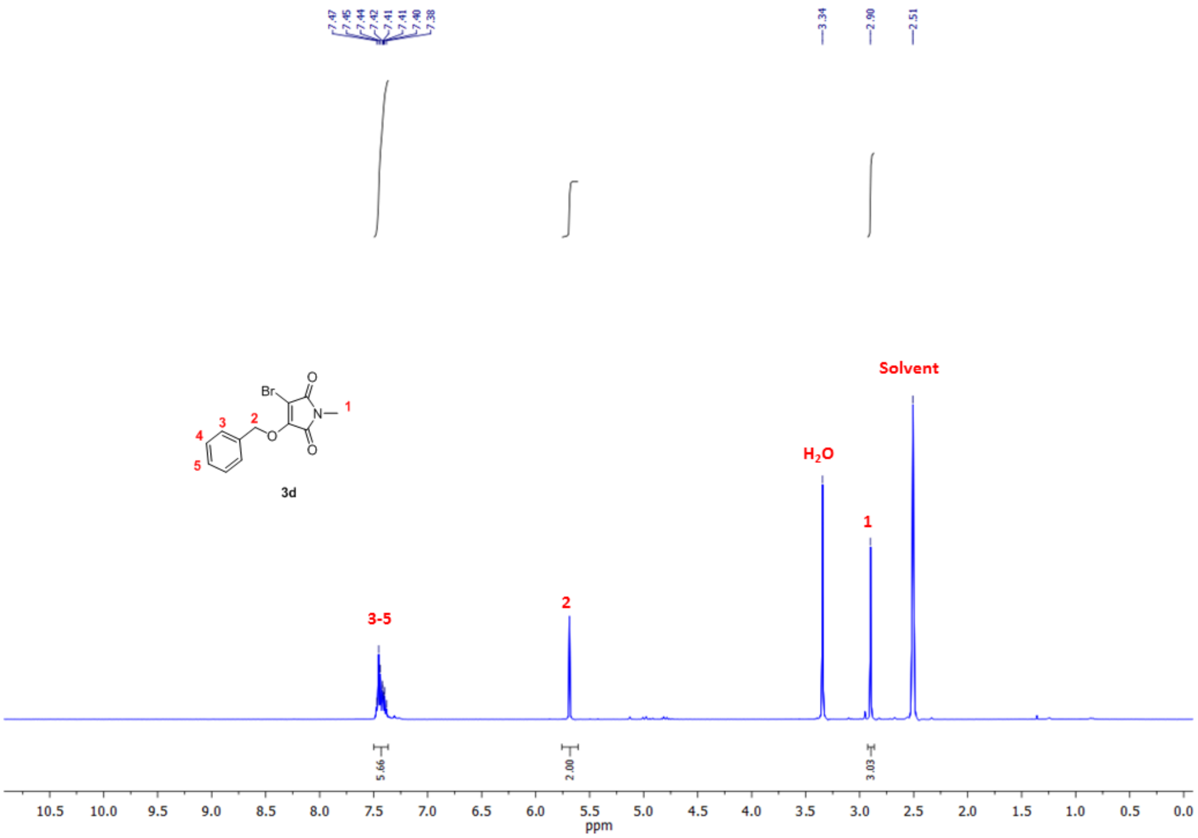

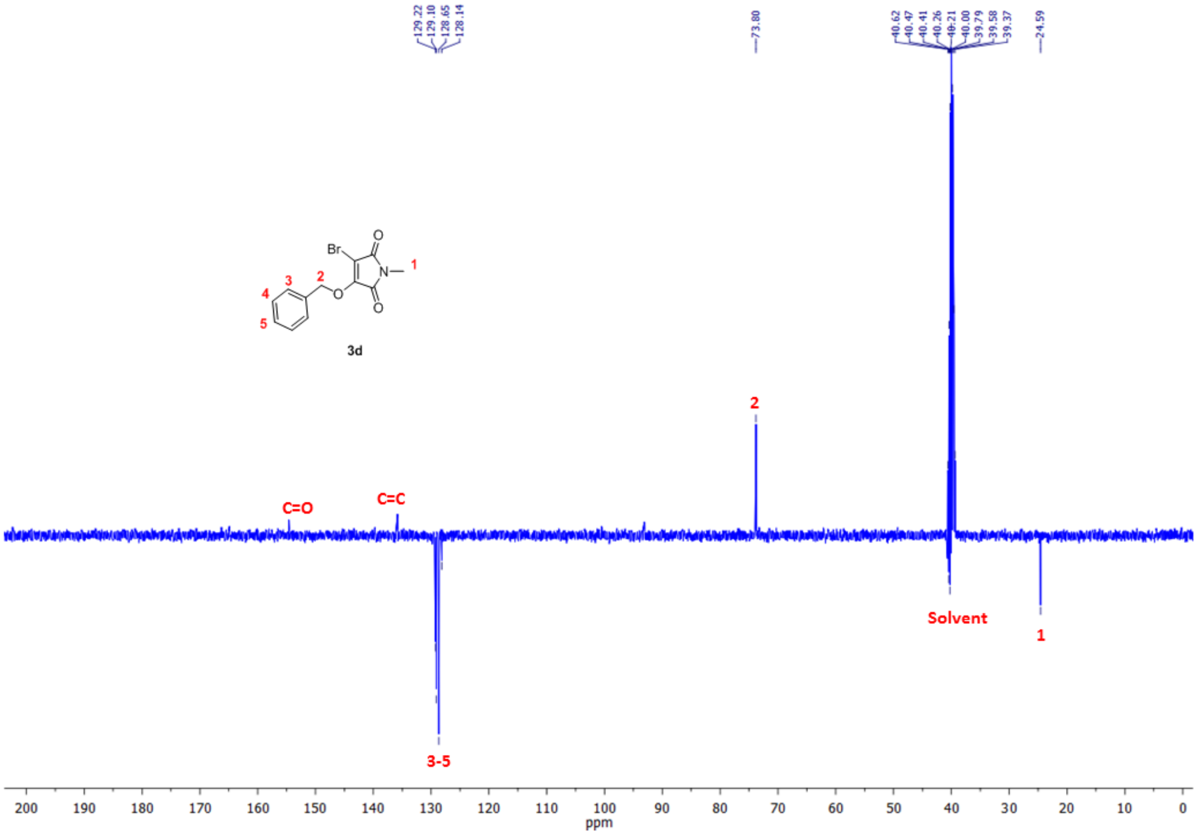

3-chloro-1-methyl-4-phenoxy-1H-pyrrole-2,5-dione (3e):

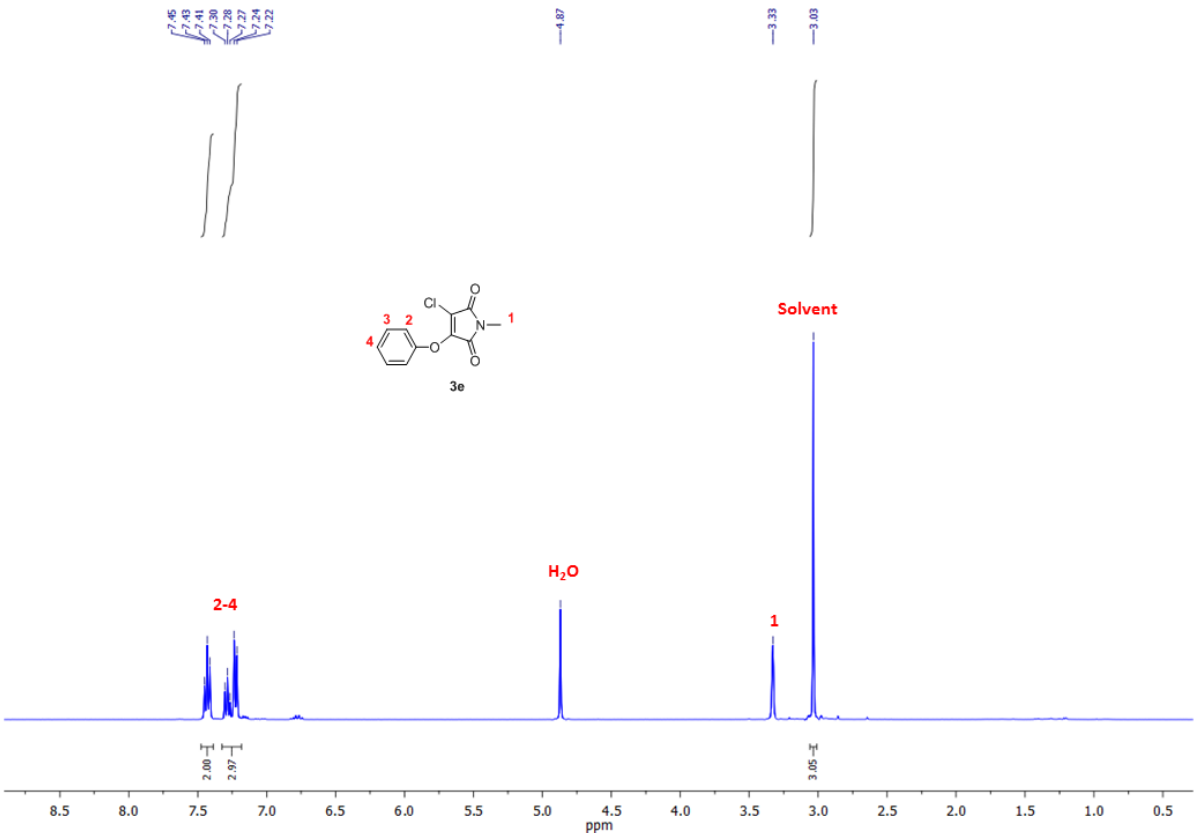

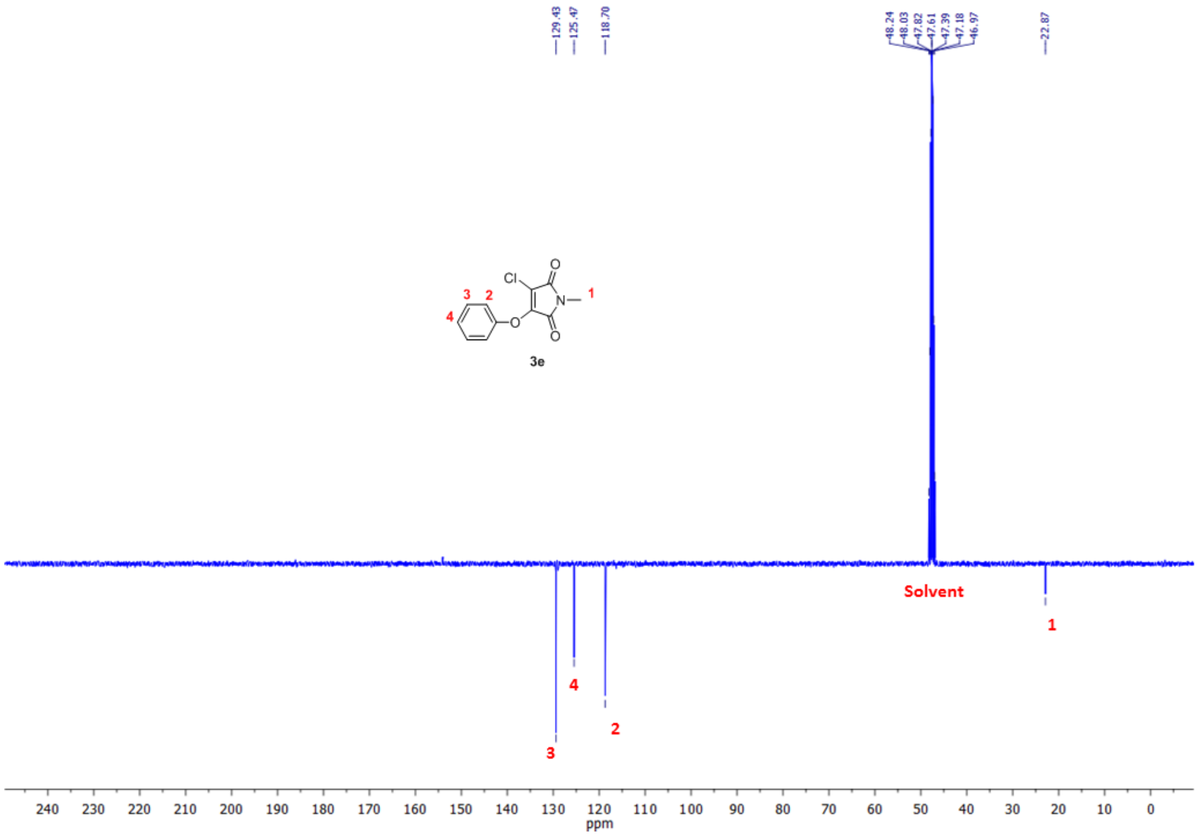

3-bromo-1-methyl-4-phenoxy-1H-pyrrole-2,5-dione (3f):

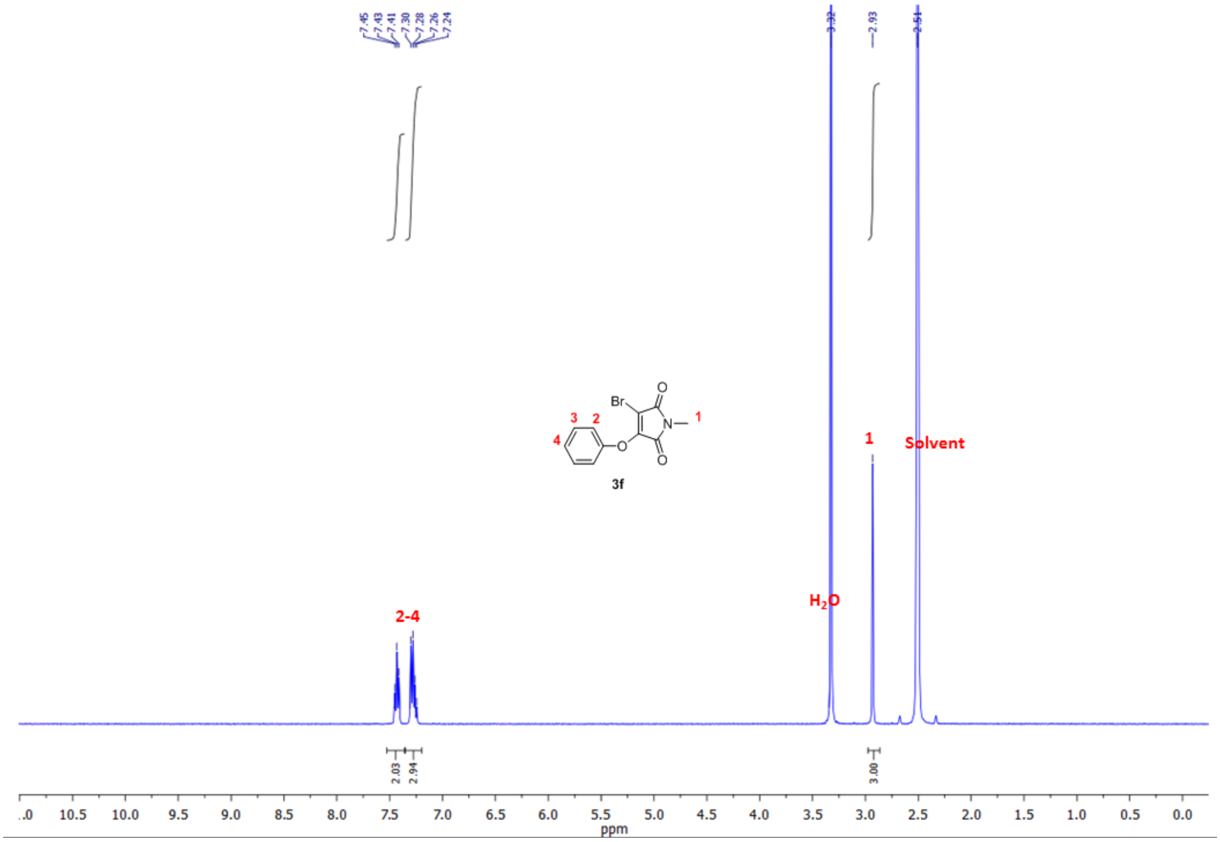

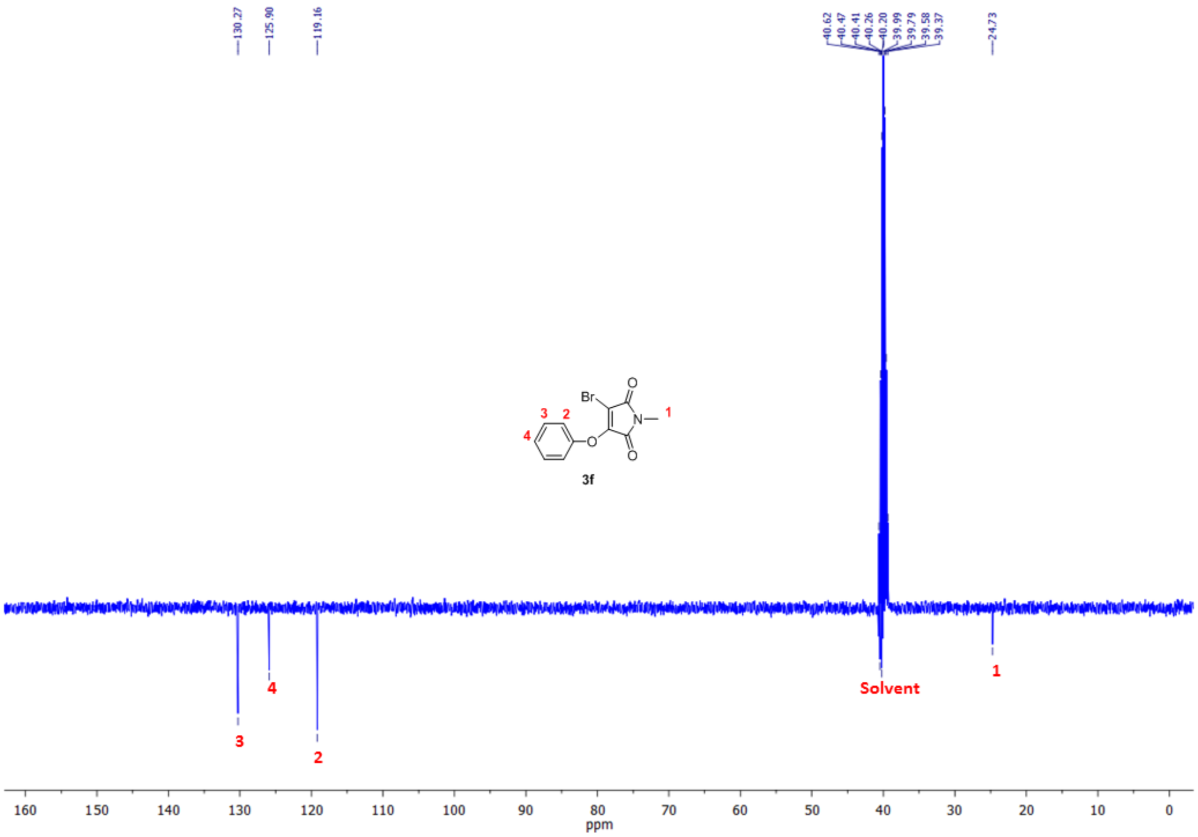

3-(butylthio)-4-(ethylamino)-1-methyl-1H-pyrrole-2,5-dione(4a)

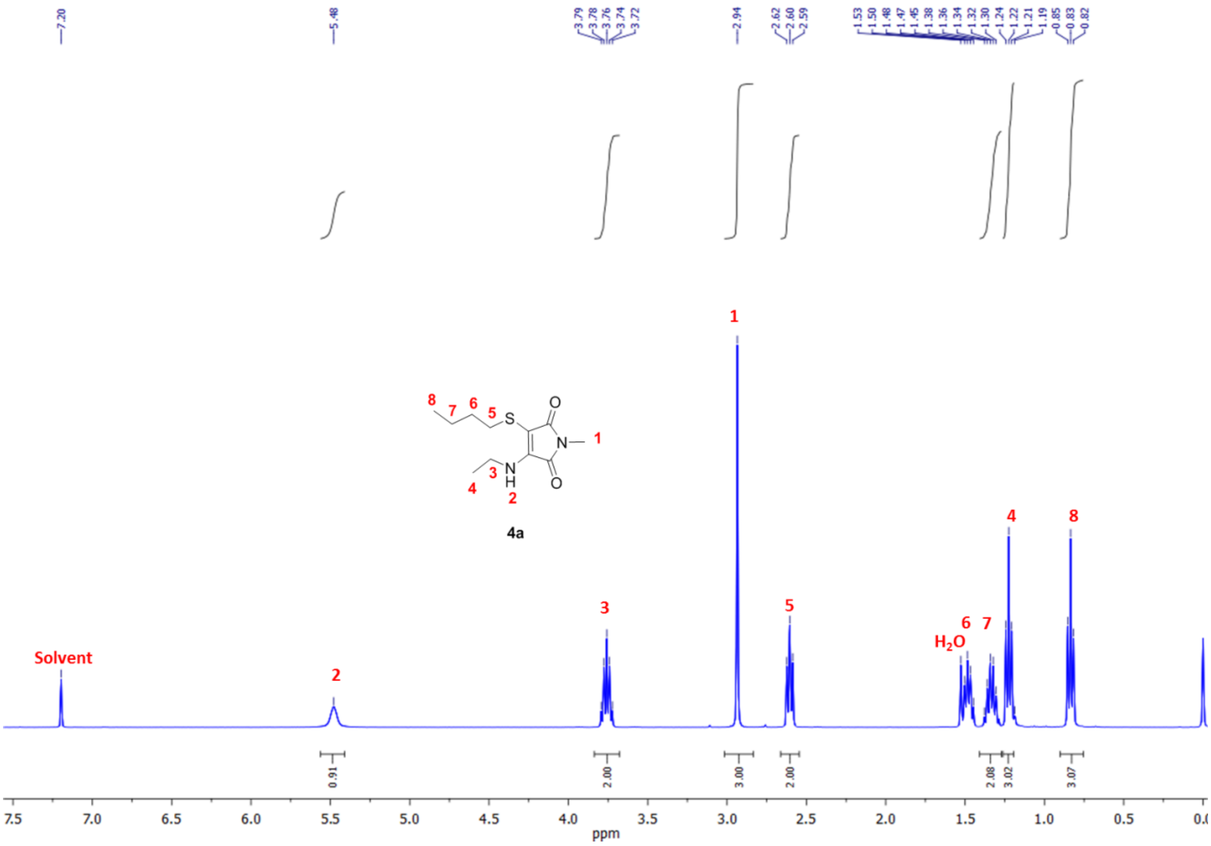

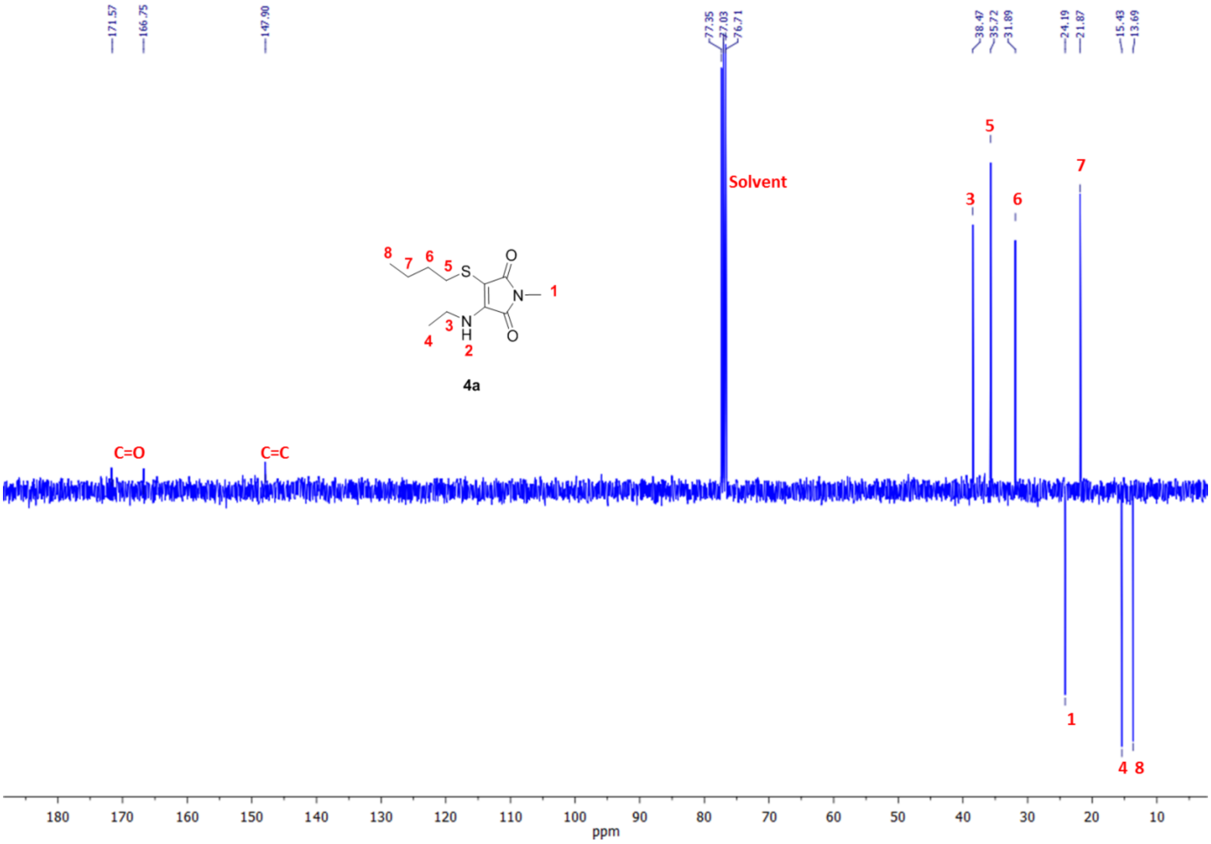

3-(ethylamino)-1-methyl-4-(octadecylthio)-1H-pyrrole-2,5-dione (4b)

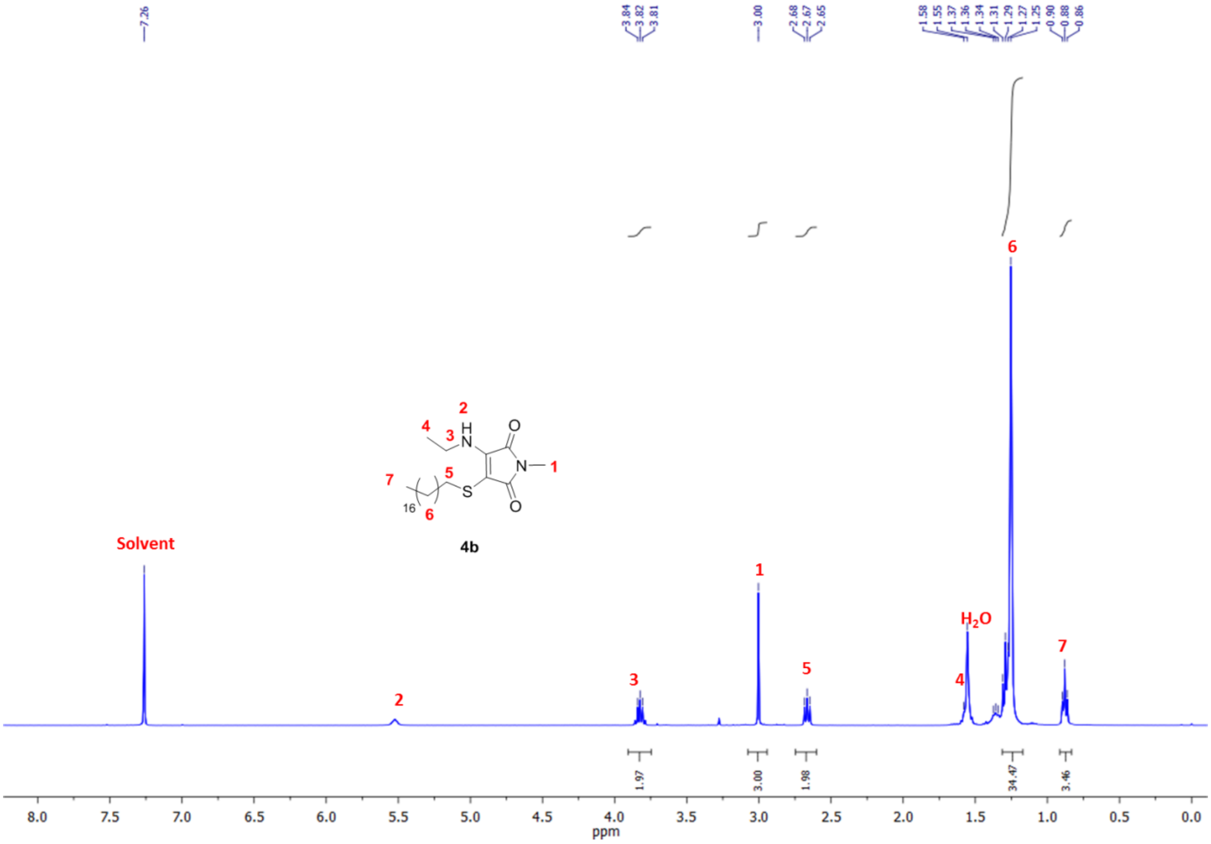

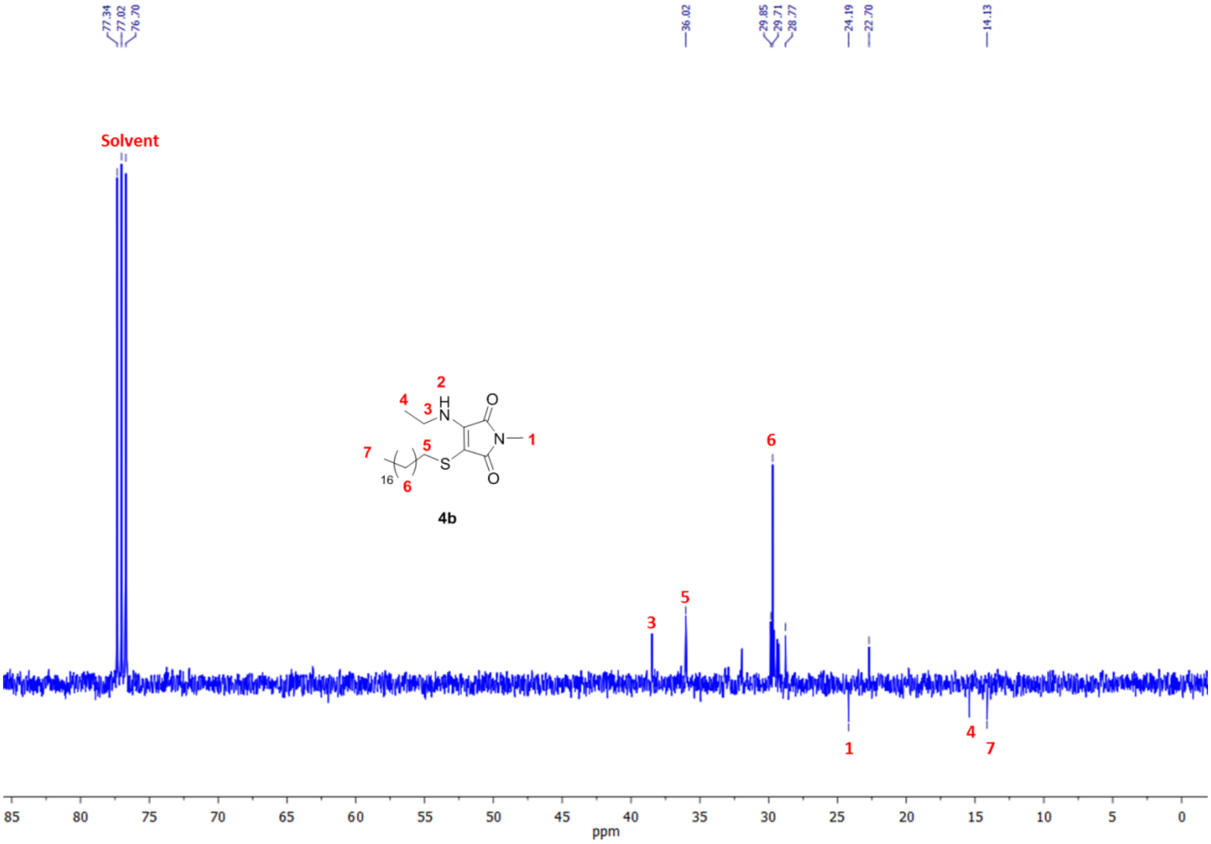

3-(ethylamino)-1-methyl-4-(phenylthio)-1H-pyrrole-2,5-dione(4c)

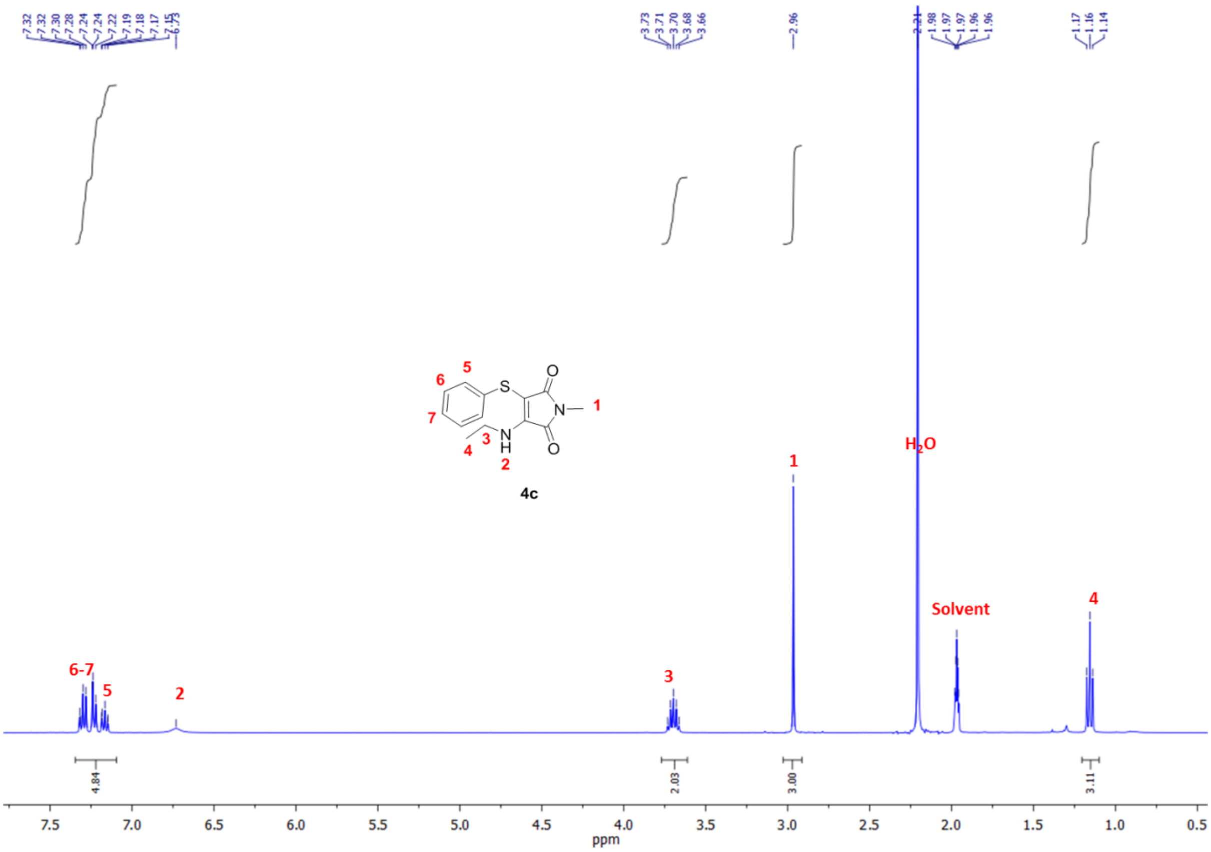

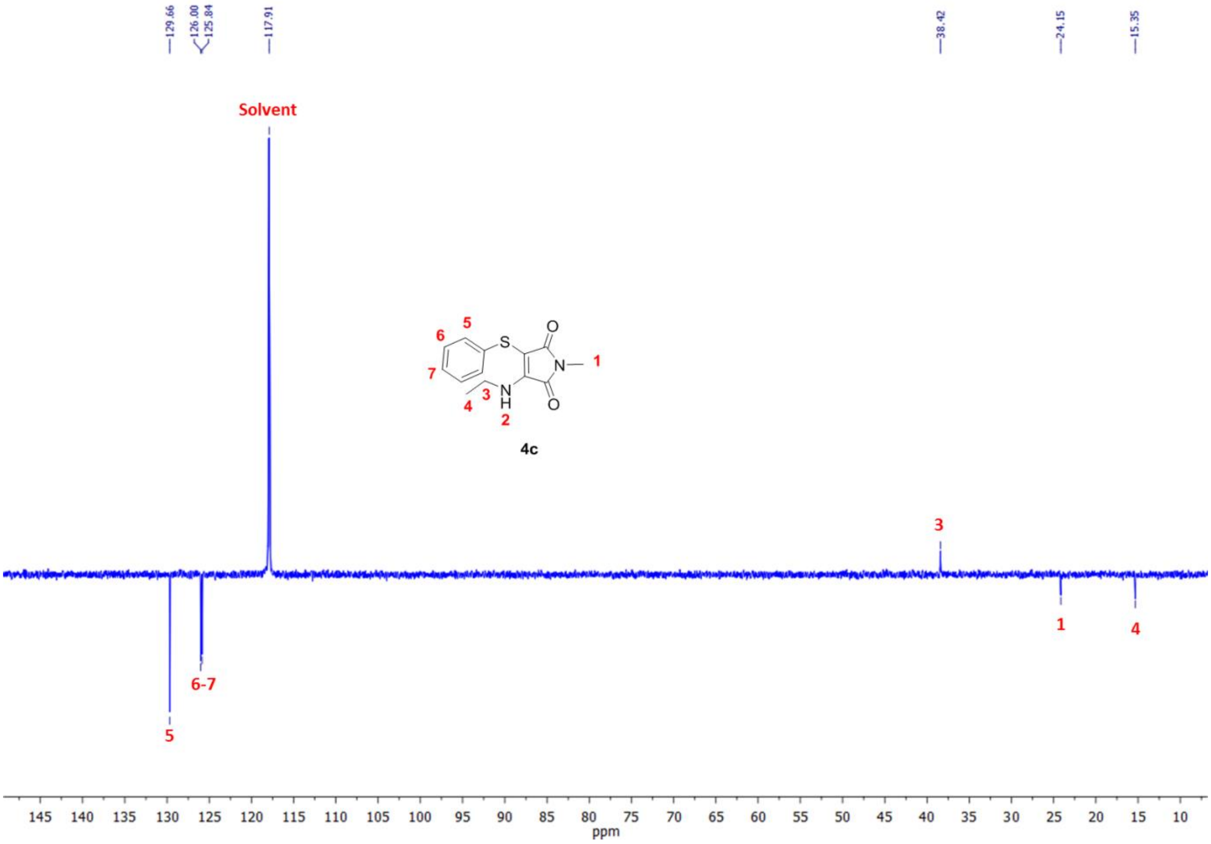

3-(ethylamino)-1-methyl-4-(o-tolylthio)-1H-pyrrole-2,5-dione(4d)

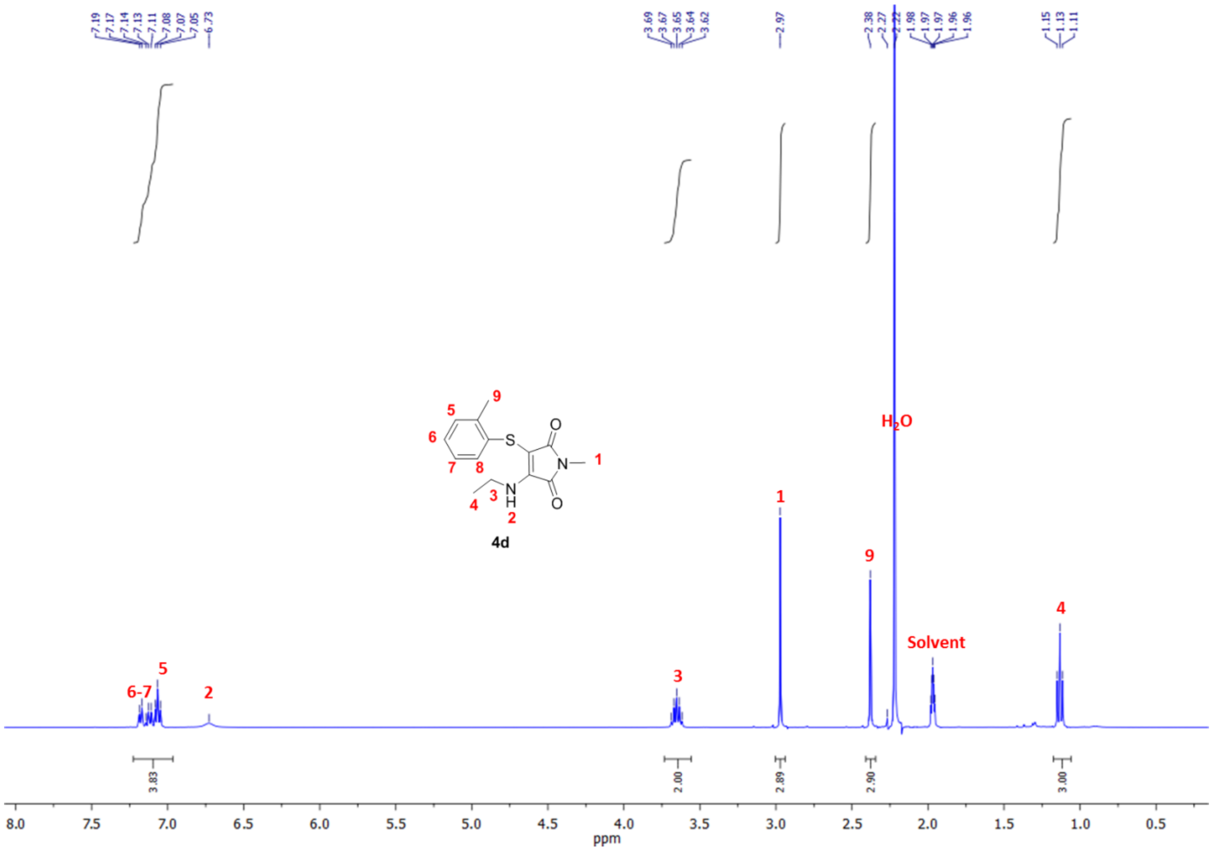

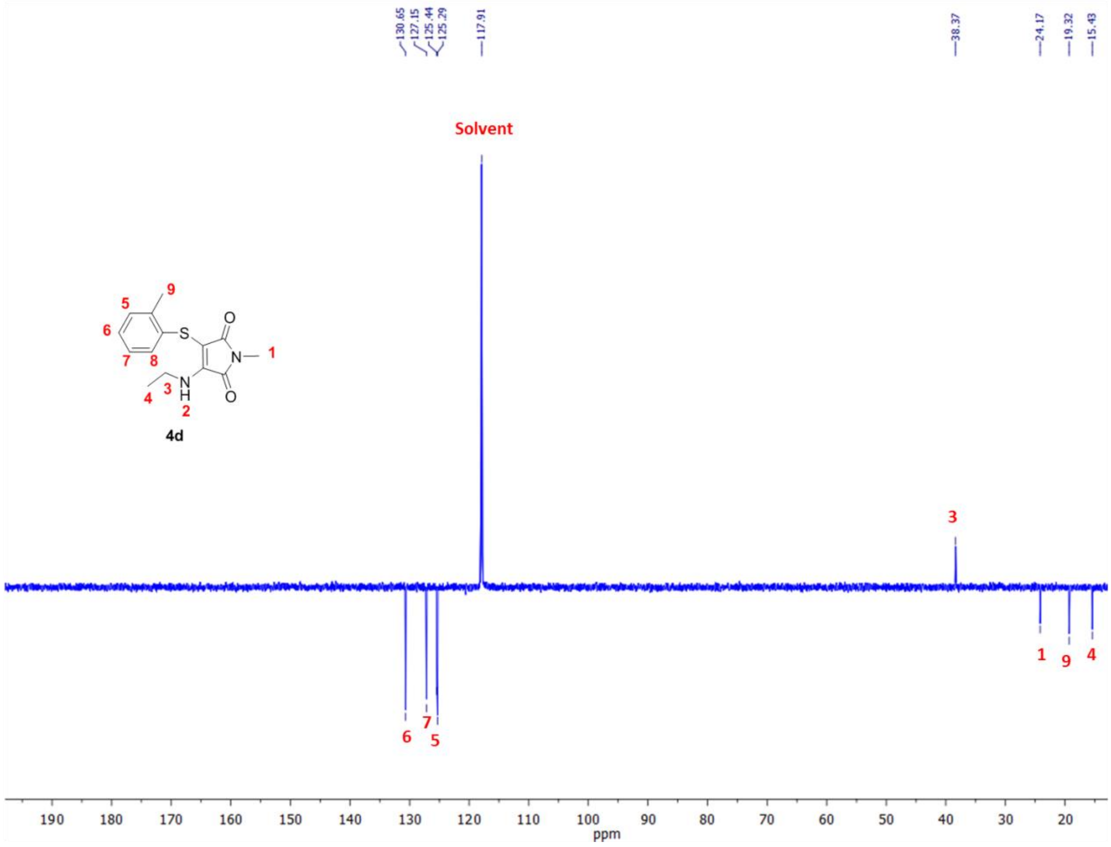

3-(benzylthio)-4-(ethylamino)-1-methyl-1H-pyrrole-2,5-dione (4e)

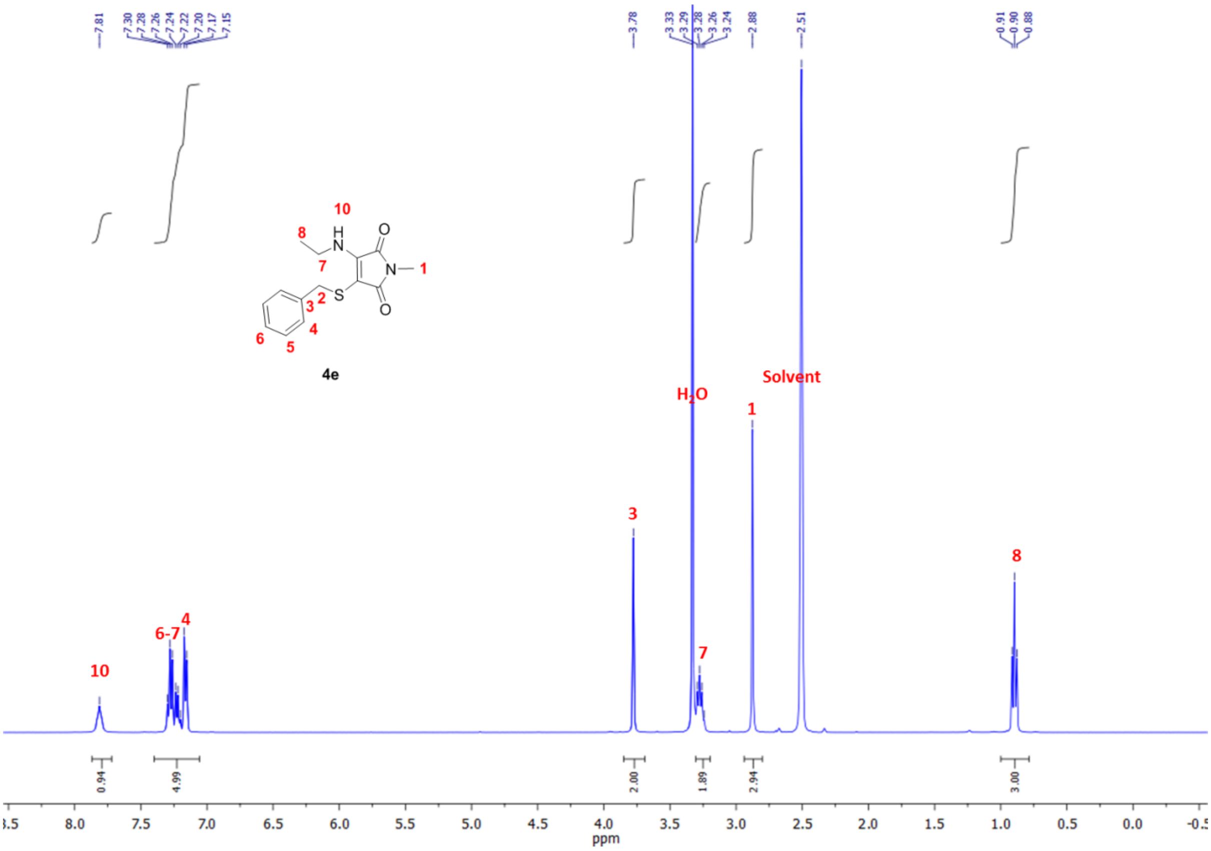

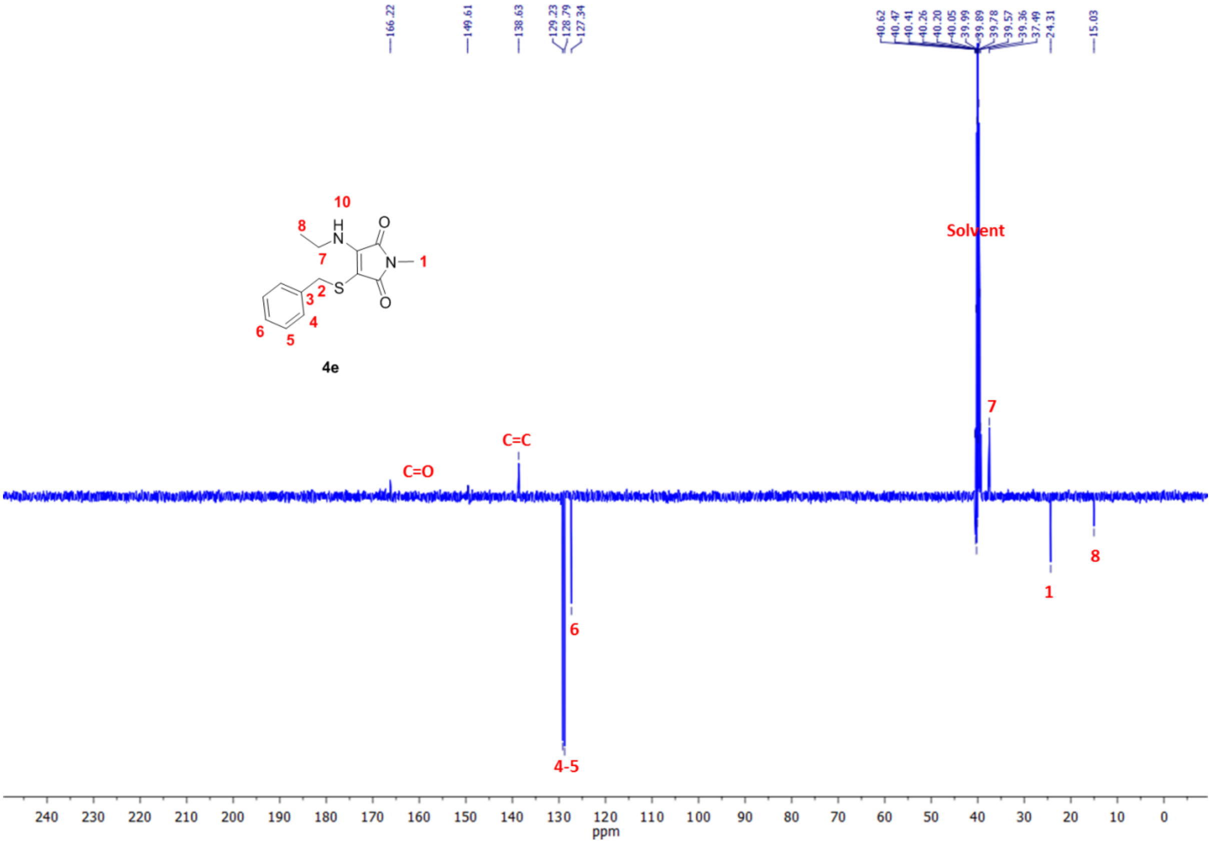

3-(butylamino)-1-methyl-4-(phenylthio)-1H-pyrrole-2,5-dione (4f):

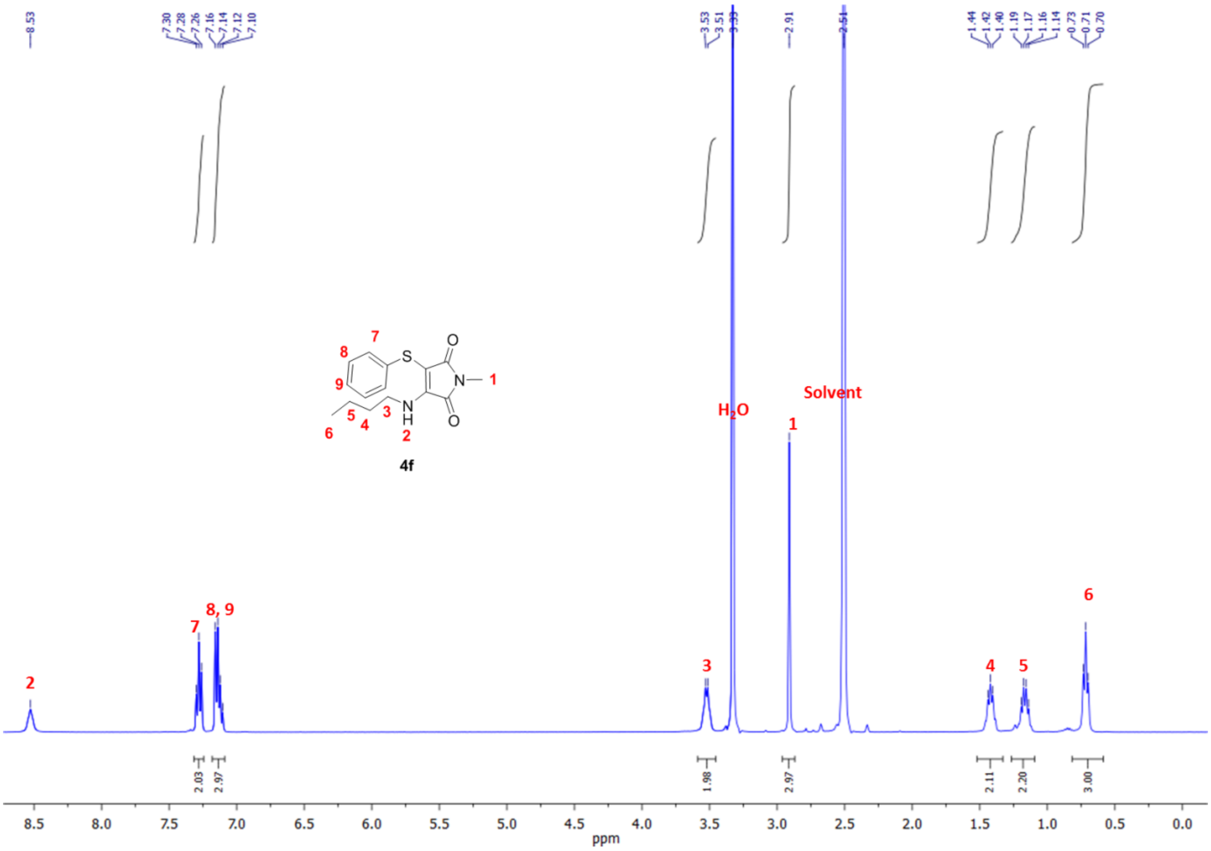

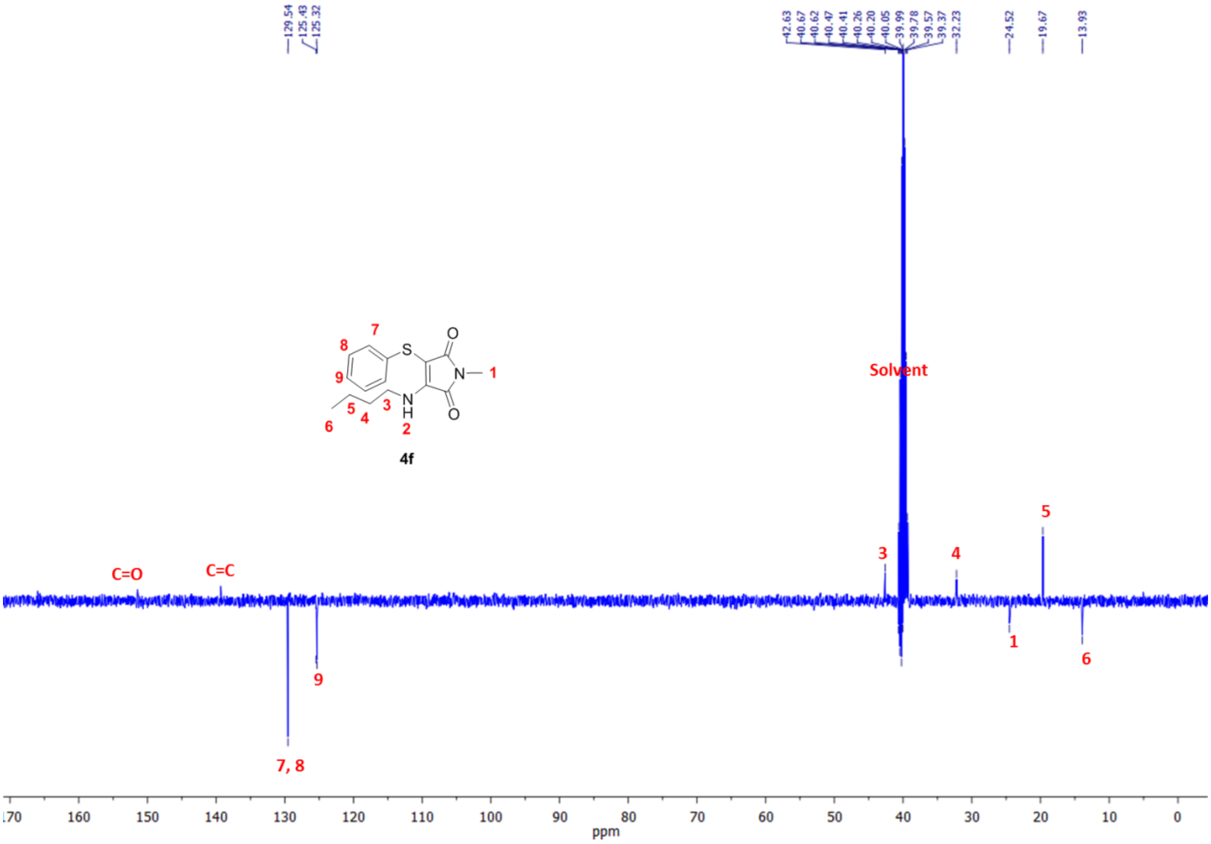

1-methyl-3-(phenylamino)-4-(phenylthio)-1H-pyrrole-2,5-dione (4g)

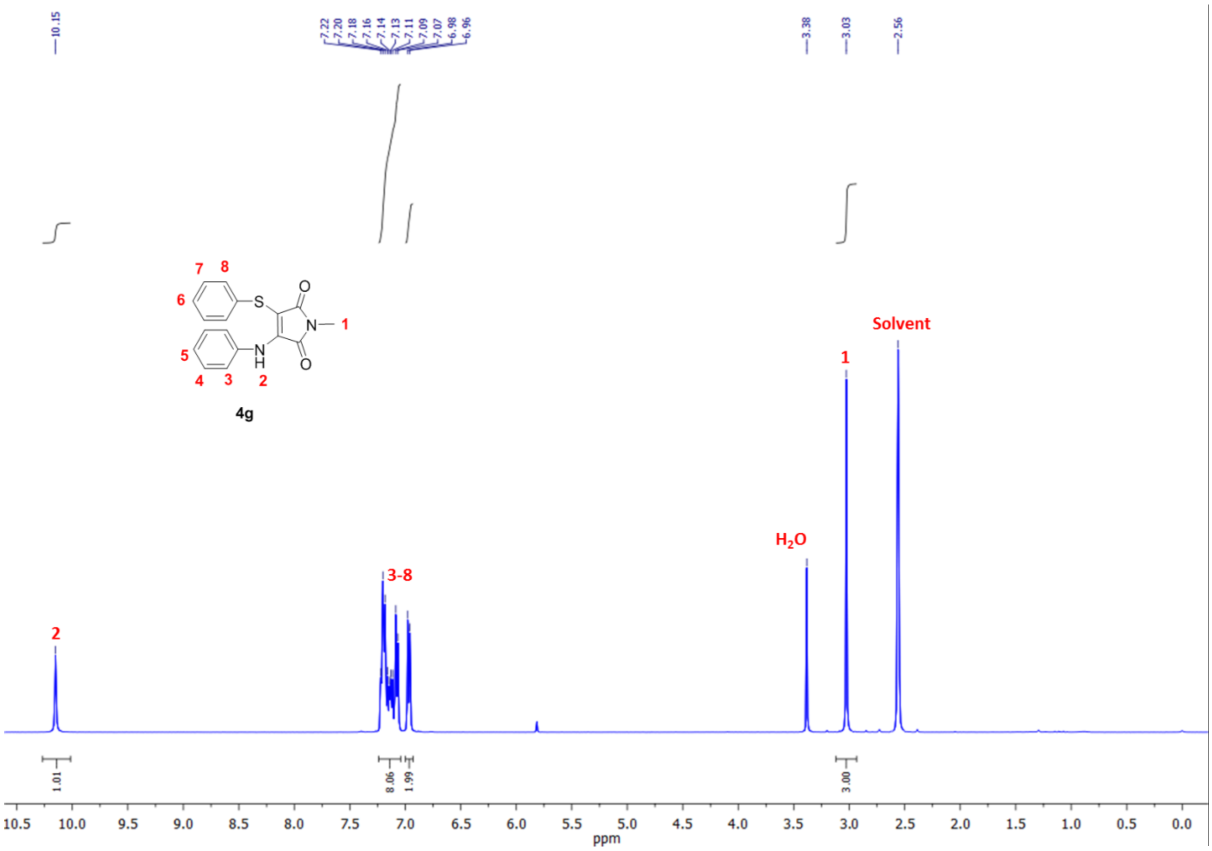

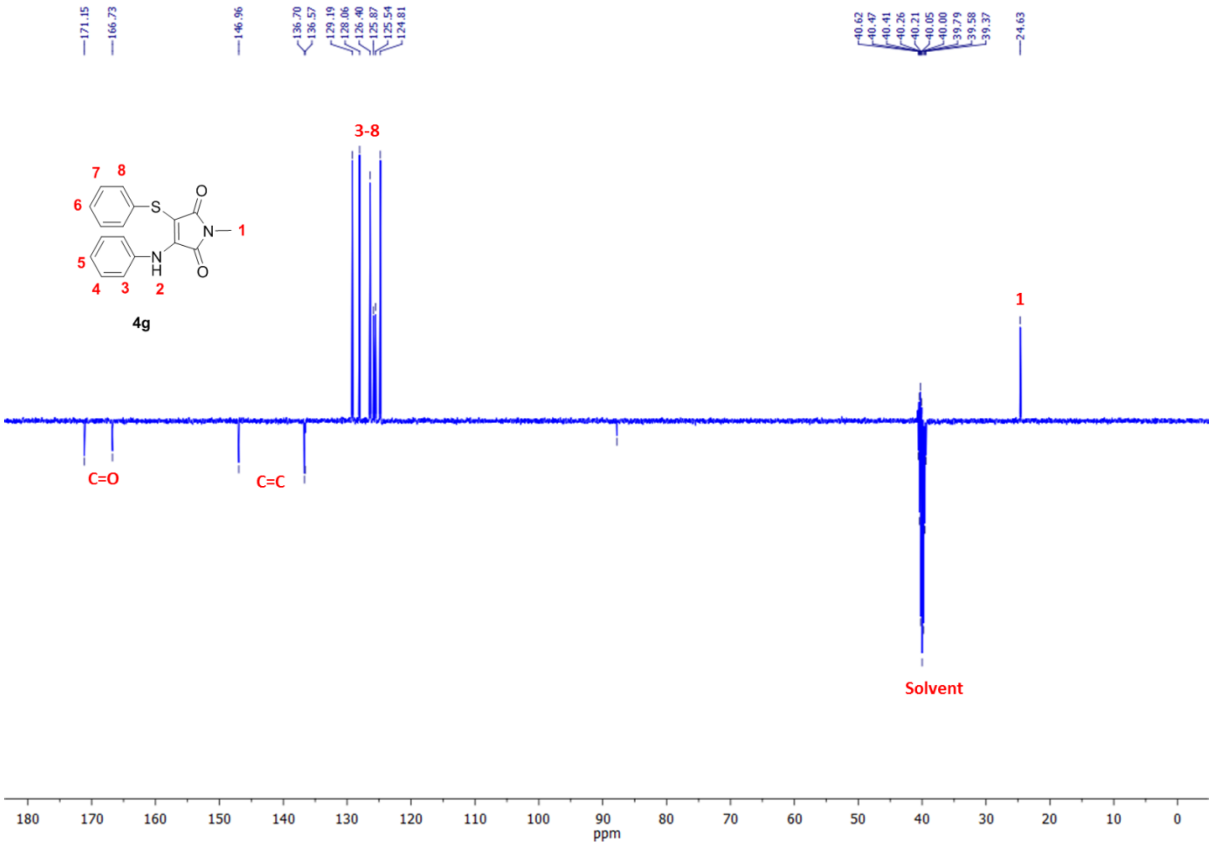

3-(butylamino)-4-(phenylthio)-1H-pyrrole-2,5-dione (4h)

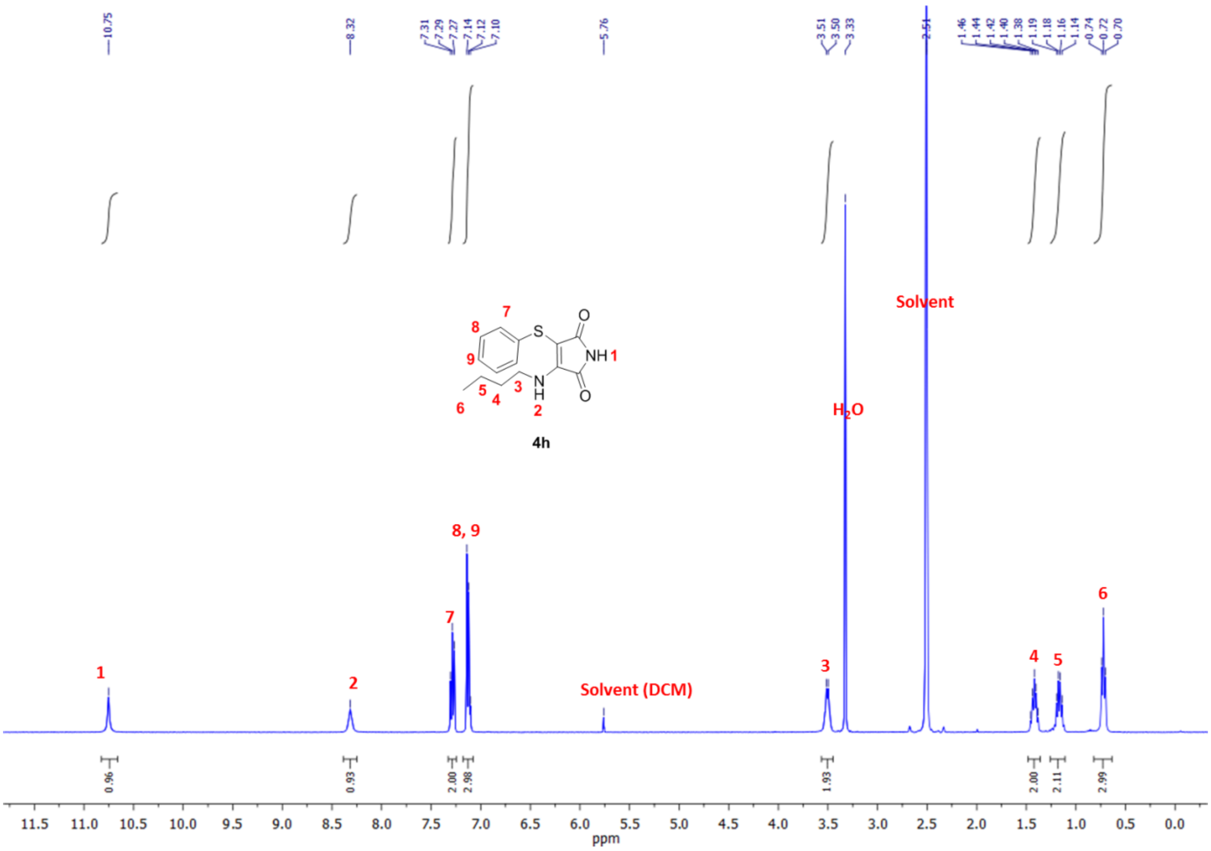

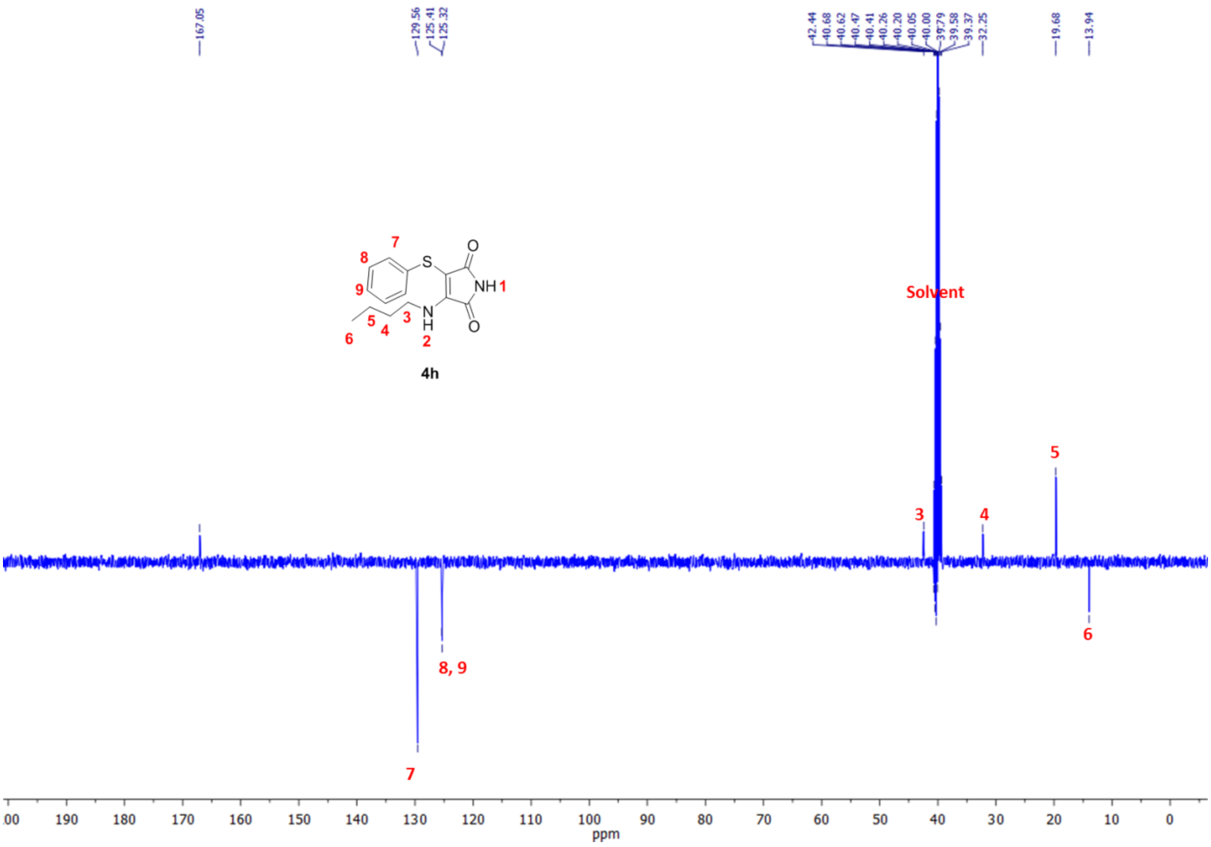

3-(butylthio)-4-(ethylamino)-1H-pyrrole-2,5-dione (4i)

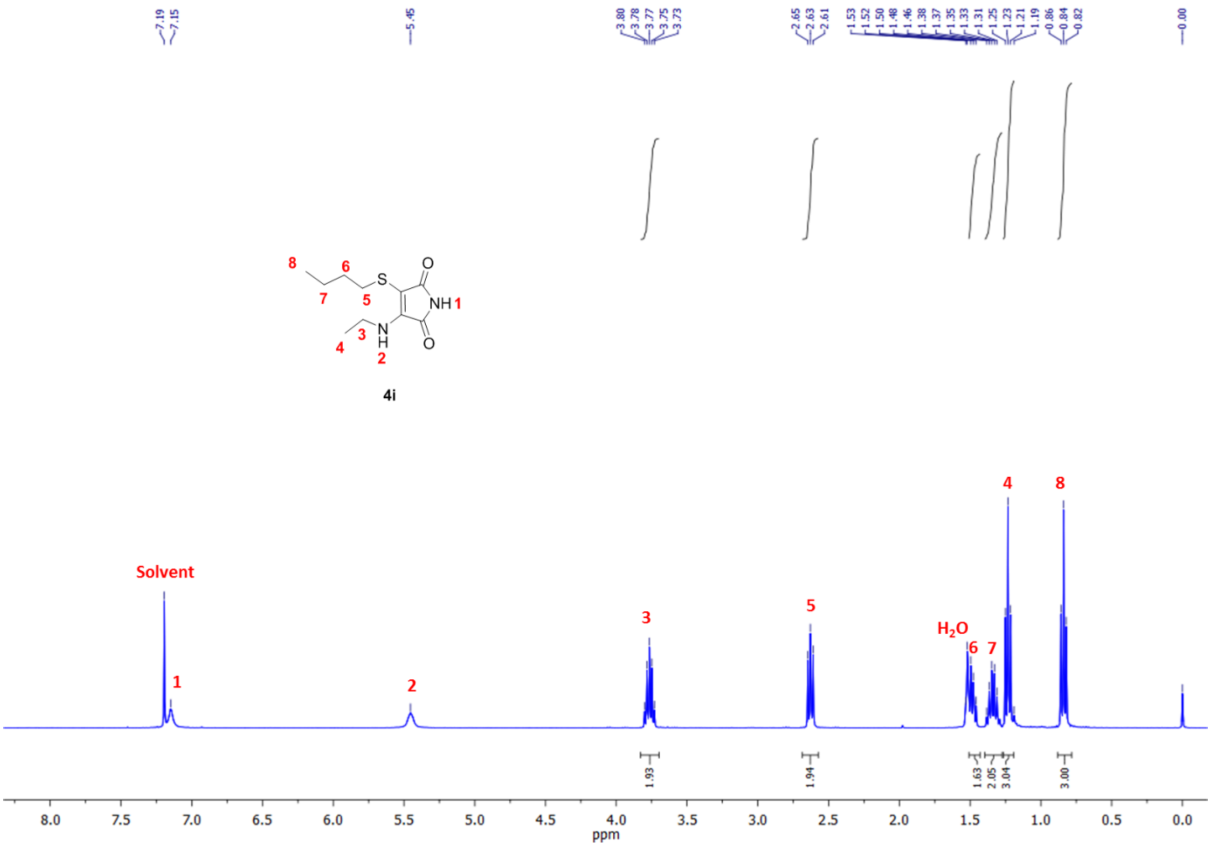

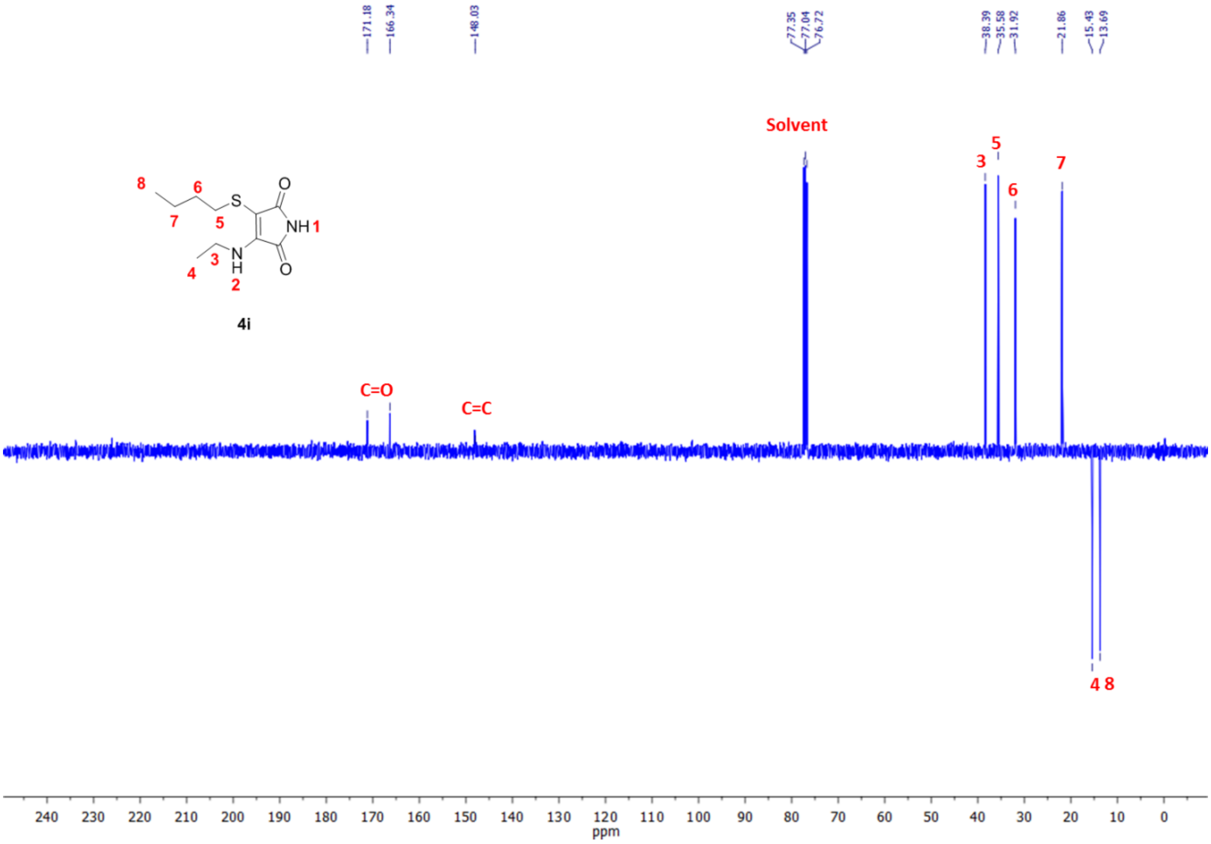

3-(benzylamino)-4-(butylthio)-1H-pyrrole-2,5-dione (4j)

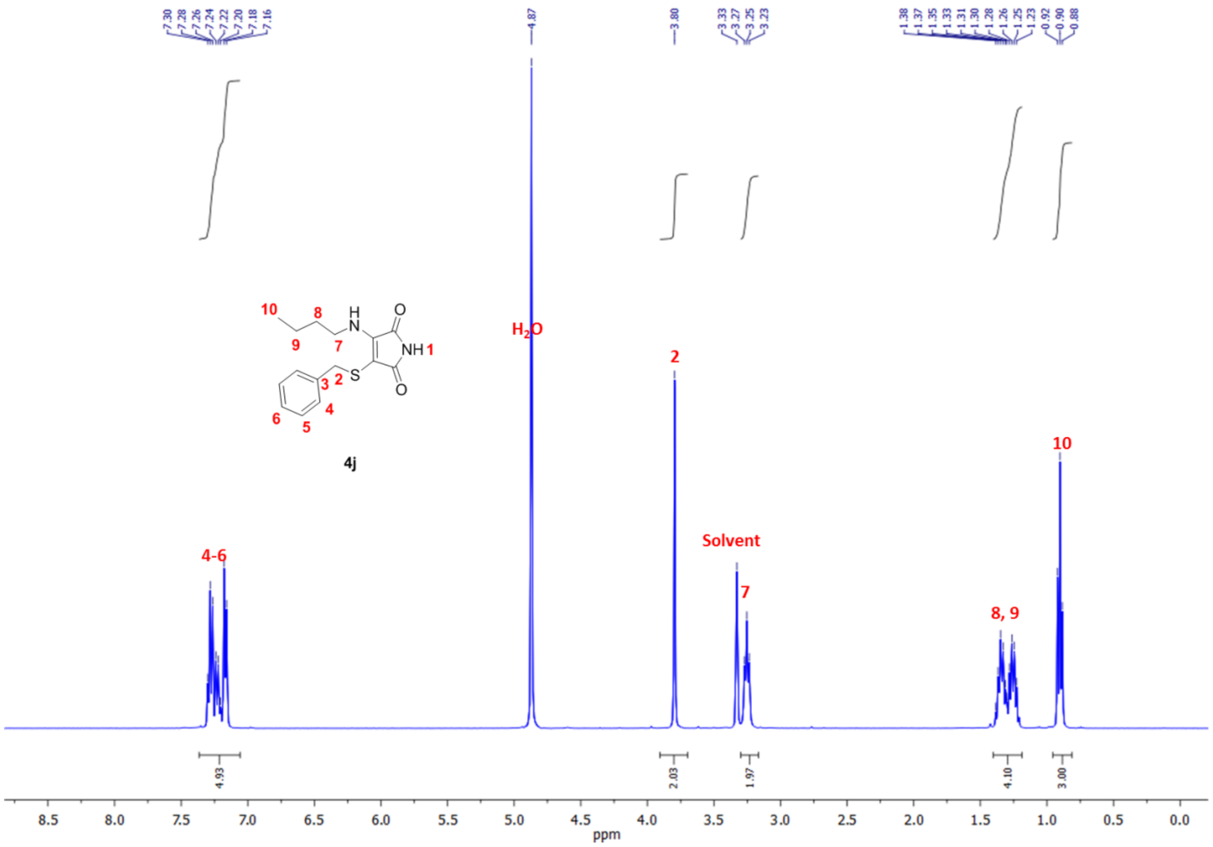

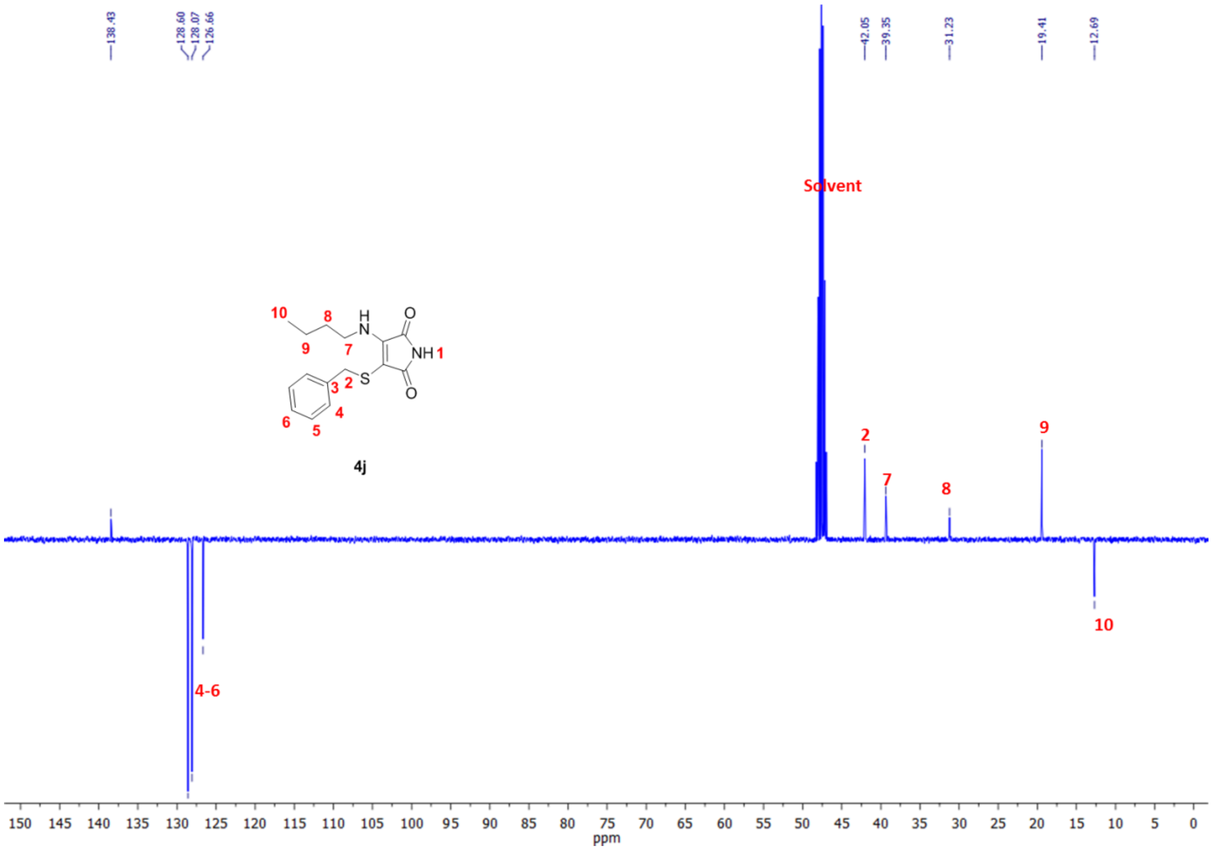

9. COMPUTATIONAL DETAILS

The geometries for all studied systems in the ground state have been fully optimized with the CAM-B3LYP[7] functional and the 6-311G(d,p) basis set.[8,9] The dispersion effects and the solvent were included in the optimization process. The D3-Grimme's dispersion[10] with Becke-Johson damping factor[11,12] was used to evaluate the dispersion effects. The solvent was considered using the polarization continuum model (PCM)[13, 14] and the dielectric constant of diethyl ether (ε = 4.24). The harmonic vibrational frequencies were also calculated at the same level of theory in order to verify that all the stationary points are minima of their potential energy surface. The time-dependent density functional theory (TD-DFT)[15-23] was applied to compute the absorption and emission (fluorescence) spectra at the ground and the first singlet excited state, respectively. The charge distribution was obtained following the natural bond orbital (NBO) partition scheme by Weinhold and co-workers.[24] All the calculations were performed using the Gaussian09 program package.[25]

Table S2. TD-DFT excitation and emission energies and oscillator strengths of the maleimide derivatives studied in this work.[a,b]

**Compound**	**λ_{exc}** [c]	**f_{exc}** [d]	**λ_{em}** [e]	**f_{em}** [d]	**$\Delta\lambda$** [f]
**1a**	302.4	0.023	468.4	0.011	166.0
**1b**	309.2	0.040	465.6	0.022	156.4
**1c**	323.2	0.078	463.7	0.058	140.5
**2a**	338.6	0.087	460.2	0.070	121.6
**2b**	339.0	0.087	458.9	0.073	119.9
**2c**	339.9	0.088	458.5	0.078	118.6
**2d**	330.3	0.121	435.3	0.104	104.9
**2e**	331.3	0.119	435.4	0.105	104.2
**3a**	307.3	0.035	442.7	0.028	135.4
**3b**	309.0	0.038	442.1	0.032	133.1
**3c**	308.0	0.031	449.7	0.022	141.7
**3d**	310.2	0.035	449.8	0.026	139.5
**3e**	306.3	0.033	445.5	0.024	139.2
**3f**	308.9	0.036	459.8	0.035	151
**4a**	334.0	0.090	551.5	0.084	217.5
**4b**	333.8	0.090	552.9	0.085	219.0
**4c**	328.3	0.067	510.4	0.065	182.1
**4d**	328.1	0.068	511.5	0.065	183.4
**4e**	336.2	0.095	550.6	0.089	214.5
**4f**	336.8	0.064	510.0	0.065	173.2
**4g**	373.5	0.088	508.2	0.098	134.7
**4h**	316.7	0.087	491.4	0.084	174.8
**4i**	327.2	0.115	528.8	0.106	201.5
**4j**	326.3	0.104	537.6	0.105	211.3

[a] All excitation and emission energies correspond to the first excited state ($\pi \rightarrow \pi^*$ transition). [b] All values computed at CAM-B3LYP-D3BJ/6-311G(d,p) level of theory in solution using the PCM with $\varepsilon = 4.24$. [c] Excitation wavelength, in nm. [d] Oscillator strength. [e] Emission wavelength, in nm. [f] Stokes shift ($\lambda_{em} - \lambda_{exc}$).

Table S3. Natural population analysis of the Carbon atoms adjacent to the electron withdrawing and donating groups (labeled as C_2 and C_3, respectively).[a]

Compound	q_{C2}	q_{C3}	Δq
**1a**	-0.13	-0.13	0.00
**1b**	-0.20	-0.20	0.00
**1c**	-0.32	-0.32	0.00
**2a**	-0.24	0.15	0.39
**2b**	-0.32	0.15	0.47
**2c**	-0.44	0.15	0.59
**2d**	-0.25	0.14	0.39
**2e**	-0.32	0.14	0.47
**3a**	-0.23	0.29	0.52
**3b**	-0.31	0.29	0.6
**3c**	-0.22	0.28	0.50
**3d**	-0.29	0.28	0.57
**3e**	-0.21	0.28	0.49
**3f**	-0.29	0.28	0.57
**4a**	-0.40	0.18	0.58
**4b**	-0.40	0.18	0.58
**4c**	-0.42	0.19	0.61
**4d**	-0.42	0.19	0.61
**4e**	-0.40	0.18	0.59
**4f**	-0.42	0.19	0.61
**4g**	-0.38	0.16	0.53
**4h**	-0.42	0.19	0.61
**4i**	-0.40	0.17	0.57
**4j**	-0.42	0.19	0.60

[a] All values computed at CAM-B3LYP-D3BJ/6-311G(d,p) level of theory in solution using the PCM with ε = 4.24.

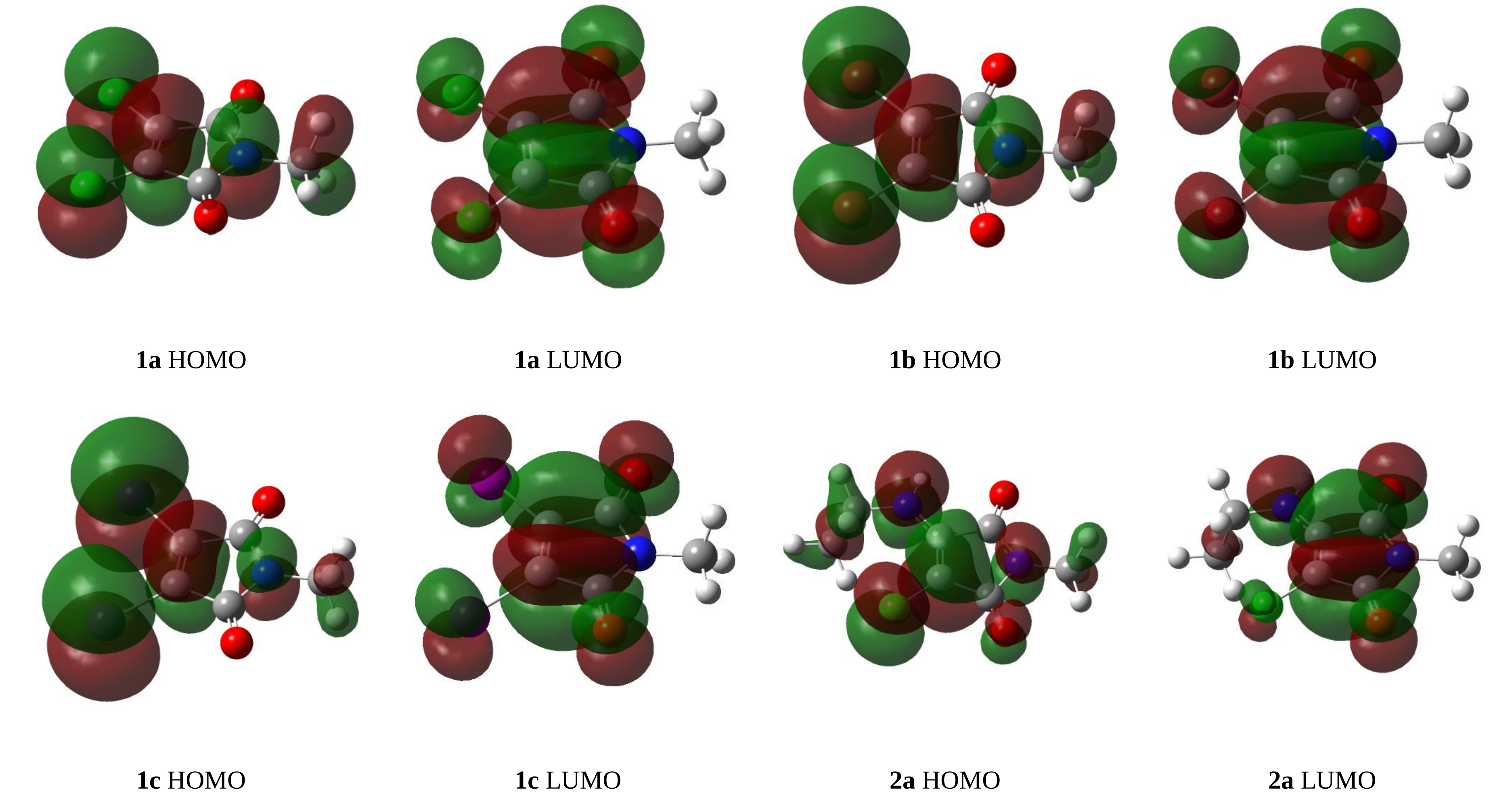

Figure S17. HOMO and LUMO Kohn-Sham orbitals of **1a**, **1b**, **1c** and **2a** maleimide derivatives.

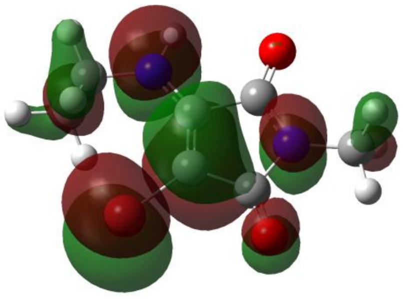

2b HOMO

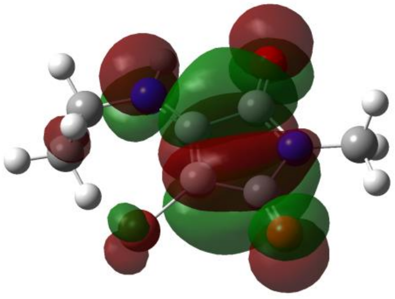

2b LUMO

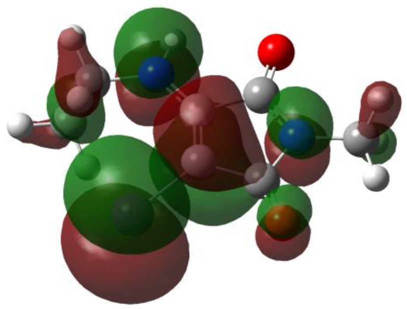

2c HOMO

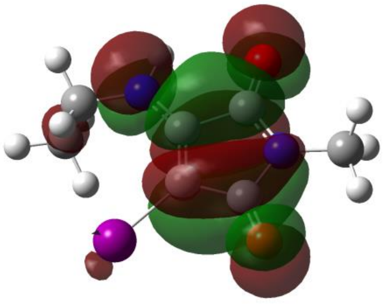

2c LUMO

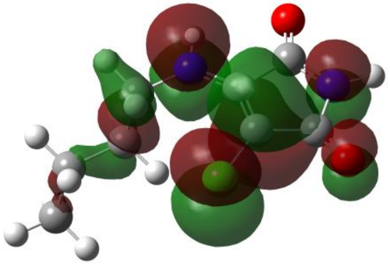

2d HOMO

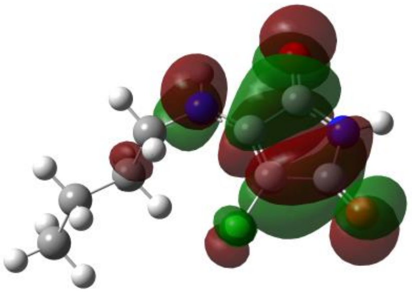

2d LUMO

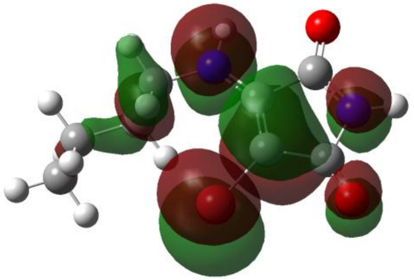

2e HOMO

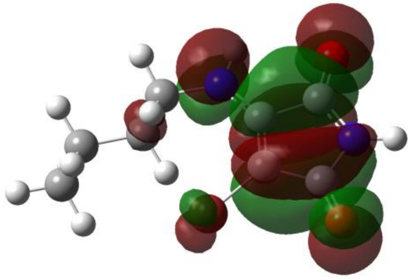

2e LUMO

Figure S18. HOMO and LUMO Kohn-Sham orbitals of **2b**, **2c**, **2d** and **2e** maleimide derivatives.

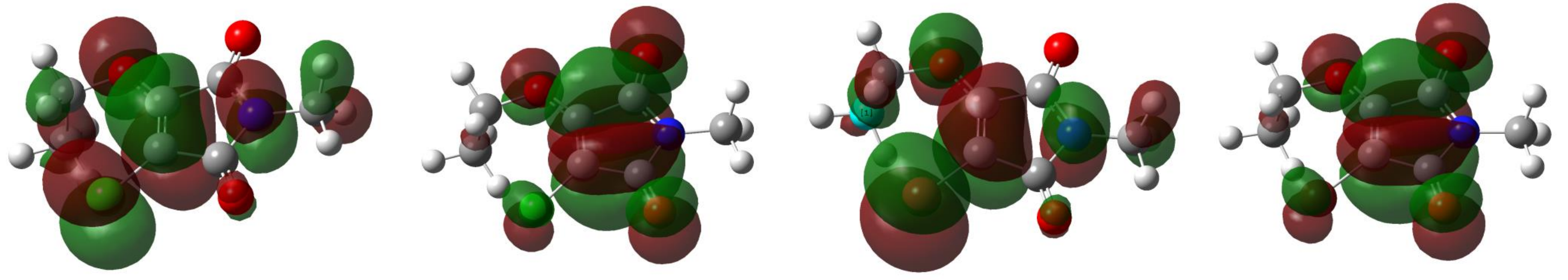

3a HOMO | **3a** LUMO | **3b** HOMO | **3b** LUMO

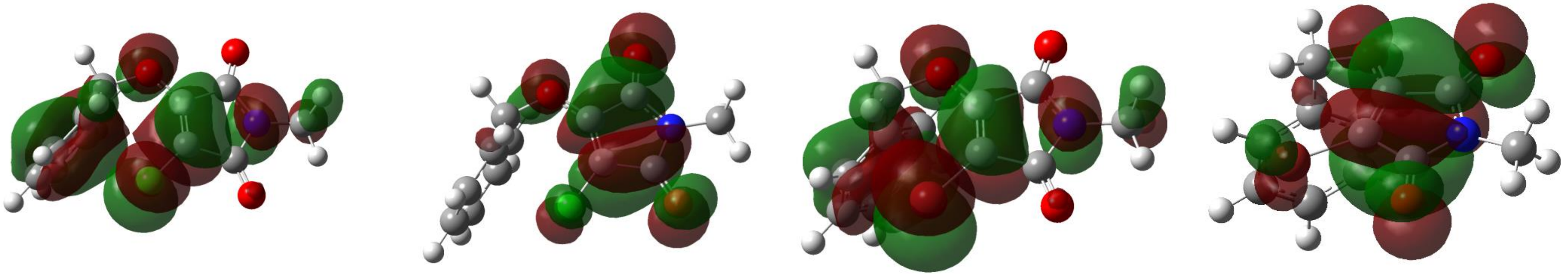

3d HOMO | **3d** LUMO | **3c** HOMO | **3c** LUMO

Figure S19. HOMO and LUMO Kohn-Sham orbitals of **3a**, **3b**, **3c** and **3d** maleimide derivatives.

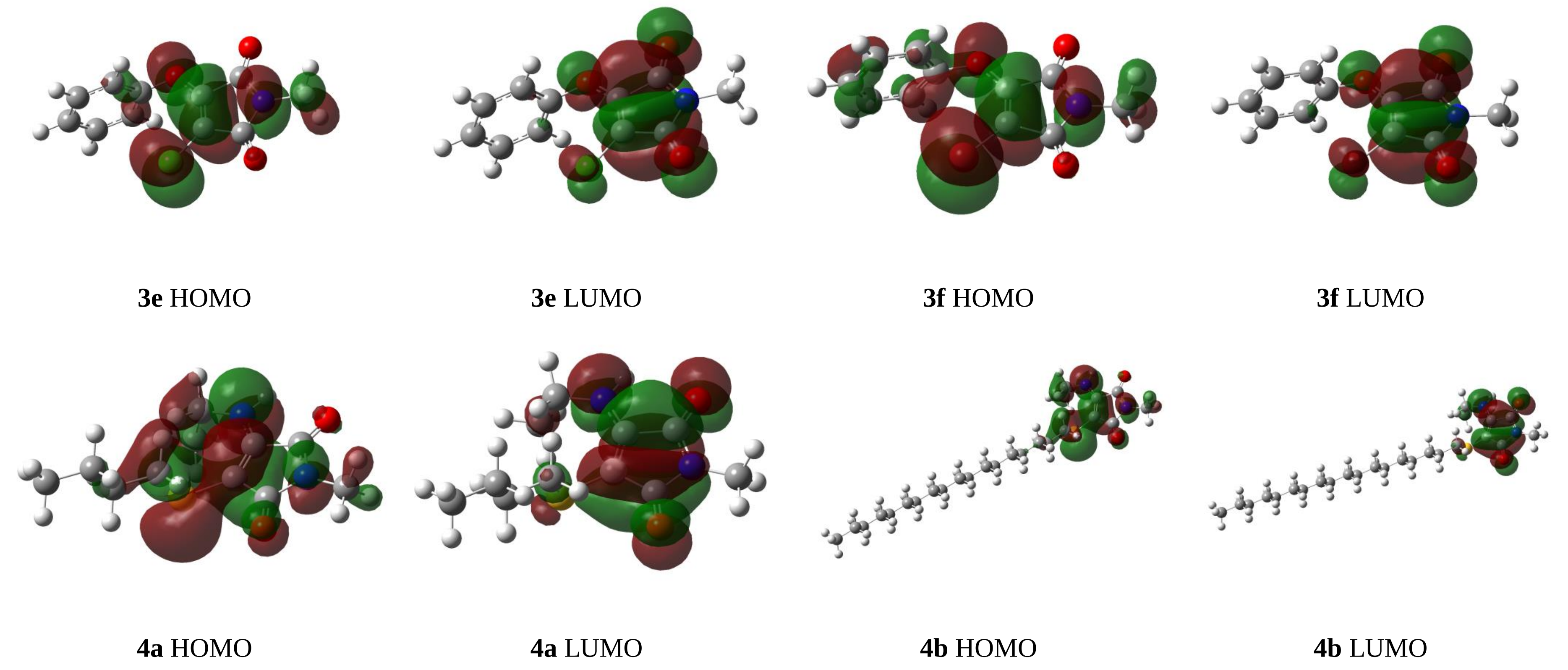

Figure S20. HOMO and LUMO Kohn-Sham orbitals of **3e**, **3f**, **4a**, and **4b** maleimide derivatives.

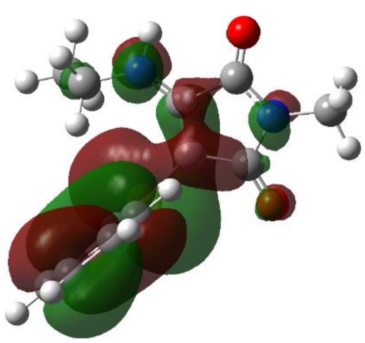

4c HOMO

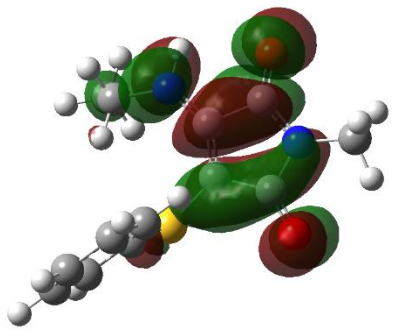

4c LUMO

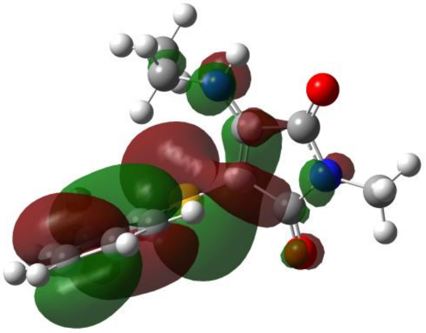

4d HOMO

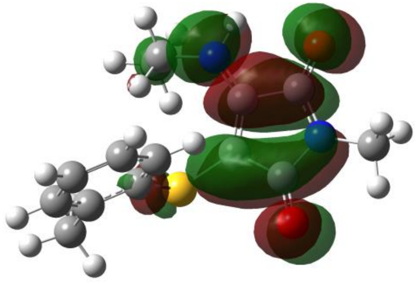

4d LUMO

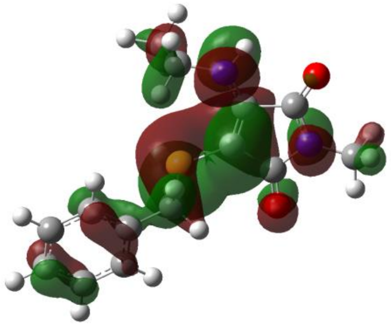

4e HOMO

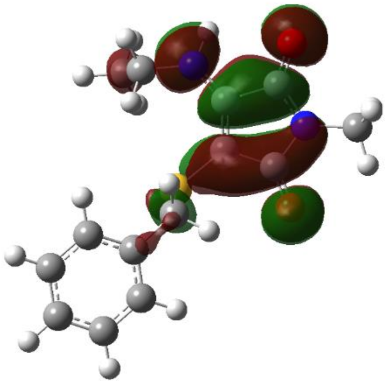

4e LUMO

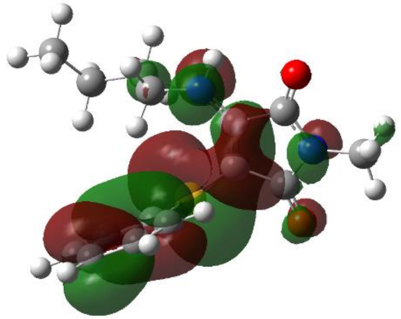

4f HOMO

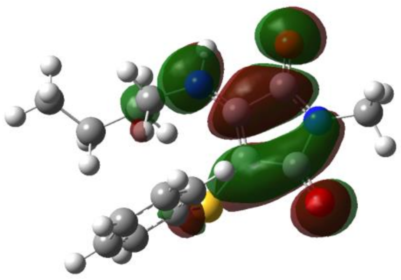

4f LUMO

Figure S21. HOMO and LUMO Kohn-Sham orbitals of **4c**, **4d**, **4e** and **4f** maleimide derivatives.

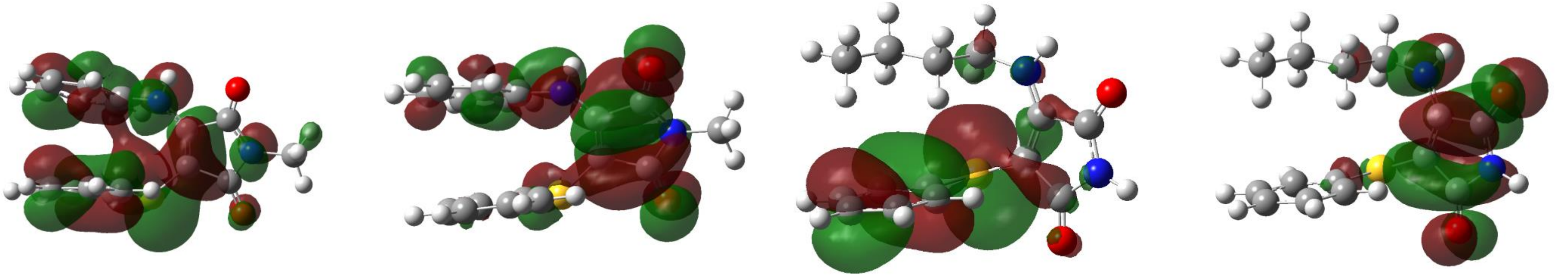

**4g** HOMO	**4g** LUMO	**4h** HOMO	**4h** LUMO

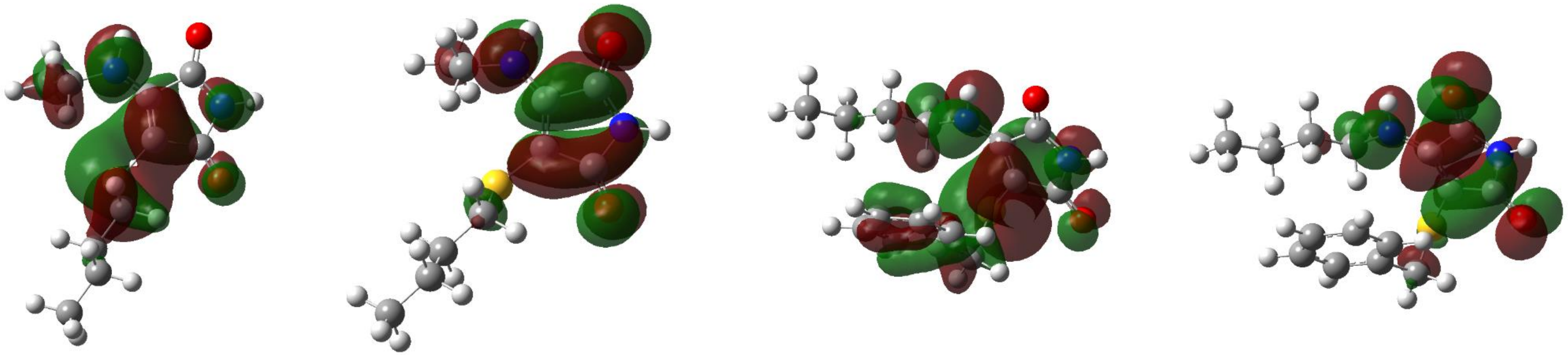

**4i** HOMO	**4i** LUMO	**4j** HOMO	**4j** LUMO

Figure S22. HOMO and LUMO Kohn-Sham orbitals of **4g**, **4h**, **4i** and **4j** maleimide derivatives.

Table S4. Cartesian coordinates (in Å) of the stationary points in the ground state optimized at the CAM-B3LYP-D3BJ/6-311G(d,p) level of theory in solution using the PCM with ε = 4.24.

1a

C	0.864909	1.139077	-0.001752
C	-0.569939	0.677136	-0.000135
C	-0.586832	-0.650148	0.002315
C	0.836264	-1.146833	0.001007
N	1.631825	-0.014532	-0.001932
O	1.265302	2.267340	-0.004657
O	1.214277	-2.282938	0.002625
C	3.082796	-0.039832	-0.001653
H	3.452921	-0.529994	0.898060
H	3.450648	-0.568592	-0.879992
H	3.427360	0.991100	-0.024105
Cl	-1.861360	1.776889	-0.000987
Cl	-1.905628	-1.716690	0.005352

1b

C	-1.425260	-1.090979	0.114405
C	-0.069890	-0.456563	-0.079915
C	-0.231534	0.856101	-0.203217
C	-1.704211	1.168563	-0.098332
N	-2.336708	-0.047679	0.089115
O	-1.673575	-2.253254	0.260474
O	-2.233835	2.240925	-0.161487
C	-3.771339	-0.203314	0.241265
H	-4.120668	0.345845	1.114815
H	-4.287323	0.161564	-0.646027
H	-3.972711	-1.263790	0.371304
Br	1.474886	-1.476326	-0.117349
Br	1.023108	2.192326	-0.461961

1c

C	-1.346667	-1.340713	0.135454
C	-0.256196	-0.312673	-0.061393
C	-0.824711	0.881465	-0.204375
C	-2.323235	0.709406	-0.110805
N	-2.540771	-0.641430	0.090799
O	-1.219077	-2.521045	0.296806
O	-3.167947	1.555468	-0.191632
C	-3.854055	-1.240512	0.236768
H	-4.370715	-0.812824	1.095157
H	-4.447251	-1.074885	-0.661834
H	-3.711425	-2.307473	0.389530
I	1.731796	-0.871452	-0.074456
I	0.008964	2.745927	-0.507641

2a

C	1.476204	-0.119859	-0.842751
C	2.851062	0.283966	-1.150410
C	3.251094	1.222803	-0.262051
C	2.086949	1.455194	0.686551
N	1.076971	0.638168	0.283784
O	0.773511	-0.925263	-1.393003
O	2.096949	2.225381	1.614741
C	-0.220254	0.539015	0.918006
H	-0.116698	0.219920	1.955216
H	-0.736506	1.498574	0.886608
H	-0.794244	-0.200975	0.365200
N	4.342220	1.948013	-0.043250
H	4.293207	2.534341	0.779516
C	5.620905	1.810330	-0.727777
H	6.157647	2.747947	-0.583891
C	6.443295	0.636477	-0.212929
H	5.917896	-0.306008	-0.374418
H	7.397998	0.588265	-0.739966
H	6.644130	0.742228	0.854720
H	5.433594	1.709206	-1.796206
Cl	3.695361	-0.421379	-2.478955

2b

C	1.457789	-0.113997	-0.851235
C	2.835552	0.276277	-1.165084
C	3.247637	1.206088	-0.270407
C	2.089608	1.443871	0.687368
N	1.071550	0.639400	0.284636
O	0.739171	-0.905930	-1.400741
O	2.112819	2.208693	1.620030
C	-0.223704	0.548011	0.924008
H	-0.117931	0.223520	1.959324
H	-0.732366	1.511821	0.899069
H	-0.805920	-0.185039	0.370639
Br	3.726674	-0.500921	-2.622747
N	4.340114	1.925306	-0.043360
H	4.286588	2.505844	0.783711
C	5.621203	1.805842	-0.726079
H	6.138471	2.756119	-0.593517
C	6.467062	0.655975	-0.196022
H	5.961917	-0.299080	-0.347576
H	7.423747	0.622272	-0.720624
H	6.663491	0.778386	0.870637
H	5.435658	1.686864	-1.792589

2c

C	1.452543	-0.113316	-0.860887
C	2.830188	0.270521	-1.189197
C	3.247112	1.197407	-0.289756
C	2.095502	1.438110	0.677860
N	1.074339	0.637817	0.279536
O	0.725156	-0.902624	-1.403678
O	2.127923	2.201056	1.612006
C	-0.216884	0.547623	0.927400
H	-0.103834	0.224402	1.962312
H	-0.724783	1.511912	0.905062
H	-0.803462	-0.185426	0.378783
N	4.337877	1.915605	-0.055338

H	4.275627	2.493346	0.773649
C	5.626979	1.804792	-0.723524
H	6.134313	2.760366	-0.591082
C	6.477259	0.666414	-0.175956
H	5.981348	-0.294323	-0.321951
H	7.438264	0.635314	-0.692725
H	6.664094	0.800616	0.891030
H	5.455482	1.678873	-1.791404
I	3.773069	-0.598993	-2.829541

2d

C	2.479933	1.044477	0.177009
C	1.124593	0.741599	-0.293395
C	0.964167	-0.600342	-0.379413
C	2.274614	-1.234709	0.057502
N	3.112162	-0.204309	0.362385
O	3.001648	2.106693	0.382105
O	2.480181	-2.419472	0.110861
N	-0.002132	-1.436215	-0.742534
H	0.226630	-2.414529	-0.627079
C	-1.371660	-1.069815	-1.071916
H	-1.796776	-1.898630	-1.639384
H	-1.352771	-0.204349	-1.735001
C	-2.224488	-0.775602	0.157924
C	-3.641133	-0.355302	-0.215998
H	-1.748653	0.018380	0.741020
H	-2.252285	-1.664748	0.795895
C	-4.504155	-0.054475	1.003773
H	-3.594313	0.529533	-0.859340
H	-4.109261	-1.145384	-0.812676
H	-4.591076	-0.932175	1.649281
H	-4.071436	0.753742	1.598692
Cl	0.017169	2.015862	-0.646073
H	4.060692	-0.308061	0.689122
H	-5.512192	0.247498	0.713290

2e

C	2.517878	0.720056	0.348404
C	1.156747	0.486679	-0.145756
C	0.986856	-0.836751	-0.385464
C	2.296517	-1.528409	-0.034671
N	3.142177	-0.546984	0.382664
O	3.053691	1.744453	0.674777
O	2.492302	-2.712950	-0.122456
Br	-0.027332	1.928070	-0.348928
N	0.019476	-1.627154	-0.834873
H	0.254754	-2.611002	-0.835038
C	-1.352576	-1.236618	-1.122020
H	-1.766286	-1.991220	-1.792281
H	-1.340439	-0.293678	-1.669086
C	-2.212185	-1.110494	0.131328
C	-3.629831	-0.653098	-0.191685
H	-1.743517	-0.396235	0.814545
H	-2.236999	-2.075111	0.648407
C	-4.499950	-0.518963	1.052442
H	-3.585262	0.308424	-0.713658
H	-4.090477	-1.360844	-0.889045
H	-4.582188	-1.473436	1.578635
H	-4.075971	0.208653	1.749001
H	4.092045	-0.693773	0.688479
H	-5.509264	-0.189529	0.798581

3a

C	1.336298	0.936175	0.083577
C	-0.122224	0.719931	-0.116697
C	-0.352667	-0.597591	-0.223826
C	0.985549	-1.306789	-0.094497
N	1.930601	-0.325753	0.082819
O	1.916012	1.978086	0.220513
O	1.173247	-2.490889	-0.137747
C	3.348892	-0.568208	0.251788
H	3.530727	-1.161357	1.147840
H	3.750379	-1.092662	-0.614907
H	3.835201	0.399251	0.350477
C	-2.729806	-0.849664	-0.426826
H	-3.308873	-1.632546	-0.910678
C	-3.226860	-0.585500	0.975791
H	-2.644277	0.192721	1.469451
H	-4.265852	-0.253619	0.933742
H	-3.178618	-1.496049	1.574035
H	-2.754776	0.040377	-1.053517
Cl	-1.169079	2.081193	-0.181622
O	-1.384382	-1.384152	-0.410637

3b

C	-1.301024	-0.990615	0.122374
C	0.035125	-0.367760	-0.083377
C	-0.128900	0.957100	-0.227016
C	-1.618450	1.251019	-0.120870
N	-2.237778	0.043663	0.083512
O	-1.559468	-2.150496	0.291537
O	-2.141350	2.327879	-0.200590
C	-3.667158	-0.132985	0.240542
H	-4.026527	0.421897	1.106927
H	-4.192813	0.210359	-0.650205
H	-3.850501	-1.194836	0.385739
Br	1.544969	-1.475186	-0.115199
C	2.067251	1.914240	-0.421325
H	2.386653	2.815236	-0.939631
C	2.594799	1.877337	0.994312
H	2.269605	0.979601	1.520667
H	3.686160	1.879167	0.972817
H	2.257971	2.753984	1.548694
H	2.375103	1.047748	-1.003688
O	0.623082	2.009873	-0.434222

3c

C	1.224140	0.906986	0.180438
C	-0.219019	0.717015	-0.142358
C	-0.449233	-0.589927	-0.320392
C	0.860902	-1.322710	-0.102120
N	1.802239	-0.361496	0.179229
O	1.799957	1.936213	0.400013
O	1.033244	-2.509197	-0.153253
C	3.197724	-0.631372	0.460796
H	3.289675	-1.294206	1.320777
H	3.679232	-1.091137	-0.402007
H	3.676005	0.320118	0.680259
C	-2.817434	-0.826255	-0.525748
H	-3.427169	-1.609020	-0.974339

H	-2.909395	0.073399	-1.132820
Cl	-1.242785	2.088027	-0.255538
O	-1.479427	-1.352643	-0.619863
C	-3.220109	-0.574046	0.902332
C	-4.086527	0.469665	1.204180
C	-2.761859	-1.399877	1.923947
C	-4.499331	0.682434	2.511558
H	-4.434112	1.126033	0.414555
C	-3.164509	-1.179422	3.232452
H	-2.083046	-2.212065	1.693156
C	-4.036278	-0.140025	3.528836
H	-5.173481	1.499666	2.736907
H	-2.798139	-1.823018	4.022907
H	-4.350178	0.031082	4.551261

3d

C	-1.153598	-0.951408	0.170379
C	0.159434	-0.325914	-0.161221
C	-0.028618	0.989734	-0.329453
C	-1.499566	1.283545	-0.091039
N	-2.095409	0.077628	0.185607
O	-1.387740	-2.108403	0.384991
O	-2.027509	2.360817	-0.124035
C	-3.502405	-0.097937	0.483682
H	-3.779804	0.481189	1.364205
H	-4.112897	0.217374	-0.362126
H	-3.665379	-1.155545	0.676216
Br	1.667688	-1.418495	-0.314419
C	2.140032	1.984693	-0.520845
H	2.463288	2.916194	-0.983118
H	2.531944	1.154052	-1.105930
O	0.705064	2.039113	-0.633534
C	2.583434	1.908169	0.915599
C	3.752493	1.232547	1.244637
C	1.854116	2.540734	1.917303
C	4.193994	1.194814	2.559330
H	4.317219	0.725416	0.470750
C	2.289014	2.492538	3.233275
H	0.940925	3.065998	1.664986
C	3.460944	1.821926	3.556994
H	5.105440	0.664222	2.806093
H	1.711639	2.982436	4.007807
H	3.799667	1.784923	4.585121

3e

C	-2.170679	-0.716178	0.769833
C	-0.801335	-0.499213	0.215351
C	-0.752946	0.728893	-0.312095
C	-2.107125	1.379239	-0.123620
N	-2.889117	0.451221	0.527217
O	-2.598698	-1.691744	1.320880
O	-2.424013	2.481395	-0.474692
Cl	0.374637	-1.737863	0.331821
O	0.156969	1.465771	-0.927221
C	-4.271449	0.652286	0.913510
H	-4.908284	-0.098707	0.447372
H	-4.377486	0.592188	1.996301
H	-4.565293	1.642423	0.573118
C	1.436552	0.934693	-1.141604
C	1.686344	0.258829	-2.319443
C	2.412347	1.148534	-0.188357
C	2.962418	-0.239870	-2.538076
H	0.892126	0.124471	-3.042279
C	3.684247	0.644762	-0.419165
H	2.172176	1.693302	0.715459
C	3.958420	-0.051668	-1.588901
H	3.176983	-0.777451	-3.452988
H	4.461833	0.797175	0.318550
H	4.952360	-0.443589	-1.763887

3f

C	-2.303185	0.864033	-0.550841
C	-0.827052	0.648519	-0.482922
C	-0.607356	-0.559947	0.048179
C	-1.948208	-1.196453	0.355819
N	-2.900682	-0.285573	-0.033018
O	-2.890750	1.824298	-0.964636
O	-2.134541	-2.276758	0.842847
O	0.450141	-1.294915	0.348270
C	-4.330647	-0.488572	0.084268
H	-4.818979	0.400082	-0.308142
H	-4.639373	-1.360008	-0.492578
H	-4.611216	-0.628972	1.127879
C	1.734780	-0.753711	0.207144
C	2.454823	-1.049463	-0.932869
C	2.253802	0.009848	1.234439
C	3.738442	-0.537280	-1.055832
H	2.009633	-1.661490	-1.706347
C	3.537748	0.516765	1.098639
H	1.657150	0.205166	2.116094
C	4.277326	0.248573	-0.045814
H	4.317112	-0.752318	-1.945220
H	3.959739	1.123238	1.889966
H	5.278652	0.647507	-0.147727
Br	0.325387	1.974869	-1.112633

4a

C	-1.442798	-1.076303	0.302654
C	-0.077374	-0.569172	0.099866
C	-0.173501	0.757198	-0.200204
C	-1.643625	1.141488	-0.180641
N	-2.334580	0.012548	0.117387
O	-1.817422	-2.190826	0.563696
O	-2.076629	2.248221	-0.398170
C	-3.773994	-0.079141	0.226901
H	-4.139978	0.556863	1.033384
H	-4.249178	0.218936	-0.707967
H	-4.015506	-1.116922	0.444634
N	0.665465	1.738044	-0.501350
H	0.222936	2.639977	-0.624232
C	2.119759	1.667753	-0.523514
H	2.464435	2.444854	-1.206207
C	2.737359	1.851563	0.855553
H	2.412858	1.059721	1.531855
H	3.826136	1.813515	0.786105
H	2.453034	2.814376	1.284200
H	2.410452	0.705931	-0.943203
S	1.331067	-1.595161	0.289873
C	1.337057	-2.486397	-1.309668
C	2.490427	-3.476023	-1.351175

H	1.423688	-1.752720	-2.112814
H	0.381747	-3.002479	-1.408667
C	2.536114	-4.253154	-2.664192
H	3.437060	-2.943608	-1.210439
H	2.398465	-4.178029	-0.516255
C	3.686468	-5.251974	-2.713123
H	1.586029	-4.778680	-2.803875
H	2.624293	-3.548738	-3.497841
H	3.700712	-5.796641	-3.659224
H	4.649784	-4.747044	-2.603742
H	3.603135	-5.985153	-1.906841

4b

C	8.350213	-1.559488	-0.499973
C	7.916037	-0.165981	-0.319063
C	8.747881	0.406317	0.596997
C	9.768779	-0.634705	1.024822
N	9.472648	-1.769828	0.343424
O	7.887919	-2.423602	-1.200068
O	10.656787	-0.451348	1.823299
C	10.189464	-3.021005	0.457478
H	11.231450	-2.894721	0.162519
H	10.149851	-3.394927	1.480936
H	9.706365	-3.731606	-0.209096
N	8.865805	1.589725	1.182210
H	9.662179	1.667859	1.801971
C	8.077715	2.783139	0.908121
H	8.116682	3.401650	1.805175
C	8.585057	3.557377	-0.300266
H	8.524282	2.947021	-1.202175
H	7.977402	4.451175	-0.453703
H	9.622194	3.866288	-0.157352
H	7.041652	2.483770	0.757523
S	6.578544	0.515751	-1.223715
C	5.147235	-0.128244	-0.280401
C	3.850167	0.384314	-0.885699
H	5.247325	0.194878	0.756936
H	5.185292	-1.217421	-0.312967
C	2.623060	-0.151162	-0.153807
H	3.842198	1.478963	-0.860857
H	3.799725	0.094202	-1.940019
C	1.311585	0.366576	-0.733724
H	2.629581	-1.246175	-0.191006
H	2.683490	0.121448	0.905990
H	1.300549	1.461505	-0.682014
H	1.260984	0.110335	-1.798195
C	0.079698	-0.184701	-0.024677
C	-1.231937	0.345500	-0.592818
H	0.136189	0.058629	1.042859
H	0.085823	-1.279057	-0.088888
C	-2.465953	-0.222856	0.098976
H	-1.244083	1.439010	-0.514411
H	-1.280785	0.116354	-1.663804
C	-3.776838	0.316975	-0.461819
H	-2.457795	-1.315668	0.011214
H	-2.413691	-0.002960	1.171839
H	-3.790008	1.408828	-0.362838
H	-3.823085	0.108187	-1.537132
C	-5.012213	-0.264902	0.216188
C	-6.322331	0.281666	-0.339884
H	-4.963848	-0.062652	1.292698
H	-5.001850	-1.356137	0.110544
C	-7.558724	-0.309984	0.327735
H	-6.336439	1.372067	-0.226090
H	-6.366223	0.087525	-1.418055
C	-8.868206	0.240812	-0.225660
H	-7.546339	-1.399937	0.209690
H	-7.513664	-0.119975	1.406614
H	-8.883103	1.330149	-0.102226
H	-8.910215	0.056195	-1.305582
C	-10.105321	-0.357278	0.434839
C	-11.414322	0.196096	-0.117142
H	-10.062700	-0.175166	1.515173
H	-10.091452	-1.446319	0.308810
C	-12.651958	-0.405766	0.539048
H	-11.429669	1.284747	0.012069
H	-11.455089	0.017193	-1.198067
C	-13.960622	0.148881	-0.012201
H	-12.637077	-1.494271	0.408482
H	-12.610826	-0.228084	1.620186
H	-13.976832	1.237365	0.119503
H	-14.001738	-0.027332	-1.093704
C	-15.199566	-0.453997	0.641720
H	-15.182586	-1.541225	0.509227
H	-15.157246	-0.277982	1.722073
C	-16.501848	0.108321	0.083235
H	-17.372803	-0.339110	0.566907
H	-16.557389	1.190131	0.231637
H	-16.582511	-0.081873	-0.990404

4c

C	-1.642071	-1.115150	0.689053
C	-0.295102	-0.719095	0.252710
C	-0.374019	0.556030	-0.229137
C	-1.814840	1.021472	-0.095977
N	-2.504086	-0.011230	0.449044
O	-2.025718	-2.151753	1.163730
O	-2.225264	2.109402	-0.422815
C	-3.915880	0.006043	0.766148
H	-4.135486	0.783285	1.498531
H	-4.507636	0.182188	-0.132245
H	-4.164719	-0.967447	1.181964
N	0.456752	1.419791	-0.785472
H	0.036830	2.313860	-1.008615
C	1.892835	1.259120	-0.977296
H	2.172265	1.905493	-1.809339
C	2.694204	1.609196	0.267947
H	2.431440	0.945741	1.092532
H	3.761223	1.496666	0.067080
H	2.508370	2.640129	0.574737
H	2.087354	0.232360	-1.283706
S	1.085358	-1.775158	0.424836
C	1.310571	-2.459166	-1.211470
C	2.463131	-3.213480	-1.420076
C	0.410601	-2.271833	-2.252505
C	2.706633	-3.777515	-2.662114
H	3.169317	-3.357919	-0.610386
C	0.665055	-2.837013	-3.494880
H	-0.487565	-1.689372	-2.096056
C	1.809840	-3.590884	-3.706541

H	3.604914	-4.363665	-2.814183
H	-0.043050	-2.686404	-4.300956
H	2.003203	-4.030153	-4.677153

4d

C	-1.924527	-1.224364	0.779707
C	-1.116774	-0.004874	0.634870
C	-1.650037	0.726222	-0.387031
C	-2.858155	-0.024519	-0.922745
N	-2.958699	-1.162907	-0.192702
O	-1.799994	-2.145325	1.543524
O	-3.565658	0.353737	-1.825617
C	-3.984332	-2.170129	-0.358702
H	-4.971815	-1.747549	-0.171395
H	-3.955931	-2.582290	-1.367452
H	-3.782514	-2.958025	0.363102
N	-1.355106	1.854403	-1.008863
H	-2.009713	2.110077	-1.737904
C	-0.278580	2.781623	-0.683160
H	-0.035949	3.315609	-1.602050
C	-0.655517	3.758061	0.421147
H	-0.875472	3.224873	1.346540
H	0.173821	4.442141	0.610773
H	-1.529896	4.347488	0.139397
H	0.600227	2.202877	-0.402225
S	0.220848	0.354289	1.699021
C	1.663095	-0.117583	0.747712
C	2.916085	0.186850	1.297869
C	1.569103	-0.749918	-0.485332
C	4.050709	-0.161860	0.573131
C	2.716444	-1.086723	-1.187930
H	0.596439	-0.982614	-0.897655
C	3.963269	-0.792367	-0.659727
H	5.024245	0.069810	0.990526
H	2.629543	-1.581908	-2.147460
H	4.864223	-1.051832	-1.201723
C	3.038933	0.869167	2.631288
H	2.532102	1.838007	2.630676
H	2.586783	0.272347	3.427996
H	4.085562	1.033241	2.885468

4e

C	-1.460043	-1.107981	0.032567
C	-0.081295	-0.597800	0.026290
C	-0.146506	0.758443	-0.100418
C	-1.611831	1.157060	-0.161884
N	-2.329140	0.007767	-0.083141
O	-1.856142	-2.243369	0.102412
O	-2.020962	2.288783	-0.266562
C	-3.772756	-0.077732	-0.123193
H	-4.212726	0.463006	0.715072
H	-4.155972	0.335245	-1.056653
H	-4.036005	-1.130750	-0.055002
N	0.718879	1.757682	-0.187602
H	0.292182	2.674662	-0.227118
C	2.169156	1.667591	-0.085515
H	2.577709	2.532490	-0.608597
C	2.654651	1.632794	1.356759
H	2.259665	0.758572	1.875767
H	3.744547	1.578480	1.383002
H	2.340952	2.529031	1.895060
H	2.496415	0.774977	-0.616554
S	1.299501	-1.661212	0.204593
C	1.406614	-2.377694	-1.489568
H	1.537357	-1.558822	-2.195708
H	0.462623	-2.880464	-1.692739
C	2.557321	-3.336412	-1.546424
C	2.383968	-4.669640	-1.183394
C	3.823145	-2.901936	-1.930717
C	3.453039	-5.553009	-1.211627
H	1.402563	-5.014034	-0.877305
C	4.893839	-3.783527	-1.960525
H	3.967770	-1.864657	-2.212225
C	4.710763	-5.111906	-1.600893
H	3.303617	-6.588515	-0.930580
H	5.872324	-3.433548	-2.266736
H	5.545622	-5.801717	-1.624949

4f

C	-1.704162	-1.114537	0.733138
C	-0.342381	-0.722704	0.343984
C	-0.391183	0.571220	-0.090152
C	-1.827853	1.053234	0.028771
N	-2.544317	0.011407	0.518517
O	-2.113814	-2.162948	1.157935
O	-2.215204	2.159039	-0.264755
C	-3.962123	0.039362	0.806683
H	-4.181967	0.761745	1.593416
H	-4.526951	0.300820	-0.088073
H	-4.244195	-0.956952	1.138927
N	0.463668	1.440688	-0.597268
H	0.062594	2.349909	-0.800144
C	1.909449	1.278097	-0.763325
C	2.642823	1.644405	0.521679
H	2.300754	1.024421	1.350972
H	3.716173	1.487937	0.398747
H	2.473966	2.693629	0.776033
H	2.085942	0.226625	-0.985053
S	1.013679	-1.813981	0.493593
C	1.333844	-2.328224	-1.188747
C	2.523721	-3.015696	-1.417148
C	0.472055	-2.069245	-2.246889
C	2.842266	-3.443282	-2.696261
H	3.201267	-3.212759	-0.594266
C	0.802415	-2.495886	-3.526071
H	-0.454278	-1.537426	-2.074912
C	1.984689	-3.183445	-3.757658
H	3.769241	-3.978263	-2.863875
H	0.124359	-2.289747	-4.345562
H	2.237520	-3.514289	-4.757184
C	2.355428	2.115369	-1.951679
H	2.156295	3.176615	-1.776825
H	3.427603	1.997840	-2.111014
H	1.835701	1.808486	-2.860301

4g

C	-1.532746	-1.080278	0.570945
C	-0.181162	-0.674970	0.134473
C	-0.275375	0.581230	-0.373785
C	-1.722972	1.015017	-0.297230

N	-2.405602	-0.005453	0.289552
O	-1.892237	-2.115567	1.066682
O	-2.154683	2.072627	-0.687987
C	-3.822302	0.000729	0.585358
H	-4.064999	0.796802	1.289657
H	-4.401704	0.139788	-0.327340
H	-4.065083	-0.962869	1.027117
N	0.555407	1.450736	-0.958951
H	0.092045	2.254157	-1.366973
S	1.156256	-1.799044	0.221752
C	1.813491	-1.728466	-1.438480
C	3.189257	-1.855200	-1.591144
C	0.999478	-1.579037	-2.555374
C	3.747557	-1.833248	-2.860184
H	3.821998	-1.947060	-0.717606
C	1.567300	-1.538655	-3.819089
H	-0.072612	-1.488805	-2.436913
C	2.941163	-1.666503	-3.976845
H	4.820720	-1.927165	-2.972549
H	0.929827	-1.414421	-4.686212
H	3.380557	-1.635275	-4.966174
C	1.969687	1.445348	-0.936808
C	2.662744	1.791932	-2.091313
C	2.657950	1.132824	0.229047
C	4.046599	1.811886	-2.080586
H	2.112165	2.021724	-2.995141
C	4.044845	1.141598	0.225729
H	2.110365	0.894558	1.130141
C	4.742242	1.478284	-0.924948
H	4.584546	2.074026	-2.983083
H	4.580549	0.890785	1.132914
H	5.825095	1.484708	-0.921974

4h

C	-1.825504	-2.618948	1.184467
C	-1.652685	-1.184862	1.460135
C	-2.639511	-0.508100	0.798054
C	-3.493218	-1.524210	0.052234
N	-2.951782	-2.737407	0.334084
O	-1.177824	-3.560174	1.559002
O	-4.441915	-1.252773	-0.639434
N	-3.008683	0.752924	0.659502
H	-3.796609	0.890541	0.038657
S	-0.424312	-0.597072	2.558693
H	-3.295404	-3.618004	-0.018277
C	-2.299713	1.921885	1.163248
C	-1.139479	2.332690	0.261869
H	-1.935234	1.696139	2.163994
H	-3.028314	2.728786	1.251038
C	-0.269807	3.412582	0.893604
H	-0.523453	1.454532	0.056122
H	-1.531253	2.677446	-0.700565
C	0.912682	3.792511	0.010490
H	-0.876775	4.299155	1.106067
H	0.098219	3.049147	1.858579
H	1.527587	4.563694	0.478835
H	1.549300	2.924555	-0.176305
H	0.574519	4.176000	-0.955758
C	0.986055	-0.281683	1.506458
C	2.004676	0.501601	2.042423
C	1.102684	-0.777550	0.214204
C	3.131367	0.785522	1.286328
H	1.912033	0.895635	3.047897
C	2.229942	-0.481130	-0.538993
H	0.313933	-1.385775	-0.207964
C	3.247949	0.299023	-0.009065
H	3.917165	1.398826	1.710422
H	2.311545	-0.868867	-1.547302
H	4.125579	0.527074	-0.600818

4i

C	-1.444259	-1.064144	0.289381
C	-0.079997	-0.551415	0.087099
C	-0.175032	0.776293	-0.210786
C	-1.645154	1.166148	-0.193263
N	-2.325431	0.028083	0.104786
O	-1.817161	-2.178519	0.548710
O	-2.070638	2.273146	-0.409509
N	0.666581	1.756406	-0.508228
H	0.226119	2.659499	-0.629685
C	2.120882	1.683959	-0.524518
H	2.469728	2.462620	-1.203223
C	2.733891	1.861626	0.857418
H	2.404222	1.068875	1.530115
H	3.822677	1.819408	0.791296
H	2.452076	2.824176	1.288156
H	2.411715	0.723130	-0.946100
S	1.328894	-1.576504	0.280265
C	1.337452	-2.468854	-1.318569
C	2.474301	-3.478059	-1.345705
H	1.445686	-1.737928	-2.121581
H	0.374826	-2.969140	-1.427140
C	2.531408	-4.246242	-2.663550
H	3.427335	-2.963419	-1.183390
H	2.354043	-4.183816	-0.517670
C	3.660324	-5.269963	-2.695466
H	1.573269	-4.749560	-2.828038
H	2.653396	-3.537873	-3.489507
H	3.684038	-5.806667	-3.645901
H	4.631799	-4.787541	-2.559877
H	3.542438	-6.007940	-1.897980
H	-3.328884	-0.042215	0.178246

4j

C	-1.056031	-2.800211	0.948325
C	-0.766376	-1.363568	1.049129
C	-1.872781	-0.683903	0.622595
C	-2.941774	-1.701525	0.251050
N	-2.381378	-2.920567	0.464042
O	-0.353045	-3.747272	1.189623
O	-4.044043	-1.430361	-0.154883
N	-2.221474	0.578953	0.451098
H	-3.172925	0.707629	0.126775
S	0.782091	-0.782151	1.619858
H	-2.841547	-3.803728	0.303874
C	-1.418262	1.767668	0.690809
C	-1.719412	2.834569	-0.348227
H	-0.370266	1.482607	0.648259
H	-1.615065	2.145124	1.700093
C	-0.890562	4.095097	-0.131968

H	-1.512523	2.423214	-1.339484
H	-2.785993	3.086874	-0.319389
C	-1.128020	5.141161	-1.214049
H	-1.120379	4.519470	0.850752
H	0.169449	3.824144	-0.111579
H	-0.528119	6.036764	-1.041494
H	-0.866311	4.747391	-2.199608
H	-2.177910	5.443639	-1.246081
C	1.815730	-1.000872	0.107702
H	2.821175	-0.728658	0.429401
H	1.811377	-2.060995	-0.140010
C	1.360642	-0.159264	-1.042545
C	1.798993	1.156473	-1.182143
C	0.456825	-0.665955	-1.973138
C	1.346181	1.946556	-2.227530
H	2.501589	1.562982	-0.462877
C	-0.003915	0.125033	-3.016803
H	0.117715	-1.691267	-1.883102
C	0.439689	1.433284	-3.147046
H	1.700621	2.965558	-2.326184
H	-0.707148	-0.283964	-3.732195
H	0.084477	2.050529	-3.963284

Table S5. Cartesian coordinates (in Å) of the stationary points in the first excited state optimized at the CAM-B3LYP-D3BJ/6-311G(d,p) level of theory in solution using the PCM with ε = 4.24.

1a

C	0.829009	1.213256	-0.002687
C	-0.499378	0.726026	0.000476
C	-0.510834	-0.698857	0.001399
C	0.810344	-1.205222	-0.000611
N	1.608053	-0.003504	-0.003239
O	1.327860	2.322275	-0.004021
O	1.295979	-2.320448	-0.000868
C	3.028490	-0.024280	-0.002670
H	3.380997	-0.552303	0.890557
H	3.380674	-0.601743	-0.864034
H	3.394210	0.997859	-0.028043
Cl	-1.857944	1.725027	0.002515
Cl	-1.884915	-1.676104	0.005371

1b

C	-1.381911	-1.154479	0.117363
C	-0.129822	-0.513612	-0.068133
C	-0.309262	0.894625	-0.200135
C	-1.685825	1.224310	-0.105358
N	-2.310315	-0.054985	0.088270
O	-1.723710	-2.312326	0.271560
O	-2.313386	2.266220	-0.156927
C	-3.717363	-0.210252	0.236802
H	-4.057805	0.378524	1.094932
H	-4.221529	0.197637	-0.645413
H	-3.939394	-1.265250	0.367550
Br	1.459612	-1.431646	-0.120765
Br	1.001647	2.154653	-0.456656

1c

C	-1.293653	-1.377218	0.137900
C	-0.288438	-0.382091	-0.050023
C	-0.902534	0.897793	-0.203192
C	-2.320216	0.759872	-0.117312
N	-2.510883	-0.635595	0.089440
O	-1.243471	-2.583706	0.306757
O	-3.239395	1.558216	-0.188181
C	-3.803436	-1.228167	0.232977
H	-4.324287	-0.768560	1.077694
H	-4.398447	-1.029314	-0.663050
H	-3.675614	-2.295892	0.386557
I	1.693608	-0.852586	-0.075769
I	-0.014524	2.706507	-0.501419

2a

C	1.456802	-0.155909	-0.850718
C	2.817098	0.249854	-1.112853
C	3.220943	1.249801	-0.162193
C	2.107973	1.478697	0.710702
N	1.093319	0.617000	0.250893
O	0.744868	-0.976911	-1.426405
O	2.025941	2.257151	1.668702
C	-0.202439	0.543207	0.871247
H	-0.110275	0.142890	1.883428
H	-0.634183	1.542720	0.938015
H	-0.825780	-0.109564	0.265969
N	4.349722	1.925449	-0.002163
H	4.324202	2.557111	0.791476
C	5.598732	1.794004	-0.736565
H	6.125105	2.741329	-0.623861
C	6.462666	0.643081	-0.230460
H	5.948803	-0.312406	-0.342888
H	7.391389	0.601105	-0.802296
H	6.711208	0.780956	0.822664
H	5.374484	1.671917	-1.795873
Cl	3.710714	-0.415136	-2.378085

2b

C	1.434745	-0.137752	-0.867999
C	2.799422	0.254799	-1.137448
C	3.219666	1.236028	-0.172494
C	2.115528	1.460686	0.713629
N	1.088536	0.618232	0.250390
O	0.706096	-0.939946	-1.449098
O	2.051624	2.225180	1.684524
C	-0.202391	0.546692	0.881625
H	-0.089095	0.245344	1.924607
H	-0.681030	1.527589	0.860189
H	-0.799185	-0.180035	0.337016
Br	3.732114	-0.477965	-2.538007
N	4.349385	1.909112	-0.002236
H	4.319856	2.525499	0.803533
C	5.602945	1.793175	-0.729239
H	6.116673	2.747685	-0.616000
C	6.479139	0.655063	-0.215008
H	5.978718	-0.307293	-0.329410
H	7.412722	0.624727	-0.779745
H	6.717826	0.798149	0.839789
H	5.385837	1.664352	-1.789177

2c

C	1.429934	-0.140736	-0.872836
C	2.797253	0.243041	-1.156680
C	3.221135	1.222753	-0.188423
C	2.120691	1.452552	0.701399
N	1.088309	0.615560	0.244452
O	0.695093	-0.942165	-1.447963
O	2.063938	2.220772	1.669966
C	-0.200053	0.549139	0.882100
H	-0.089462	0.202198	1.911435
H	-0.654483	1.541162	0.903274
H	-0.816659	-0.142328	0.314113
N	4.348676	1.901064	-0.010905

H	4.308515	2.515740	0.795876
C	5.608608	1.795052	-0.724983
H	6.111882	2.755510	-0.614231
C	6.494469	0.670060	-0.197940
H	6.001045	-0.297467	-0.297972
H	7.427194	0.638599	-0.764226
H	6.733903	0.828803	0.854385
H	5.403484	1.658305	-1.786519
I	3.774641	-0.598123	-2.731723

2d

C	2.491447	1.082513	0.158517
C	1.141999	0.746916	-0.265850
C	1.003257	-0.681918	-0.343445
C	2.265507	-1.260293	0.036984
N	3.086902	-0.159363	0.317056
O	3.006288	2.181016	0.341727
O	2.559695	-2.460254	0.102384
N	-0.009289	-1.459301	-0.692000
H	0.211248	-2.448583	-0.628016
C	-1.363548	-1.070993	-1.044192
H	-1.785512	-1.890161	-1.626630
H	-1.325374	-0.194388	-1.692579
C	-2.238008	-0.789265	0.178852
C	-3.635181	-0.330062	-0.222010
H	-1.760545	-0.022042	0.794767
H	-2.297982	-1.694737	0.789493
C	-4.521558	-0.044892	0.984636
H	-3.553940	0.570535	-0.839474
H	-4.102796	-1.094552	-0.850914
H	-4.643136	-0.937895	1.602567
H	-4.088284	0.737680	1.612365
Cl	-0.005209	1.933341	-0.590351
H	4.048459	-0.259594	0.615724
H	-5.514983	0.285961	0.676439

2e

C	2.518821	0.759623	0.342597
C	1.167388	0.487702	-0.123110
C	1.021427	-0.922940	-0.365139
C	2.280117	-1.548916	-0.052475
N	3.107764	-0.495166	0.356280
O	3.041980	1.823857	0.657219
O	2.566099	-2.750355	-0.128988
Br	-0.049789	1.840627	-0.322898
N	0.012525	-1.659319	-0.805261
H	0.241204	-2.647636	-0.855223
C	-1.345886	-1.246810	-1.105582
H	-1.760366	-1.992413	-1.784426
H	-1.318922	-0.295941	-1.640128
C	-2.220201	-1.127419	0.144034
C	-3.619631	-0.625681	-0.192248
H	-1.744131	-0.443767	0.852473
H	-2.276176	-2.103690	0.634169
C	-4.505137	-0.503379	1.042210
H	-3.542473	0.347704	-0.687758
H	-4.085365	-1.303689	-0.914769
H	-4.620797	-1.469480	1.539637
H	-4.074456	0.193470	1.765553
H	4.067148	-0.635571	0.645028
H	-5.500976	-0.140724	0.781107

3a

C	1.311612	0.992948	0.117095
C	-0.074331	0.755941	-0.124005
C	-0.314533	-0.656470	-0.300193
C	0.934439	-1.340117	-0.183817
N	1.872826	-0.299231	0.070923
O	1.945408	2.024325	0.315546
O	1.229169	-2.527972	-0.257281
C	3.262537	-0.553823	0.294511
H	3.432825	-0.784179	1.352440
H	3.559388	-1.419472	-0.295341
H	3.824366	0.341506	0.037542
C	-2.733997	-0.804047	-0.447948
H	-3.342981	-1.559138	-0.937881
C	-3.135072	-0.629280	0.999602
H	-2.520375	0.116265	1.504623
H	-4.173433	-0.296226	1.043841
H	-3.051750	-1.576008	1.533700
H	-2.797630	0.121204	-1.017464
Cl	-1.165143	2.040688	-0.229139
O	-1.393832	-1.343959	-0.557675

3b

C	-1.257240	-1.032585	0.167211
C	0.005955	-0.417086	-0.090992
C	-0.174603	0.996903	-0.320891
C	-1.570737	1.291616	-0.226261
N	-2.168793	0.036466	0.073869
O	-1.568461	-2.194980	0.406431
O	-2.194114	2.341128	-0.342885
C	-3.574869	-0.115169	0.293523
H	-3.810553	0.072142	1.347204
H	-4.104480	0.618032	-0.312263
H	-3.854702	-1.138195	0.051199
Br	1.525242	-1.449286	-0.178556
C	2.089003	1.871716	-0.443035
H	2.453183	2.756502	-0.958523
C	2.472196	1.890650	1.019795
H	2.098317	1.013429	1.548601
H	3.560344	1.896854	1.103445
H	2.082175	2.786786	1.502894
H	2.455215	0.987513	-0.961389
O	0.653671	1.964789	-0.608377

3c

C	1.015268	0.991457	0.223531
C	-0.296936	0.769896	-0.282724
C	-0.521016	-0.635254	-0.488164
C	0.661308	-1.343365	-0.122303
N	1.554911	-0.315717	0.299368
O	1.625223	2.010887	0.522694
O	0.925475	-2.539263	-0.085024
C	2.854511	-0.591713	0.825877
H	2.787120	-0.778580	1.904173
H	3.244543	-1.487974	0.346483
H	3.484832	0.280651	0.665270
C	-2.913548	-0.817146	-0.672178
H	-3.530499	-1.627379	-1.055876

H	-3.111659	0.079303	-1.257911
Cl	-1.319201	2.051151	-0.682105
O	-1.567274	-1.288314	-0.933091
C	-3.143376	-0.586935	0.795859
C	-3.924398	0.480807	1.219749
C	-2.590172	-1.450810	1.738042
C	-4.160200	0.681250	2.572995
H	-4.345016	1.164097	0.490951
C	-2.815888	-1.241895	3.089618
H	-1.974527	-2.281117	1.412233
C	-3.603480	-0.177456	3.509471
H	-4.768299	1.517494	2.895141
H	-2.378028	-1.914145	3.817127
H	-3.778588	-0.015705	4.565959

3d

C	-0.922253	-0.960612	0.230426
C	0.255168	-0.349709	-0.293151
C	0.030185	1.055267	-0.499287
C	-1.309824	1.364222	-0.116735
N	-1.837374	0.115018	0.315655
O	-1.188540	-2.117506	0.533160
O	-1.924989	2.423128	-0.070471
C	-3.153029	-0.021334	0.858952
H	-3.131485	0.161131	1.939634
H	-3.801445	0.720951	0.397016
H	-3.493713	-1.040652	0.688129
Br	1.713018	-1.361438	-0.768344
C	2.234473	2.009294	-0.668869
H	2.556853	2.969791	-1.066377
H	2.726298	1.216278	-1.229858
O	0.813079	2.005085	-0.952909
C	2.502867	1.896395	0.806240
C	3.591198	1.162981	1.261749
C	1.674375	2.539044	1.723141
C	3.857482	1.077822	2.621553
H	4.230620	0.649738	0.552733
C	1.934339	2.442561	3.081287
H	0.819896	3.106130	1.372550
C	3.027615	1.714289	3.532938
H	4.706795	0.502372	2.968588
H	1.282863	2.940219	3.788992
H	3.228937	1.639895	4.594514

3e

C	-2.151285	-0.777447	0.723442
C	-0.858597	-0.516399	0.185973
C	-0.808284	0.823748	-0.335006
C	-2.074845	1.443944	-0.142929
N	-2.846305	0.432648	0.501044
O	-2.634594	-1.759434	1.259596
O	-2.496458	2.552867	-0.419089
Cl	0.357896	-1.681773	0.214300
O	0.159790	1.500437	-0.910787
C	-4.201732	0.635949	0.905583
H	-4.720859	-0.320308	0.882252
H	-4.227227	1.017956	1.933151
H	-4.654072	1.378010	0.250659
C	1.429810	0.943428	-1.121830
C	1.677377	0.296130	-2.316166
C	2.398124	1.119666	-0.153417
C	2.946428	-0.220173	-2.533404
H	0.889191	0.197703	-3.051208
C	3.662307	0.597473	-0.385063
H	2.159317	1.648984	0.759798
C	3.935008	-0.075467	-1.568822
H	3.161374	-0.736428	-3.460332
H	4.435320	0.718574	0.363067
H	4.923305	-0.481192	-1.743728

3f

C	-2.270792	0.997230	-0.448480
C	-0.909769	0.683685	-0.748471
C	-0.649007	-0.681349	-0.404154
C	-1.842678	-1.281418	0.096239
N	-2.784941	-0.216148	0.067828
O	-2.930674	2.018047	-0.589330
O	-2.088923	-2.406946	0.507646
O	0.461250	-1.382988	-0.575062
C	-4.120888	-0.350471	0.563964
H	-4.754961	0.364829	0.043593
H	-4.447132	-1.378692	0.416950
H	-4.150848	-0.126019	1.636105
C	1.685401	-0.816249	-0.265491
C	2.761878	-1.186321	-1.056871
C	1.825319	0.040168	0.817788
C	4.006244	-0.652106	-0.773333
H	2.603895	-1.866836	-1.882801
C	3.078067	0.566278	1.086332
H	0.972739	0.274938	1.441983
C	4.166137	0.229261	0.291443
H	4.855794	-0.921054	-1.387880
H	3.205239	1.235356	1.927668
H	5.142447	0.643405	0.508095
Br	0.168948	1.830840	-1.698796

4a

C	-1.165162	-1.035499	-0.241640
C	0.101284	-0.334239	-0.352253
C	-0.160440	1.071515	-0.211270
C	-1.575294	1.249673	-0.050575
N	-2.117834	-0.034197	-0.092109
O	-1.412049	-2.250102	-0.232154
O	-2.197260	2.300212	0.078151
C	-3.522742	-0.297928	0.089430
H	-3.841375	0.007616	1.087990
H	-4.100969	0.266271	-0.643261
H	-3.678991	-1.365937	-0.038335
N	0.624223	2.142693	-0.298221
H	0.111287	3.014073	-0.226411
C	2.073089	2.187412	-0.254957
H	2.367291	3.179024	-0.596901
C	2.641561	1.914940	1.134943
H	2.353824	0.925730	1.494324
H	3.731764	1.960286	1.105294
H	2.282088	2.656897	1.849339
H	2.468443	1.464353	-0.973629
S	1.630302	-1.059495	-0.571787
C	1.280422	-2.553000	-1.562718

C	2.519735	-3.431777	-1.606961
H	0.996319	-2.206212	-2.557362
H	0.425015	-3.049829	-1.110074
C	2.290243	-4.674060	-2.464027
H	3.369201	-2.867750	-2.006468
H	2.790236	-3.734816	-0.591008
C	3.516853	-5.576678	-2.520685
H	1.438150	-5.232428	-2.064510
H	2.010841	-4.366932	-3.476886
H	3.331569	-6.457855	-3.137634
H	4.375334	-5.047864	-2.942822
H	3.796939	-5.921273	-1.522050

4b

C	7.838719	-1.534816	-0.311205
C	7.851654	-0.105884	-0.055362
C	9.191676	0.262770	0.312115
C	9.998752	-0.926130	0.321650
N	9.134350	-1.959571	-0.047761
O	6.929145	-2.273370	-0.711206
O	11.199919	-1.034364	0.610804
C	9.565638	-3.323033	-0.221137
H	9.982075	-3.473534	-1.220564
H	10.337175	-3.541708	0.514958
H	8.703453	-3.974211	-0.095954
N	9.735295	1.417865	0.692333
H	10.710518	1.328629	0.953871
C	9.155206	2.744427	0.620188
H	9.737525	3.379985	1.286489
C	9.149613	3.321805	-0.792599
H	8.575067	2.693313	-1.474814
H	8.697578	4.315409	-0.785715
H	10.166807	3.405527	-1.177856
H	8.136995	2.703728	1.015940
S	6.523975	0.954026	-0.218340
C	5.071082	-0.079713	0.175625
C	3.803026	0.665177	-0.208190
H	5.111624	-0.278765	1.247597
H	5.188167	-1.021117	-0.356609
C	2.556795	-0.137654	0.153608
H	3.768502	1.636490	0.296268
H	3.808371	0.866169	-1.283548
C	1.263673	0.582981	-0.210544
H	2.591082	-1.106815	-0.355360
H	2.562345	-0.352725	1.227938
H	1.232636	1.551189	0.302440
H	1.262594	0.804581	-1.283928
C	0.012432	-0.213798	0.140960
C	-1.282131	0.512783	-0.206154
H	0.020385	-0.446157	1.212311
H	0.038608	-1.177462	-0.380672
C	-2.535246	-0.283448	0.140303
H	-1.308241	1.474743	0.319166
H	-1.288455	0.749638	-1.276583
C	-3.829529	0.449853	-0.193586
H	-2.514030	-1.241501	-0.392152
H	-2.524182	-0.528380	1.208937
H	-3.850816	1.406500	0.341619
H	-3.839676	0.697977	-1.261496
C	-5.083698	-0.346144	0.149678
C	-6.377355	0.392697	-0.174307
H	-5.070140	-0.600215	1.216173
H	-5.066331	-1.299785	-0.390884
C	-7.632396	-0.402840	0.166856
H	-6.394658	1.345322	0.368170
H	-6.390314	0.648998	-1.240271
C	-8.925330	0.340412	-0.149845
H	-7.618122	-1.353209	-0.379569
H	-7.616990	-0.663488	1.231743
H	-8.939400	1.290092	0.397856
H	-8.940317	0.602574	-1.214361
C	-10.181175	-0.454441	0.189955
C	-11.473372	0.292318	-0.121486
H	-10.164458	-0.719743	1.253671
H	-10.169414	-1.402456	-0.360622
C	-12.729981	-0.501764	0.217484
H	-11.484842	1.239888	0.429894
H	-11.489796	0.558599	-1.184950
C	-14.021510	0.247519	-0.090194
H	-12.720143	-1.448143	-0.335969
H	-12.712292	-0.770246	1.280395
H	-14.031873	1.193850	0.463632
H	-14.039787	0.516819	-1.153027
C	-15.279724	-0.545254	0.248067
H	-15.269599	-1.489559	-0.306943
H	-15.259699	-0.815339	1.309471
C	-16.564375	0.215181	-0.061028
H	-17.449658	-0.372983	0.190003
H	-16.613012	1.149010	0.505478
H	-16.623578	0.469888	-1.122637

4c

C	-1.849411	-1.031692	1.006387
C	-0.590306	-0.938442	0.291124
C	-0.576240	0.309728	-0.432432
C	-1.850351	0.944082	-0.222306
N	-2.561278	0.103130	0.636755
O	-2.259454	-1.918149	1.757269
O	-2.231414	2.039349	-0.659180
C	-3.902501	0.381626	1.082525
H	-3.944991	1.378322	1.523775
H	-4.599309	0.346636	0.242645
H	-4.169584	-0.372903	1.818107
N	0.344592	0.996479	-1.111451
H	-0.008347	1.906892	-1.388358
C	1.763263	0.748684	-1.291789
H	2.108269	1.487135	-2.014652
C	2.569014	0.865178	-0.001079
H	2.247137	0.131389	0.738328
H	3.625323	0.689469	-0.213134
H	2.461794	1.860772	0.431590
H	1.911140	-0.231147	-1.744845
S	0.541263	-2.197365	0.486041
C	1.199721	-2.452339	-1.138647
C	2.537742	-2.824471	-1.252621
C	0.397199	-2.331480	-2.271851
C	3.074449	-3.060587	-2.507759
H	3.152922	-2.914377	-0.365825
C	0.949793	-2.558146	-3.521408
H	-0.647045	-2.066582	-2.170527

C	2.285707	-2.920248	-3.642956
H	4.114481	-3.346984	-2.599812
H	0.330215	-2.461310	-4.404013
H	2.710670	-3.099112	-4.622511

4d

C	-2.238772	-1.287599	0.952989
C	-1.142186	-0.372169	0.702483
C	-1.458699	0.397008	-0.475582
C	-2.708843	-0.095014	-0.989693
N	-3.121902	-1.092106	-0.103033
O	-2.384546	-2.105534	1.862906
O	-3.333103	0.309927	-1.980544
C	-4.335845	-1.847781	-0.277515
H	-5.190727	-1.171430	-0.327280
H	-4.296398	-2.420867	-1.205640
H	-4.433178	-2.518150	0.572710
N	-0.921570	1.461956	-1.074217
H	-1.502478	1.786671	-1.840317
C	0.162453	2.326154	-0.645638
H	0.379223	2.982055	-1.488109
C	-0.180293	3.156354	0.589132
H	-0.411547	2.520813	1.444540
H	0.670155	3.787237	0.855041
H	-1.040895	3.798073	0.395473
H	1.057479	1.730543	-0.469351
S	0.167774	-0.356951	1.792192
C	1.599174	-0.247871	0.749123
C	2.723563	0.445346	1.222317
C	1.606211	-0.877984	-0.494958
C	3.837422	0.501013	0.392144
C	2.726358	-0.795167	-1.302142
H	0.736088	-1.433209	-0.819574
C	3.843293	-0.100953	-0.858527
H	4.716085	1.034736	0.734502
H	2.729384	-1.281369	-2.269287
H	4.725107	-0.033358	-1.483265
C	2.733780	1.117665	2.566238
H	1.957239	1.882393	2.635178
H	2.556275	0.402560	3.373105
H	3.696681	1.593041	2.747675

4e

C	-1.204544	-1.032662	-0.459904
C	0.087662	-0.380559	-0.347695
C	-0.144886	1.010355	-0.066281
C	-1.564068	1.231846	-0.039924
N	-2.136721	-0.018887	-0.290590
O	-1.488575	-2.225419	-0.632457
O	-2.170461	2.296819	0.147067
C	-3.559135	-0.247413	-0.286049
H	-3.922115	-0.408151	0.732395
H	-4.055012	0.629568	-0.698455
H	-3.766825	-1.134678	-0.879815
N	0.678526	2.047034	0.078024
H	0.190085	2.926218	0.203636
C	2.120094	2.025577	0.231539
H	2.474580	3.034036	0.020922
C	2.569880	1.584574	1.621698
H	2.217216	0.577910	1.851700
H	3.660126	1.581320	1.675296
H	2.186214	2.264492	2.383686
H	2.543982	1.365279	-0.529867
S	1.604636	-1.151652	-0.472852
C	1.308269	-2.509408	-1.677153
H	1.203503	-2.037584	-2.653293
H	0.362598	-2.970112	-1.399666
C	2.468662	-3.455377	-1.630497
C	2.488982	-4.491044	-0.699301
C	3.543872	-3.305058	-2.501069
C	3.564497	-5.364103	-0.643375
H	1.653168	-4.612331	-0.019592
C	4.620640	-4.178921	-2.447043
H	3.533810	-2.502088	-3.229765
C	4.632730	-5.209477	-1.517646
H	3.567926	-6.169173	0.081247
H	5.449677	-4.055935	-3.133207
H	5.471849	-5.893138	-1.475820

4f

C	-1.865102	-1.052349	1.013287
C	-0.590562	-0.907588	0.335328
C	-0.569292	0.382426	-0.310453
C	-1.852735	0.994829	-0.093535
N	-2.576981	0.098212	0.695266
O	-2.286045	-1.985503	1.698725
O	-2.234082	2.111301	-0.472336
C	-3.930872	0.341178	1.122524
H	-3.986439	1.292597	1.653602
H	-4.599214	0.388774	0.260686
H	-4.222987	-0.476531	1.776486
N	0.363057	1.107551	-0.930400
H	0.014994	2.033515	-1.164775
C	1.801421	0.899990	-1.048463
C	2.494873	1.041848	0.306439
H	2.083800	0.346456	1.038826
H	3.560899	0.831124	0.200540
H	2.376598	2.057670	0.689247
H	1.968059	-0.107435	-1.426011
S	0.542156	-2.173902	0.471009
C	1.237760	-2.316516	-1.152227
C	2.577070	-2.686555	-1.260034
C	0.467867	-2.097525	-2.293235
C	3.148579	-2.818316	-2.514991
H	3.166890	-2.852605	-0.367003
C	1.055394	-2.219042	-3.541364
H	-0.577732	-1.835504	-2.199460
C	2.393533	-2.575551	-3.655810
H	4.189787	-3.102013	-2.601758
H	0.461564	-2.043802	-4.429567
H	2.846112	-2.670981	-4.634659
C	2.337148	1.887386	-2.073232
H	2.173327	2.916995	-1.743153
H	3.409569	1.743180	-2.205589
H	1.848607	1.748149	-3.038675

4g

C	-1.528247	-1.071031	0.714916
C	-0.257221	-0.785779	0.081456
C	-0.351605	0.506745	-0.529433

C	-1.702050	0.978771	-0.376712
N	-2.350368	0.009033	0.387864
O	-1.872583	-2.054805	1.369517
O	-2.196344	2.026264	-0.807266
C	-3.730672	0.109121	0.788463
H	-3.858365	0.914575	1.515127
H	-4.353211	0.325291	-0.079928
H	-4.014653	-0.838671	1.239125
N	0.527010	1.288256	-1.179708
H	0.076285	2.049099	-1.679523
S	0.932841	-2.014415	0.055945
C	1.750874	-1.779472	-1.502000
C	3.137563	-1.882397	-1.544699
C	1.019040	-1.573879	-2.670000
C	3.793454	-1.771849	-2.760383
H	3.697298	-2.030823	-0.630628
C	1.686147	-1.451693	-3.876448
H	-0.061061	-1.518414	-2.631092
C	3.071698	-1.551657	-3.924803
H	4.872972	-1.845545	-2.793836
H	1.120232	-1.290889	-4.785571
H	3.587666	-1.459023	-4.872289
C	1.919028	1.338163	-1.068667
C	2.662904	1.785935	-2.160994
C	2.563271	0.997744	0.121802
C	4.040004	1.870852	-2.066025
H	2.154443	2.044442	-3.081641
C	3.941116	1.083298	0.201782
H	1.981769	0.691388	0.980834
C	4.686947	1.514294	-0.889114
H	4.613051	2.210720	-2.919630
H	4.435822	0.823199	1.129462
H	5.765308	1.579895	-0.819224

4h

C	-2.335272	-2.874867	1.642833
C	-1.699813	-1.563266	1.617938
C	-2.358489	-0.766031	0.612193
C	-3.331760	-1.600061	-0.048562
N	-3.264636	-2.826378	0.615222
O	-2.088635	-3.849688	2.347472
O	-4.117720	-1.273560	-0.946433
N	-2.360661	0.537831	0.344577
H	-3.060551	0.784276	-0.349288
S	-0.363706	-1.254464	2.625131
H	-3.823560	-3.626480	0.353490
C	-1.699520	1.624781	1.037328
C	-0.698312	2.370878	0.159329
H	-1.210768	1.228032	1.925311
H	-2.473069	2.321509	1.377087
C	-0.068784	3.548803	0.894631
H	0.076752	1.679257	-0.173847
H	-1.212441	2.728896	-0.738633
C	0.953931	4.288259	0.040788
H	-0.854493	4.241941	1.212781
H	0.411614	3.185925	1.808736
H	1.390666	5.129449	0.582577
H	1.766453	3.621402	-0.256938
H	0.494632	4.680058	-0.870286
C	0.823450	-0.512254	1.530478
C	1.684503	0.453796	2.044912
C	0.943226	-0.939528	0.209393
C	2.660947	0.999931	1.228398
H	1.583619	0.779296	3.073044
C	1.919135	-0.379703	-0.598905
H	0.287158	-1.709923	-0.173471
C	2.776906	0.589235	-0.094234
H	3.328929	1.754355	1.624374
H	2.014711	-0.709507	-1.625674
H	3.539320	1.021511	-0.729828

4i

C	-1.171471	-1.071102	-0.316305
C	0.098032	-0.359329	-0.389790
C	-0.171015	1.039932	-0.201362
C	-1.594363	1.213500	-0.044153
N	-2.116132	-0.074544	-0.137198
O	-1.411788	-2.282551	-0.353243
O	-2.214015	2.273861	0.118584
N	0.607785	2.118884	-0.238845
H	0.088102	2.984753	-0.146558
C	2.055390	2.173779	-0.253910
H	2.330667	3.169545	-0.599596
C	2.681291	1.893637	1.109835
H	2.407951	0.902626	1.475356
H	3.769321	1.938781	1.034343
H	2.352499	2.632131	1.842214
H	2.424462	1.456505	-0.992776
S	1.634042	-1.070132	-0.591072
C	1.315002	-2.558796	-1.597767
C	2.553383	-3.440035	-1.607128
H	1.062167	-2.207354	-2.599352
H	0.444384	-3.054590	-1.173845
C	2.349573	-4.673423	-2.483559
H	3.417737	-2.874980	-1.971594
H	2.787127	-3.753914	-0.585427
C	3.573963	-5.580644	-2.504720
H	1.481344	-5.231672	-2.120391
H	2.108273	-4.355668	-3.502911
H	3.407208	-6.455346	-3.135982
H	4.448588	-5.051399	-2.891463
H	3.817032	-5.935417	-1.500000
H	-3.095997	-0.283468	-0.005600

4j

C	-1.039879	-2.151995	0.401470
C	-0.841055	-0.713518	0.501834
C	-2.116821	-0.079298	0.317823
C	-3.104478	-1.100959	0.074837
N	-2.388451	-2.296564	0.129372
O	-0.232419	-3.080675	0.531064
O	-4.314742	-0.953043	-0.139542
N	-2.472261	1.200607	0.282691
H	-3.449710	1.346709	0.053925
S	0.626964	0.084575	0.823245
H	-2.824615	-3.204258	0.045217
C	-1.646597	2.354205	0.570097
C	-2.447331	3.635723	0.418261
H	-0.784339	2.359638	-0.108398
H	-1.246326	2.276532	1.588151

C	-1.606173	4.875733	0.698512
H	-2.851265	3.686341	-0.598123
H	-3.302304	3.608382	1.101970
C	-2.401910	6.166778	0.548831
H	-1.195721	4.812053	1.711325
H	-0.748784	4.890859	0.018098
H	-1.781124	7.040891	0.753270
H	-2.798428	6.268148	-0.464537
H	-3.247917	6.188716	1.240344
C	1.907850	-1.010973	0.087675
H	2.836148	-0.627079	0.508400
H	1.714024	-2.015771	0.451498
C	1.894806	-0.936581	-1.410230
C	2.534366	0.109006	-2.072615
C	1.224960	-1.905506	-2.152263
C	2.504001	0.187292	-3.456189
H	3.060357	0.865151	-1.499865
C	1.195252	-1.825983	-3.537773
H	0.736025	-2.721217	-1.633727
C	1.830722	-0.779945	-4.192042
H	3.007872	1.002115	-3.961834
H	0.674827	-2.586184	-4.107688
H	1.805772	-0.719444	-5.273318

10. REFERENCES

1. M. P. Robin, A. B. Mabire, J. C. Damborsky, E. S. Thom, U. H. Winzer-Serhan, J. E. Raymond and R. K. O'Reilly, *J. Am. Chem. Soc.*, 2013, **135**, 9518-9524.
2. M. Dubernet, V. Caubert, J. Guillard and M.-C. Viaud-Massuard, *Tetrahedron*, 2005, **61**, 4585-4593.
3. R. Nirogi, A. Dwarampudi, R. Kambhampati, V. Bhatta, L. Kota, A. Shinde, R. Badange, P. Jayarajan, G. Bhyrapuneni and P. K. Dubey, *Bioorg. Med. Chem. Lett.*, 2011, **21**, 4577-4580.
4. A. B. Mabire, M. P. Robin, W.-D. Quan, H. Willcock, V. G. Stavros and R. K. O'Reilly, *Chem. Commun*, 2015, **51**, 9733-9736.
5. C. Würth, M. Grabolle, J. Pauli, M. Spieles and U. Resch-Genger, *Nat. Protoc.*, 2013, **8**, 1535-1550.
6. K. I. Booker-Milburn, J. R. Baker and I. Bruce, *Org. Lett.*, 2004, **6**, 1481-1484.
7. T. Yanai, D. P. Tew and N. C. Handy, *Chem. Phys. Lett.*, 2004, **393**, 51-57.
8. W. J. Hehre, R. Ditchfield and J. A. Pople, *J. Chem. Phys.*, 1972, **56**, 2257-2261.
9. W. J. Hehre, L. Radom, P. v. R. Schleyer and J. A. Pople, *Ab Initio Molecular Orbital Theory*, Wiley, New York, 1986.
10. S. Grimme, J. Antony, S. Ehrlich and H. A. Krieg, *J. Chem. Phys.*, 2010, **132**, 154104.
11. E. R. Johnson and A. D. Becke, *J. Chem. Phys.*, 2006, **124**, 174104.
12. S. Grimme, S. Ehrlich and L. Goerigk, *J. Comput. Chem.*, 2011, **32**, 1456-1465.
13. M. Cossi, N. Rega, G. Scalmani and V. Barone, *J. Comput. Chem.*, 2003, **24**, 669-681.
14. J. Tomasi, B. Mennucci and R. Cammi, *Chem. Rev.*, 2005, **105**, 2999-3093.
15. R. Bauernschmitt and R. Ahlrichs, *Chem. Phys. Lett.* 1996, **256**, 454-464.
16. M. E. Casida, C. Jamorski, K. C. Casida and D. R. Salahub, *J. Chem. Phys.* 1998, **108**, 4439-4449.
17. R. E. Stratmann, G. E.Scuseria and M. J. Frisch, *J. Chem. Phys.* 1998, **109**, 8218-8224.
18. C. Van Caillie and R. D. Amos, *Chem. Phys. Lett.* **1999**, *308*, 249-255.
19. C. Van Caillie and R. D. Amos, *Chem. Phys. Lett.* **2000**, *317*, 159-164
20. F. Furche and R. Ahlrichs, *J. Chem. Phys.* **2002**, *117*, 7433-7447.
21. G. Scalmani, M. J. Frisch, B. Mennucci, J. Tomasi, R. Cammi and V. Barone, *J. Chem. Phys.* **2006**, *124*, 094107.
22. C. Adamo and D. Jacquemin, *Chem. Soc. Rev.*, **2013**, *42*, 845-856.
23. A. D. Laurent, C. Adamo and D. Jacquemin, *Phys. Chem. Chem. Phys.*, **2014**, *16*, 14334-14356.
24. A. E. Reed, L. A. Curtiss and F. Weinhold, *Chem. Rev.*, **1988**, *88*, 899-926.
25. M. J. Frisch, G. W. Trucks, H. B. Schlegel, G. E. Scuseria, M. A. Robb, J. R. Cheeseman, G. Scalmani, V. Barone, B. Mennucci, G. A. Petersson, H. Nakatsuji, M. Caricato, X. Li, H. P. Hratchian, A. F. Izmaylov, J. Bloino, G. Zheng, J. L. Sonnenberg, M. Hada, M. Ehara, K. Toyota, R. Fukuda, J. Hasegawa, M. Ishida, T. Nakajima, Y. Honda, O. Kitao, H. Nakai, T. Vreven, J. A. Montgomery Jr., J. E. Peralta, F. Ogliaro, M. Bearpark, J. J. Heyd, E. Brothers,

K. N. Kudin, V. N. Staroverov, R. Kobayashi, J. Normand, K. Raghavachari, A. Rendell, J. C. Burant, S. S. Iyengar, J. Tomasi, M. Cossi, N. Rega, J. M. Millam, M. Klene, J. E. Knox, J. B. Cross, V. Bakken, C. Adamo, J. Jaramillo, R. Gomperts, R. E. Stratmann, O. Yazyev, A. J. Austin, R. Cammi, C. Pomelli, J. W. Ochterski, R. L. Martin, K. Morokuma, V. G. Zakrzewski, G. A. Voth, P. Salvador, J. J. Dannenberg, S. Dapprich, A. D. Daniels, Ö. Farkas, J. B. Foresman, J. V. Ortiz, J. Cioslowski and D. J. Fox, *Gaussian 09*, *revision E.01*, *Gaussian, Inc.: Wallingford, CT*, 2009.
